# *Elektra*-Qki and *Alien*-Wt1 lncRNA-protein interaction controls myocardial ion channel expression and epicardial EMT during heart development in mice

**DOI:** 10.1007/s00018-026-06322-x

**Published:** 2026-07-07

**Authors:** Sheila Caño-Carrillo, Estefanía Lozano-Velasco, Diego Franco

**Affiliations:** Cardiovascular Development Group, Department of Experimental Biology, Faculty of Experimental Sciences, University of Jaén, 23071 Jaén, Spain

**Keywords:** *Elektra*, *Alien*, LncRNA, Myocardium, Epicardium, Ion channels, EMT

## Abstract

**Background:**

The heart is the first organ to develop during embryogenesis and its development relies on coordinated interactions between the myocardium and the embryonic epicardium (EE), the third layer of the heart derived from the proepicardium (PE). A subset of epicardial cells undergoes an epithelial-to-mesenchymal transition (EMT), invades the subepicardial space and leads to epicardial-derived cells (EPDCs) colonizing the embryonic myocardium and differentiating into multiple cardiac lineages. However, the regulatory landscape governing these processes remains incomplete. In recent years, long non-coding RNAs (lncRNAs) have emerged as key regulators of cardiac development. Previous data from our laboratory identified two murine lncRNAs, *Elektra* and *Alien*, with differential expression during the transition from the PE to the EE.

**Methods:**

In this study, we performed a multi-omic characterization of both lncRNAs across embryonic and adult tissues. Their transcriptional regulation was evaluated through cardiogenic transcription factors analysis, while their molecular interactomes were identified via RNA pull-down (PD) and mass spectrometry (MS) assays and subsequently validated by RNA immunoprecipitation (RIP). Functional impact was analyzed through loss-of-function experiments and RT-qPCR, focusing on cell migration and EMT dynamics, evidencing distinct roles for each lncRNA.

**Results:**

*Elektra* regulated the expression of ion channel genes in the myocardium through interaction with Qki protein, while *Alien* modulates the epicardial EMT process by interacting with Wt1 and controlling EMT-related genes, including *Snai1*, *Snai2*, *Cdh1* and *Cdh2*.

**Conclusion:**

Altogether, our findings reveal that *Elektra* and *Alien* exert important roles in cardiac development by regulating myocardial ion channel expression and epicardial EMT, respectively, supporting new insights into lncRNA-mediated regulation of heart morphogenesis in mice.

**Supplementary Information:**

The online version contains supplementary material available at https://doi.org/10.1007/s00018-026-06322-x.

## Background

The formation of the heart is a complex developmental process that initiates soon after gastrulation with the configuration of the bilateral precardiac precursors that will subsequently fuse in the embryonic midline forming a cardiac straight tube [1, 2]. The linear cardiac tube, initially composed of myocardium and endocardium, begins to grow and elongate due to cardiomyocyte proliferation. This structure undergoes a rightward looping, which is the first evidence of asymmetry in heart development [3, 4]. Then, the third and final cellular layer of the heart, the embryonic epicardium (EE) originates from the proepicardium (PE), a transient cauliflower-structure located near the venous pole [5, 6]. This transient structure exhibits high cellular heterogeneity, as evidenced by the differential expression of several gene markers, suggesting the presence of distinct cell populations within the PE [7]. After PE formation, proepicardial cells delaminate and generate vesicles that float in the pericardial cavity until they reach the myocardium, where they adhere and begin to cover the myocardial surface [8]. Epicardial development is essential for correct cardiac morphogenesis, since once established, a cellular subset undergoes an epithelial-mesenchymal transition (EMT), giving rise to epicardial-derived cells (EPDCs) that invade the myocardium. These EPDCs can differentiate into various cell types, including smooth muscle cells, fibroblasts and endothelial cells [9–11]. Due to its importance in cardiac development, epicardial EMT is a highly regulated process, involving several signaling pathways as retinoic acid, Wnt and Hedgehog [12, 13]and transcription factors, such as *Wt1*, *Snai1* or *Snai2* [14, 15]. Once the embryonic heart has developed three distinct cardiac layers, each chamber is subsequently divided into right and left parts as development proceeds, resulting in the configuration of the fully formed four-chambered heart with distinct inlet and outlet connections [16].

The heart is the first organ that is functional during embryonic development [17, 18]. As soon as the cardiac straight tube is formed, a peristaltic contraction is initiated and blood is directionally pumped [19]. As development proceeds, the synchronous chamber contraction initiates and the configuration of the atrial and ventricular cardiac action potentials takes place, involving distinct sodium, calcium and potassium ion channels [20]. For efficient pumping of blood throughout the body, it is essential that the heart precisely coordinates its structural changes and functional responses. Alterations in the heart contractile capacity can lead to cardiovascular diseases such as cardiomyopathies or arrhythmias, including atrial fibrillation (AF), which can potentially result, eventually, in heart failure (HF) [21–23]. In particular, AF is the most common arrhythmias and its prevalence has significantly increased in recent years [24]. Mutations in sarcomeric genes can induce structural alterations in the atria and affect the expression of ion channels and other proteins involved in electrical conduction, resulting in arrhythmia development [25, 26]. In addition to sarcomeric genes such as *Myh7* [27] or *Myl4* [28], the transcription factor *Pitx2c* has been shown to play a key role in establishing AF. *Pitx2c* mutations have been associated with alterations in cardiac electrophysiology, including disruptions in calcium homeostasis, which can trigger arrhythmogenesis [29, 30]. Moreover, it has been elucidated that impairment of *Foxf-Wnt-Nr2f* regulatory network, which is evolutionarily conserved in both zebrafish and mice, may underlie congenital heart defects affecting the atrium, as well as the atrioventricular septum [31, 32].

In recent years, the mechanisms of transcriptional and post-transcriptional regulation have become more complex due to the discovery of non-coding RNAs (ncRNAs), which are involved in regulating multiple biological processes [33]. ncRNAs can be classified according to various criteria, including their biological function. In this context, the term “housekeeping RNAs” is used to describe a group that includes ribosomal RNAs (rRNAs), transfer RNAs (tRNAs), small nuclear RNAs (snRNAs) and small nucleolar RNAs (snoRNAs) [34, 35]. On the other hand, regulatory ncRNAs can be divided into two large groups according to their length [36, 37]. Small non-coding RNAs, which are less than 200 nucleotides, include microRNAs (miRNAs) [38]. These molecules, which consist of 19–25 nucleotides, exert a pivotal role in post-transcriptional control by binding to the 3’ UTR regions of messenger RNAs (mRNAs), thereby inducing their degradation and reducing their expression [39]. However, additional functions have recently been described at both nuclear and cytoplasmic levels, including transcriptional and translational regulation [40, 41]. In contrast, another group of regulatory ncRNAs are long non-coding RNAs (lncRNAs), which exceed 200 nucleotides and share several structural features with mRNAs [42]. Although the majority of lncRNAs are poorly conserved at sequence level among species, most of them are conserved at syntenic location levels [43]. Functionally, lncRNAs can act in both the nucleus and the cytoplasm, regulating processes such as transcription, alternative splicing, translation and/or protein distribution [44–47].

In the heart, several lncRNAs play a role in cardiac development by regulating key cellular mechanisms [33]. These include *Braveheart*, *CARMEN* or *Fendrr* lncRNAs, which are primarily involved in cardiac differentiation during the early stages of development [48–50]. Another relevant lncRNA is *ALIEN*, which is associated with the specification of cardiac mesoderm [51]. However, its function at later stages of development remains unclear. In addition to their role in differentiation, lncRNAs also regulate aspects of cardiac morphogenesis, including epicardial cell migration, formation of the cardiac chambers, trabeculation, or establishment of the conduction system and electrophysiology [52–54]. Dysregulation of these processes has been associated with multiple cardiac pathologies, including cardiomyopathies and electrical conduction disorders [55–57].

Using RNA-seq analysis, we recently identified two lncRNAs, *Elektra* (also known as *Gm35533*) and *Alien* (also known as *DEANR1*, *Falcor* or *9030622O22Rik*), which are differentially expressed between the PE and the EE [58]. In this study, we characterized these lncRNAs in the different cardiac cell types by analyzing their expression, subcellular distribution, and transcriptional regulation. Additionally, we identified key proteins that directly interact with these lncRNAs and are essential for epicardial development and cardiac electrophysiology regulation. Our results reveal that *Elektra* and *Alien* directly interact with Qki and Wt1, respectively, thereby regulating genes associated with voltage-gated ion channels in the myocardium, and also key genes involved in the epithelial-to-mesenchymal transition in epicardial cells. These findings suggest a potential regulatory role for these lncRNAs in essential cardiac processes such as myocardial contractility and epicardial development.

## Methods

### Mouse embryonic tissues

CD1 mice were bred and embryos were collected at different embryonic stages (E10.5, E13.5, E16.5, E19.5) as well as postnatal (P2, P7 and P21) and adult stages. Pregnant females were euthanized by cervical dislocation to obtain the embryos while neonatal mice were decapitated. For each embryonic and postnatal stage, the heart was carefully isolated, dissected and stored at −80°C until further used. To obtain epicardial cells, ventricles from E10.5 mouse embryos were isolated and cultured in 24-well plates for 48 h to allow the migration and expansion of epicardial cells. Additionally, tissues from the lung, stomach and liver were collected from embryonic (E13.5) and adult stages and stored following the same procedure. Approved consent of the Andalusian Ethic Committee was obtained prior to the initiation of the study (#09/07/2024/096 corresponding to the ALURES platform code NTS-ES-001583).

### siRNA and ASO transfections

HL1 cardiomyocytes (SCC065, Sigma Aldrich), MEVEC endocardial cells [59], MEC1 (SCC187, Sigma Aldrich) and EPIC epicardial cells [60] were cultured on 24-well plates (6 × 10^4^ cells per well) and transfected with *Pitx2c*-siRNA, *Mef2c*-siRNA, *Srf*-siRNA, *Nkx2.5*-siRNA, *Elektra*-siRNA, *Qki*-siRNA, *Wt1*-siRNA (Sigma, Aldrich, Munich, Germany), respectively. Antisense oligonucleotides (ASOs) were designed according to the method described by Le et al. [61] with a backbone of 10–12 DNA bases flanked by 5 modified 2’ O-Methyl RNA bases in both 5’ and 3’-arms. The sequences of siRNAs and ASOs are provided in Supplementary Table 1. Transfections were carried out using Lipofectamine 2000 (Invitrogen, Carlsbad, CA, USA) and Opti-MEM I (1X) (Gibco™, 31,985,070) following the manufacturer’s guidelines. siRNAs and ASOs concentration used ranged from 20 to 80nM and validation of the inhibition was carried out by RT-qPCR assays.

### Western blot

For protein knockdown validation, cells were lysed and subjected to Western blot analysis (WB). Membranes were blocked in milk powder-PBST for Qki and WT1, or milk powder-TBST for α-Tubulin according to the manufacturer’s recommendations. Membranes were then incubated overnight at 4 °C with the following primary antibodies: rabbit anti-Qki (1:1000; ThermoFisher, 1D1N6), rabbit anti-Wt1 (1:1000; ThermoFisher, 6F-H2), or mouse anti-α-Tubulin (1:5000, Sigma, T5168) as loading control. After washing, membranes were incubated for 30 min at room temperature with horseradish peroxidase (HRP)-conjugated secondary antibodies: anti-rabbit IgG (1:5000, Sigma, A0545) or anti-mouse IgG (1:5000, Sigma, A0168).

### Plasmid transfections

*Elektra* lncRNA was cloned into the pcDNA3.1 vector to achieve gain-of-function expression. To clone the ∼700 bp *Elektra* insert in sense orientation under the SP6 promoter, primers were designed with EcoRI and HindIII restriction sites at the 5’ ends of the forward and reverse primers, respectively. These enzymes do not cut within the insert, preserving its sequence integrity. The primer sequences used for cloning are detailed in Supplementary Table 1. HL1 cardiomyocytes were transfected with 300 ng of the plasmid using Lipofectamine 2000, as described above. Overexpression was validated by RT-qPCR assays.

### RNA isolation and cDNA synthesis

Total RNA isolation was carried out using ReliaPrep™ RNA Miniprep Systems (Promega, Z6011) according to the manufacturer's instructions. For RNA extraction from both tissue and cells, pooled samples were collected and stored at −80°C. First-strand cDNA synthesis for real-time RT-PCR (qPCR) was performed using PrimeScript™ RT Master Mix (Perfect Real Time) (Takara, RR036A) following the manufacturer's guidelines. For the analysis of lncRNAs and mRNA expression, a range of 100–500 ng total RNA was used for retrotranscription. Subsequently, the cDNA was diluted to a ratio of 1/3.5 for use in RT-qPCR. To avoid genomic DNA contamination, a negative control reaction was set up without reverse transcriptase enzyme, which produced no amplification signal during RT-qPCR.

### Nucleus/cytoplasm subcellular isolation

Nuclear and cytoplasmic RNA fractions were isolated from MEC1 and EPIC epicardial cells, MEVEC endocardial cells and HL1 cardiomyocytes using Cytoplasmic & Nuclear RNA Purification Kit (Norgen, Belmont, CA. USA, Cat. 21,000) according to the manufacturer’s instructions. Separation of RNA fractions from proteins or genomic DNA was achieved by column chromatography with Norgen resin. To verify the successful separation of nuclear and cytoplasmic RNA, the expression levels of nuclear-enriched mRNA (*Xist2*) and cytoplasmic-enriched mRNA (*Gapdh*) were analyzed by RT-qPCR in each fraction.

### qPCR analyses (mRNA and lncRNA)

Real-time PCR was performed using 2µL of cDNA (prepared as described above), 5 µL of GoTaq® qPCR Master Mix (Promega), and 1µL of specific primer (2.5µM). The reactions were conducted on a CFX384™ thermocycler (Bio-Rad) following the manufacturer's recommendations. The expression levels of the different mRNAs and lncRNAs were analyzed according to the ∆∆CT method described by Livak & Schmittgen [62] using *Gapdh* as an internal normalization control. Each reaction was prepared in triplicate using three biological samples. Negative control reactions, prepared without cDNA, showed no amplification signal. The sequences of all primers used are provided in Supplementary Table 1. Primer design was performed using Primer3Plus software (https://www.bioinformatics.nl/cgi-bin/primer3plus/primer3plus.cgi) and they were synthesized by IDT (Integrated DNA Technologies).

### SCRINSHOT in situ hybridization

SCRINSHOT *(Single-Cell Resolution IN Situ Hybridization On Tissues)* assay was performed as described by Sountoulidis et al. [63]. Hearts were collected at different embryonic (E10.5, E13.5, E16.5, E19.5) and postnatal stages (P2, P7, P21, P30) (*n* = 4). All samples were washed in PBS 1X (pH = 7.4), frozen in OCT (FSC22 Clear, Leica), and stored at −80 °C until used. Heart tissues were sectioned at a thickness of 5 µm using a cryostat (Leica CM3050S) and mounted onto Superfrost Plus slides. Padlock probes and detection oligos were designed using the IDT PrimerQuest™ Tool and RStudio. Padlock probes (ordered from IDT), were used at a concentration of 0.04 µM, while detection oligos (ordered from Eurofins), were employed at concentrations ranging from 0.02 µM to 0.01 µM. The sequences for both padlock probes and fluorophore-labeled detection oligos are provided in Supplementary Table 1. Images were captured using a Leica TCS SP5 II confocal scanning laser microscope. Subsequent image processing and analysis were performed with FIJI (v2.9.0), CellProfiler (v4.2.5), RStudio (v2024.04.2), and TissUUmaps (v3.0.10.1).

### LncRNA pull down assays

Biotinylated RNA pull-down was performed described by Panda et al*.* [64]. For the in vitro transcription of *Elektra and Alien*, primers were designed using Primer3plus adding the T7 RNA polymerase promoter sequence (T7) [5’AGTAATACGACTCACTATAGGG] upstream of the forward primer sequences. All primer sequences to obtain the DNA fragments are described in Supplementary Table 1. A biotinylated RNA negative control (*Gapdh*) was designed to validate the specificity of interaction between the RNA-binding proteins (RBPs) and the mRNA of interest. Two fragments were obtained for the *Elektra* sequence, seven fragments for *Alien* and one fragment for *Gapdh.* DNA fragments obtained by PCR were transcribed *in vitro* into RNA using MaxiScript T7 kit (Thermo Fisher Scientific, Invitrogen) and biotinylated with Biotin-14-CTP (Thermo Fisher Scientific, Invitrogen). Whole cells lysates (500µg) from MEC1 epicardial cells, MEVEC endocardial cells and HL1 myocardial cells were incubated with 1 µg of biotinylated RNA fragments and streptavidin-coupled dynabeads for 2 h at room temperature. A negative control sample (input), incubated without RNA fragments, was prepared to ensure specificity. All samples were processed in triplicate and analyzed by mass spectrometry (MS). Proteins uniquely associated with *Elektra and Alien* were selected and analyzed using the DAVID database (v2023q4).

### RIP assay

Ribonucleoprotein immunoprecipitation (RIP) assays for Qki and Wt1 proteins were respectively performed as previously described [65]. Whole-cell lysates from HL1 cardiomyocytes and MEC1 epicardial cells were prepared using PEB buffer (100 mM KCl, 5 mM MgCl2, 10 mM HEPES pH 7.0, 0.5% Nonidet P-40). Cells were lysed on ice for 10 min and centrifuged at 10 000 × g during 15 min at 4°C. For Qki RIP, the supernatant was incubated with protein A Sepharose beads (Abcam, ab193256) and antibodies recognizing Qki (ThermoFisher, 1D1N6) and IgG (Abcam, ab7085) proteins. For Wt1 RIP protein G Sepharose beads (Abcam, ab193259) and antibodies recognizing Wt1 (ThermoFisher, 6F-H2) and IgG (Santa Cruz, sc-52256) were used. Antibody-bead complexes were diluted in NT2 buffer (50 mM Tris, pH 7.4, 150 mM NaCl, 1mM MgCl2, 0.05% Nonidet P-40). The samples were incubated during 1–2 h at 4 °C and washed with NT2 buffer. To eliminate contaminating DNA and proteins, samples were treated sequentially with Ambion™ DNase I (Invitrogen, AM2222) and Proteinase K (20 mg/mL; Invitrogen, 17,916). Purified RNA was analyzed by RT-qPCR.

### Cell migration assay

Cell migration was evaluated using a scratch wound assay, following the protocol established by Ascione et al. [66]. MEC1 epicardial cells were seeded in 24-well plates at a density of 6 × 10^5^ cells per well and cultured until reaching 90–100% confluence. A uniform scratch was then made across the cell monolayer using a P200 pipette tip. After scratching, the cells were rinsed with PBS to remove debris, and the medium was replaced with serum-free culture medium. For the experimental condition, lipid vesicles loaded with antisense oligonucleotides (ASOs) were added, while control cells received empty lipid vesicles. Images were captured at 6 h, 12 h and 24 h after treatment to assess wound closure.

### Collagen gel assay for EMT analysis

To assess the ability of MEC1 epicardial cells and E10.5 ventricular explants to undergo EMT, cells were cultured on collagen gels in 24-well plates. Collagen gels were prepared using Corning™ Collagen I (rat tail, Fisher Scientific), 199 Medium, 1 M NaOH, and penicillin/streptomycin (P/S). The mixture was allowed to solidify by incubating the plates for 30 min at 37 °C. After polymerization, gels were washed three times with EMT medium consisting of high-glucose DMEM supplemented with GlutaMAX (ThermoFisher), Insulin-Transferrin-Selenium (1 ×) (Gibco), and P/S (1 ×). Following the specific treatments, gels were fixed and stained with DAPI (1:1000) for nuclear labeling and Phalloidin-iFluor 488 (1:1000) (Abcam, ab176753) to visualize F-actin filaments. Imaging was performed using a Leica TCS SP5 II confocal scanning laser microscope.

### Immunohistochemical analyses

HL1 cardiomyocytes were fixed with 4% paraformaldehyde (PFA) for 10 min at room temperature. After fixation, samples were washed twice with PBS for 5 min and incubated with permeation solution (50 nM NH_4_Cl, 0.2% Triton X-100, PBS 1X) during 10 min. Samples were washed twice with PBS 5 min each. Blocking was performed using 0.2% gelatin (Sigma-Aldrich, G1393) dissolved in PBS applied twice during 10 min. The primary antibody for Qki (ThermoFisher, 1D1N6) and Wt1 (ThermoFisher, 6F-H2) were diluted in blocking solution (1:200) andTODOO incubated at 4 °C overnight. Following primary antibody incubation, samples were washed three times with PBS and incubated with Alexa-Fluor 488-conjugated secondary antibody (Invitrogen) for 30 min at room temperature. Finally, cells were washed with PBS, incubated with DAPI (1:1000) for nuclei labelling and Phalloidin FITC (Abcam) (1:1000) for F-actin staining and stored in PBS until imaging using a Leica TCS SP5 II confocal scanning laser microscope. Negative controls were carried out without incubation of the corresponding specific primary antibody, and no signal was obtained in any of them after incubation with the secondary antibody.

### Statistical analyses

For statistical analyses of datasets, unpaired Student’s t-tests were used for comparisons between two groups. For multiple group comparisons, a one-way analysis of variance (ANOVA) was performed, followed by Tukey’s post-hoc test when all pairwise comparisons were of interest (Supplementary Table 2). When multiple experimental groups were compared with a common control group, a one-way ANOVA followed by Dunnett’s multiple-comparisons test was applied. A 95% confidence interval was maintained for all analyses. Significance levels of p-values are stated in each corresponding figure legend: * *p*-value < 0.05; ** *p*-value < 0.005; *** *p*-value < 0.001; **** *p*-value < 0.0001. GraphPad Prims software (v8.0.2) was used for statistical analysis and graphical representation.

## Results

### *Elektra* and *Alien* are developmentally expressed in multiple tissues displaying different cardiac expression profiles in adulthood

**Fig. 1:**
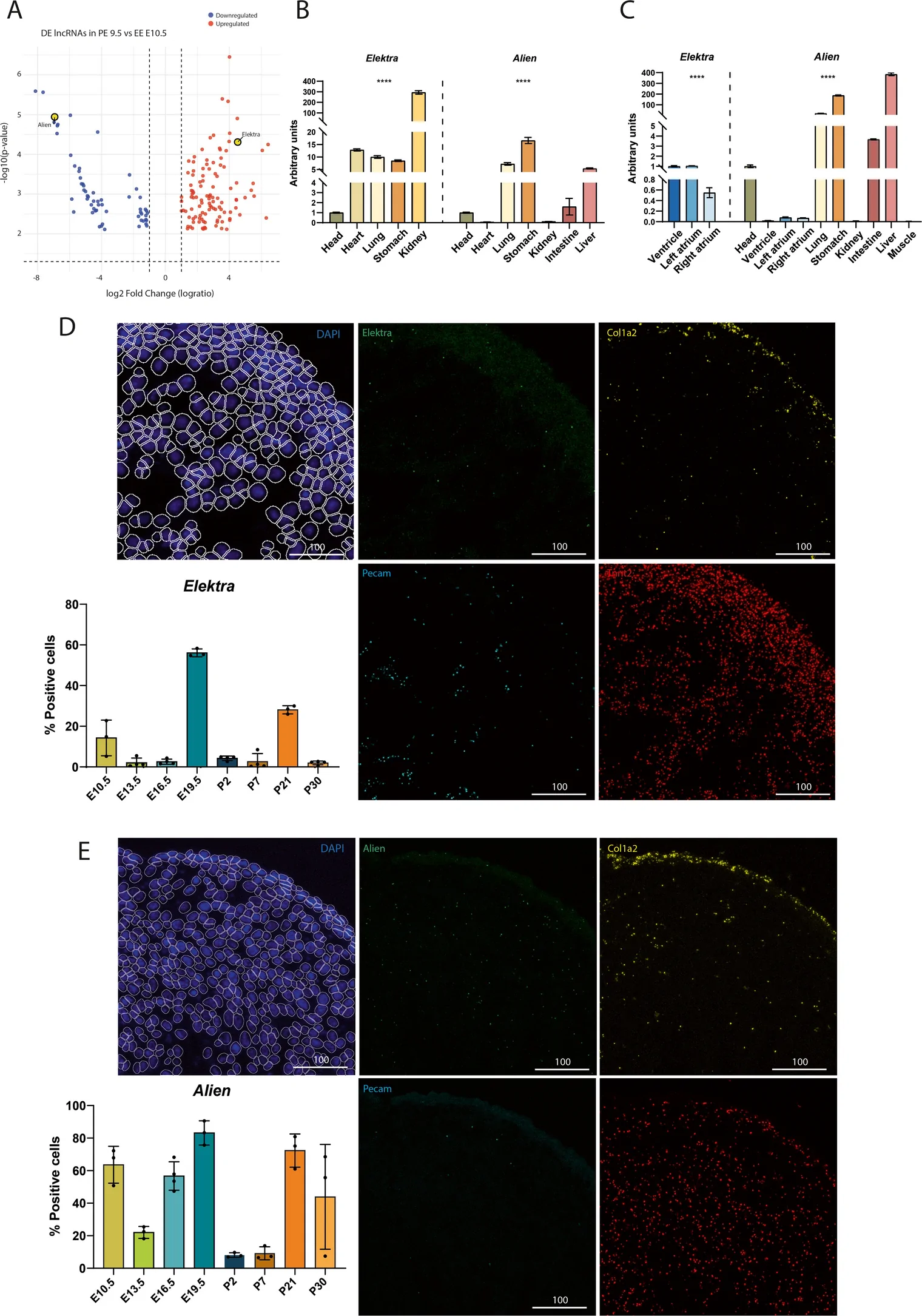
Expression and cellular distribution of *Elektra* and *Alien* lncRNAs during embryonic development and adulthood. Panel (**A**). Volcano plot showing differentially expressed lncRNAs identified by RNA-seq analysis of PE (E9.5) and EE (E10.5) samples. *Alien* and *Elektra* are highlighted as two of the most significantly altered lncRNAs, being downregulated and upregulated in EE, respectively. Panels (**B, C**). qPCR analysis showing tissue expression (*n* = 3) of *Elektra* and *Alien* at E13.5 (B) and adult stage (C). *Elektra* is highly expressed in the kidney at E13.5 and restricted to the heart in adults. *Alien* shows high expression in the lung, stomach and liver in both embryonic and adult stages. Panel (**D**). Percentage of *Elektra*-positive cells during cardiac development stages (E10.5 to P30) using SCRINSHOT analysis (*n* = 4), whose expression is higher during early embryogenesis and reduces over time with transient increases at postnatal day 2 (P2) and postnatal day 30 (P30). Panel (**E**). Percentage of *Alien*-positive cells during cardiac development stages (E10.5 to P30) (*n* = 4). *Alien* expression is higher at E13.5, decreases by early postnatal stages (P2 and P7), and reactivates at P21 and P30. The images provided in 1D and 1E correspond to E13.5 hearts. Statistical analysis: Fig. 1B-C**,** one-way ANOVA (95% confidence interval) followed by Tukey’s multiple-comparisons test. ANOVA pairwise comparison is provided in Supplementary Table 2; * *p*-value < 0.05; ** *p*-value < 0.005; *** *p*-value < 0.001; **** *p*-value < 0.0001. Schemes made with TissUUmaps software

To gain insight into the molecular changes associated with the transition from PE (E9.5) to EE (E10.5), we performed a comparative RNA-seq analysis of embryonic samples at these stages [53]. This analysis revealed a set of lncRNAs differentially expressed between the two conditions. Among them, we focused our attention on two particularly relevant lncRNAs, *Alien*, which was downregulated in EE, and *Elektra*, which was upregulated in EE at E10.5, as they were among the most significantly altered transcripts identified in this dataset (Fig. 1A). Based on these findings, we further characterize their expression patterns at two distinct developmental time points (E13.5 and adult) across various tissues and organs in mice. At E13.5, *Elektra* is highly expressed in the kidney, moderately expressed in the heart, lung and stomach and barely detectable in the head (Fig. 1B). Curiously, in adulthood, expression was exclusively confined to the heart, displaying similar expression levels in the ventricles and left atrium while it was significantly decreased in the right atrium (Fig. 1C). For *Alien,* high expression levels were observed in the embryonic lung, stomach and liver, with moderate levels in the head and intestine and lower levels in the heart and kidney (Fig. 1B). This expression pattern was largely maintained in adulthood, with elevated *Alien* levels persisting in the lung, stomach, intestine and liver. In contrast, its expression remained low in the kidney, skeletal muscle and the heart, where similar levels were found in the atria and the ventricle (Fig. 1C).

Although *Elektra* and *Alien* are not among the most highly expressed lncRNAs in cardiac tissue, we selected them for further analysis due to their potential involvement in heart development. To investigate their possible regulatory roles, we subsequently performed SCRINSHOT analyses to dissect their cellular distribution along cardiac development from E10.5 to E19.5 and postnatal stages from P2 to P30 (Fig. 1D, E). Globally, *Elektra* expression is more abundant at early developmental stages E10.5 corresponding to ∼ 10% of the total number of cells, decreasing as development proceeds (E13.5 and E16.5; corresponding to ∼ 2% of the total number of cells). Surprisingly a transient increase was observed at E19.5 (corresponding to ∼ 60% of the total number of cells), while becoming barely detectable at later postnatal stages (P2, P7 and P30) corresponding to ∼ less than 5% of the total cells (Fig. 1D). Notably, at P21, the proportion of *Elektra*-positive cells increased again to ∼ 27% of the total cell population. Overall these data illustrate that *Elektra* expression decreases during cardiac maturation, with transient increases at E19.5 and P21, suggesting an embryonic and postnatal dynamic regulation. We subsequently performed colocalization analyses with epicardial (*Col1a2*), endothelial (*Pecam*) and myocardial (*Tnnt2*) at different developmental stages (Supplementary Fig. 1A). Our data demonstrated that *Elektra* is expressed in all three distinct cell types, with comparable abundance on each of them at all developmental stages analyzed.

*Alien* expression levels were substantially higher than those of *Elektra*, but displayed a similar dynamic in embryonic and postnatal expression patterns (Fig. 1E). The highest expression levels were detected at E10.5, E16.5 and E19.5, with up to ∼ 85% of positive cells at E19.5. This was followed by a marked decrease in expression at early postnatal stages (P2 and P7), reaching ∼ 10% of total cells. Interestingly, at later postnatal stages (P21 and P30), expression increased again, obtaining ∼ 43% of positive cells at P30, comparable to the levels detected during embryogenesis. Furthermore, in line with the colocalization percentages, these data suggest that this lncRNA is expressed in the three cardiac cell types (Supplementary Fig. 1B). These results indicate a temporal expression pattern of *Alien*, with high embryonic levels, a marked decrease during early postnatal stages and a subsequent reactivation at late postnatal stages.

#### Subcellular distribution and transcriptional regulation of *Elektra* and *Alien*

**Fig. 2:**
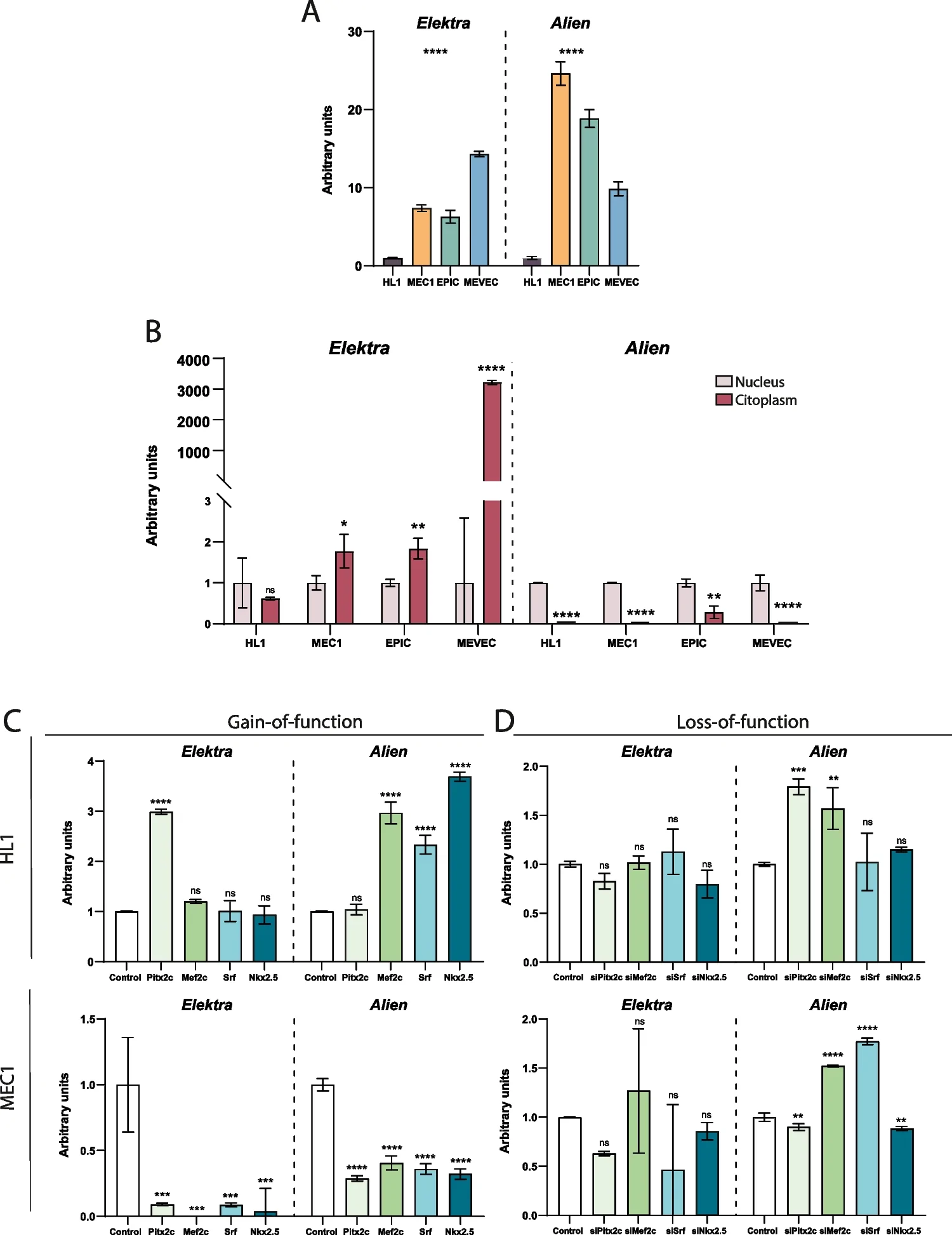
*Elektra* and *Alien* lncRNAs have distinct subcellular localization and cell-type-specific transcriptional regulation in cardiac cell lines. Panel (**A**). RT-qPCR analyses of *Elektra* and *Alien* expression in HL1 cardiomyocytes, MEC1 and EPIC epicardial cells and MEVEC endocardial cells. *Elektra* shows the highest expression in MEVEC endocardial cells. Followed by epicardial cells, and lowest in HL1 cells. In contrast, *Alien* is most highly expressed in epicardial cells. Panel (**B**). RT-qPCR analyses of nuclear and cytoplasmic distribution. *Elektra* shows variable localization depending on cell type, while *Alien* is mainly nuclear in all cell lines. Panel (**C**). RT-qPCR analyses of *Elektra* and *Alien* expression following overexpression of *Pitx2c*, *Mef2c*, *Srf* and *Nkx2.5* in HL1 and MEC1 cells (*n* = 3; each biological replicate corresponds to a single transfection assay). Panel (**D**). RT-qPCR analyses of *Elektra* and *Alien* expression following siRNA-mediated silencing of *Pitx2c*, *Mef2c*, *Srf* and *Nkx2.5* in HL1 and MEC1 cells (*n* = 3). These data reveal cell-type-specific transcriptional responses. Statistical analysis: Fig. 2A, one-way ANOVA followed by Tukey’s multiple-comparisons test; Fig. 2B, unpaired t-student test; Fig. 2C-D, one-way ANOVA followed by Dunnett’s multiple-comparisons test. A 95% confidence interval was used for all analyses. ANOVA pairwise comparison is provided in Supplementary Table 2; * *p*-value < 0.05; ** *p*-value < 0.005; *** *p*-value < 0.001; **** *p*-value < 0.0001

To further characterize *Elektra* and *Alien*, we subsequently analyzed their relative expression in different cardiac cell types, as well as their subcellular distribution. We examined the expression of both lncRNAs in HL1 atrial cardiomyocytes, MEC1 and EPIC epicardial cells and MEVEC endocardial cells, demonstrating that *Elektra* displayed the highest expression levels in endocardial cells, followed by epicardial cells, with the lowest levels observed in the myocardial cells (Fig. 2A). In contrast, *Alien* was most highly expressed in epicardial cells, followed by endocardial cells, and, similarly to *Elektra*, showed the lowest expression in cardiomyocytes (Fig. 2A). We further analyzed their subcellular distribution, after validating the correct nucleus-cytoplasm isolation (Supplementary Fig. 2A), and found that *Elektra* was equally distributed in the nucleus and cytoplasm in myocardial cells, prominently distributed in the cytoplasm in epicardial cells (both EPIC and MEC1) and almost exclusively cytoplasmic in endocardial cells (MEVEC) (Fig. 2B). These findings indicate that *Elektra* has a specific subcellular localization on each cardiac cell type, which supports the notion that this lncRNA can exert distinct functional roles in a cell-specific manner in the cardiac context. In contrast, *Alien* was mainly located in the nucleus in all three cardiac cells type (Fig. 2B), suggesting a conserved nuclear function across these cell types.

LncRNAs are transcriptionally regulated by RNA polymerase II in conjunction with distinct transcription factors as *Pitx2c*, *Mef2a/c*, *Tbx5*, *Gata4*, *Srf*, *Nkx2.5* and *Tead1*, as previously reported [67–75]. To investigate whether cardiac-enriched transcription factors modulate the expression of *Elektra* and *Alien*, we performed gain- and loss-of-function for different transcriptional factors, such as *Pitx2c*, *Mef2c*, *Srf* and *Nkx2.5* (Supplementary Fig. 2B), in distinct cardiovascular cell lines, such as HL1 atrial cardiomyocytes, MEC1 and EPIC epicardial cells and MEVEC endocardial cells. Focusing in *Elektra*, *Pitx2c* overexpression leads to downregulation in MEC1 and EPIC epicardial cells, as well as in MEVEC endocardial cells, while induces upregulation in HL1 cardiomyocytes (Fig. 2C and Supplementary Fig. 3A). On the other hand, *Pitx2c* inhibition resulted in *Elektra* downregulation in MEVEC endocardial cells while it has no effect on the other cell types (Fig. 2D and Supplementary Fig. 3B). *Mef2c* overexpression leads to *Elektra* downregulation in MEC1 and EPIC epicardial cells as well as in MEVEC endocardial cells, whereas it has no effect in HL1 cardiomyocytes (Fig. 2C and Supplementary Fig. 3A). In contrast, *Mef2c* silencing resulted in *Elektra* downregulation in MEVEC endocardial cells (Supplementary Fig. 3B). In HL1 cardiomyocytes and MEC1 and EPIC epicardial cells, no significant differences were identified (Fig. 2D, Supplementary Fig. 3B). Similarly, *Srf* overexpression downregulated *Elektra* in both MEC1 and EPIC epicardial cells as well as MEVEC endocardial cells, while no significant differences were detected in HL1 cardiomyocytes (Fig. 2C and Supplementary Fig. 3A), whereas *Srf* silencing caused upregulation in EPIC epicardial cells, downregulation in MEVEC endocardial cells and no significant effect in HL1 and MEC1 cells (Fig. 2D and Supplementary Fig. 3B). Finally, *Nkx2.5* overexpression downregulated *Elektra* in MEC1 and EPIC epicardial cells and MEVEC endocardial cells while no significant differences were observed in HL1 cardiomyocytes (Fig. 2C and Supplementary Fig. 3A), whereas *Nkx2.5* inhibition did not affect in HL1 cardiomyocytes and MEC1 and EPIC epicardial cells (Fig. 2D and Supplementary Fig. 3B). Collectively, these data demonstrate that *Elektra* is regulated by distinct cardiac-enriched transcription factors, displaying a cell type specific regulation, with all tested transcriptional factors playing a pivotal regulation role in EPIC epicardial cells, and *Pitx2c* overexpression particularly in HL1 cardiomyocytes.

Regarding the transcriptional regulation of *Alien*, *Pitx2c* overexpression reduced its expression levels in both EPIC and MEC1 epicardial cells, while no changes are observed in HL1 cardiomyocytes and MEVEC endocardial cells (Fig. 2C and Supplementary Fig. 3A). Conversely, loss of *Pitx2c* function resulted in increased *Alien* expression in HL1 cardiomyocytes and EPIC epicardial cells, decreased expression in MEC1 epicardial cells, and no changes in MEVEC endocardial cells (Fig. 2D and Supplementary Fig. 3B). *Mef2c* overexpression reduced *Alien* levels in MEC1 epicardial cells and in MEVEC endocardial, but upregulated it in HL1 cardiomyocytes, with no effect in EPIC epicardial cells (Fig. 2C and Supplementary Fig. 3A). In contrast, *Mef2c* inhibition upregulated *Alien* expression in HL1 cardiomyocytes and MEC1 and EPIC epicardial cells, while downregulation in MEVEC endocardial cells was observed (Fig. 2D and Supplementary Fig. 3B). In addition, overexpression of *Srf* reduced *Alien* levels in MEC1 epicardial cells, upregulated it in HL1 cardiomyocytes and MEVEC endocardial cells, and did not affect its expression in EPIC epicardial cells (Fig. 2C and Supplementary Fig. 3A). Conversely, *Srf* loss-of-function only increased *Alien* expression in MEC1 and EPIC epicardial cells, with no differences in the other cell types (Fig. 2D and Supplementary Fig. 3B). Notably, *Nkx2.5* overexpression reduced *Alien* expression in MEC1 epicardial cells and increased it in HL1 cardiomyocytes, while there were no significant differences in EPIC epicardial and MEVEC endocardial cells (Fig. 2C and Supplementary Fig. 3A). On the other hand, *Nkx2.5* inhibition upregulated *Alien* in MEVEC endocardial and EPIC epicardial cells, while downregulated it in MEC1 epicardial cells and there were no significant differences in HL1 cardiomyocytes (Fig. 2D and Supplementary Fig. 3B). Therefore, *Alien* is also regulated by cardiac transcription factors in a cell-type-specific manner, with *Pitx2c* exerting a significant influence in EPIC cells, while *Mef2c* and *Srf* predominantly regulate its expression in MEC1 cells. In summary, the differential responses observed across various cardiac cell types underscore the complexity of transcriptional regulation governing lncRNAs, *i.e. Elektra* and *Alien* in the cardiovascular context.

#### Proteomic analysis reveals cell-type-specific interactions of *Elektra* and *Alien* lncRNAs

**Fig. 3:**
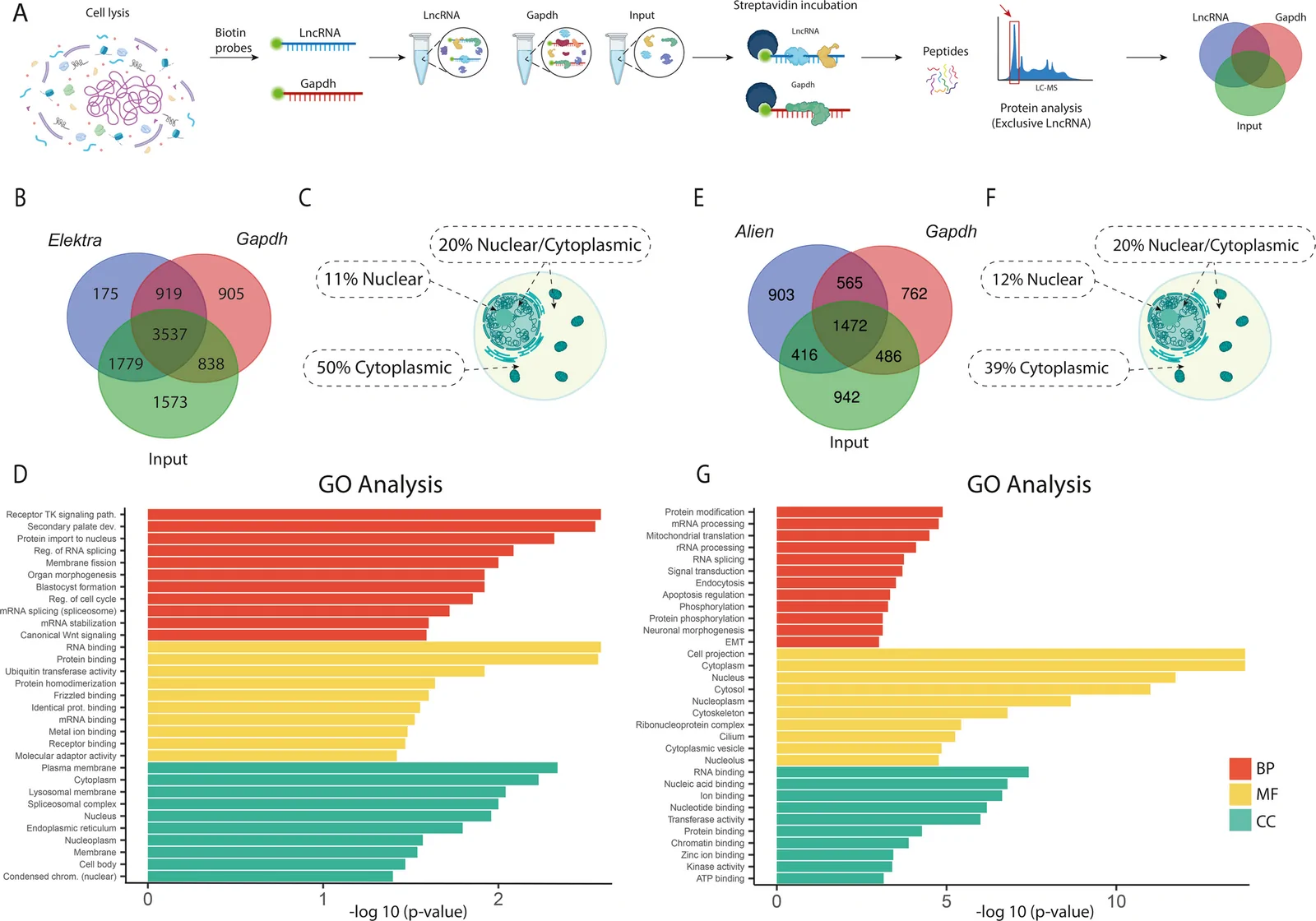
Distinct protein interaction networks of *Elektra* and *Alien* in cardiac cell types. Panel (**A**). Schematic representation of the RNA pull-down (PD) and mass spectrometry (MS) workflow used to identify lncRNA-associated proteins in HL1 cardiomyocytes, MEVEC endocardial cells and MEC1 epicardial cells. PD experiments were performed in biological triplicates (*n* = 3). Panel (**B**). Number of unique *Elektra*-associated proteins identified in HL1 cells compared to *Gapdh* and Input conditions. Panel (**C**). Subcellular classification of *Elektra*-binding proteins in HL1 cells, showing enrichment in the cytoplasm. Panel (**D**). GO enrichment analysis of *Elektra* interactors in HL1 cells, highlighting RNA binding, RNA splicing, ion channel regulation and membrane-related functions. Panel (**E**). Number of unique *Alien*-associated proteins identified in MEC1 epicardial cells compared to *Gapdh* and Input conditions. Panel (**F**). Subcellular distribution of *Alien*-associated proteins, with a predominance in the cytoplasm. Panel (**G**). GO analysis of *Alien* interactors in MEC1 epicardial cells, showing enrichment in EMT, cytoskeletal remodeling, RNA processing and mitochondrial translation. Schemes were created using Biorender (https://www.biorender.com/)

LncRNAs can exert multiple functions interacting with different types of molecules, including other RNAs species and protein [76]. Therefore, dissecting lncRNA-protein interactions can provide hints of their putative transcriptional and/or post-transcriptional functional role [44, 77]. In order to get further insights into the molecular mechanisms driven by *Elektra* and *Alien* lncRNAs in the cardiac context, we performed RNA pull-down (PD) assays followed by mass spectrometry (MS) to identify associated proteins with both lncRNAs, respectively (Fig. 3A). Since both lncRNAs showed cell type-dependent expression and specific transcriptional regulation, PD experiments were performed in different cardiac cell lines (HL1 cardiomyocytes, MEVEC endocardial cells and MEC1 epicardial cells for *Elektra*; and MEC1 epicardial cells for *Alien*) to identify proteins potentially associated with each lncRNA in different cellular contexts.

In epicardial cells, a total of 71 unique proteins that interact directly with *Elektra* were identified in MEC1 epicardial cells, as compared with *Gapdh* and Input pull-down control (Supplementary Fig. 4A). Approximately 32% of these proteins were exclusively nuclear, ∼ 22% were uniquely cytoplasmic and ∼ 32% were detected in both compartments (Supplementary Fig. 4B, Supplementary Table 3). Gene Ontology (GO) analysis of these proteins revealed an enrichment of biological processes (BPs) and molecular functions (MFs) associated with chromatin organization, nucleosome assembly, protein heterodimerization, and cytoplasmic translation (Supplementary Fig. 4C). These results indicate a variety of protein interactions involving *Elektra* in MEC1 epicardial cells. Supporting this, GO analyses of cellular components (CCs) identified the nucleus, nucleoplasm, cytosol and ribosomes as associated compartments (Supplementary Fig. 4C), suggesting a dual localization of *Elektra* within both nuclear and cytoplasmic regions.

A total of 29 unique *Elektra*-associated proteins were identified in MEVEC endocardial cells (Supplementary Fig. 4D), the majority of which were exclusively cytoplasmic (∼ 38%), located only in the nucleus (∼ 21%), or found in both compartments (∼ 21%) (Supplementary Fig. 4E, Supplementary Table 4). These percentages are in line with the GO analysis of cellular components (CCs), where the most representative compartment were the cytosol, ribosomes and cytoplasm, and with a lower proportion, the nucleus (Supplementary Fig. 4F). The major biological processes (BPs) and associated molecular functions (MFs) included cytoplasmic translation, rRNA processing, transcriptional regulation by RNA polymerases I and III, and protein binding (Supplementary Fig. 4F). These results suggest a more significant presence and potential function for *Elektra* in the cytoplasm, due to the high number of associated proteins found in this compartment.

In the myocardial context, a total of 175 proteins were uniquely identified in the *Elektra* PD assays, as compared to Input and *Gapdh* PD controls in HL1 cardiomyocytes (Fig. 3B, Supplementary Table 5). Approximately 11% were exclusively nuclear, ∼ 50% were exclusively cytoplasmic and ∼ 20% were present in both compartments (Fig. 3C). Gene ontology (GO) analyses of uniquely identified proteins in *Elektra* PD were primarily involved in biological processes (BPs) such as cell surface receptor protein tyrosine kinase pathway, secondary palate development, protein import into nucleus, regulation of RNA splicing membrane fission and animal organ morphogenesis among the most significant pathways (Fig. 3D). These results suggest the prominent role of *Elektra* in association with cytoplasmic biological processes, in line with its predominant cytoplasmic localization, although distinct nuclear and cytoplasmic biological functions are also identified. GO analyses of cellular components (CCs) revealed that the plasma membrane, cytoplasm, lysosomal membrane, spliceosomal complex and nucleus are the most representative compartments (Fig. 3D). Finally, GO molecular function (MF) analysis identified RNA binding, protein binding, ubiquitin-protein transferase activity and protein homodimerization activity as the most represented functions (Fig. 3D). Thus, all GO analyses support a dual nuclear and cytoplasmic role for *Elektra* in myocardial cells.

Comparative analyses of PD and MS data from MEC1, MEVEC and HL1 cells were performed to determine whether *Elektra* binds to similar proteins in different cardiac cell types. Curiously, we did not identify any proteins in common (Supplementary Fig. 4G), supporting the idea that lncRNA-protein interactions and their functions are cell-type-specific. Furthermore, despite the lower expression of *Elektra* in HL1 cardiomyocytes compared to the other cell types, the highest number of *Elektra*-associated proteins was detected in these cells, suggesting a higher functional relevance of the interaction in HL1 cardiomyocytes. Therefore, further functional analyses were performed in the myocardial context. We subsequently focused our attention on those cellular compartments and molecular and biological functions with a higher number of uniquely identified proteins in *Elektra* PD in HL1 cardiomyocytes, *i.e.* plasma membrane, cytoplasm, RNA binding, protein binding, protein import to the nucleus and regulation of RNA splicing (Fig. 3D). Among these categories, several families of proteins are highly represented, such distinct ion channels (e.g. Cacnb4, Kcnn3, KCa2.3), canonical Wnt signaling (Wnt8, Wnt11) and splicing mRNA stability (Qki, Snrnp70, Snrpd2). These findings suggest therefore that *Elektra* might be involved in RNA binding and regulation of RNA splicing, plausible playing a role in ion channel expression and/or remodeling.

For *Alien* lncRNA, a total of 903 proteins that specifically interact with this lncRNA were identified in MEC1 epicardial cells, after comparison with the Input and *Gapdh* PD controls (Fig. 3E, Supplementary Table 6). Approximately 12% of the total enriched proteins were classified as exclusively nuclear, ∼ 39% as exclusively cytoplasmic and ∼ 20% as localized in both nuclear and cytoplasmic compartments (Fig. 3F).

Gene Ontology (GO) analyses revealed that the most prevalent biological processes (BPs) include protein modification, mRNA processing, mitochondrial translation, and most notably, epithelial-mesenchymal transition (EMT) (Fig. 3G). In terms of molecular functions (MFs), *Alien*-associated proteins are mainly related to nucleic acid binding, ion binding, and RNA binding. Furthermore, the most representative cellular components include cell projections, the cytoplasm and the nucleus. Taken together, these results suggest that *Alien* may exert a functional role at both nuclear and cytoplasmic levels in MEC1 epicardial cells, possibly predominating in the cytoplasm due to the higher representation of cytoplasmic proteins. In addition, among the biological processes identified, the presence of key proteins involved in the maintenance and remodeling of the cytoskeleton (*e.g.* Krt39, Krt33a, Pacsin3, Pkp4), as well as the regulation of EMT (including factors such as Wt1, Tgfb1, Tgfb1r and Wnt11), is noteworthy. These findings may indicate a possible direct involvement of *Alien* in these cellular processes in epicardial cells.

#### *Elektra* modulates the gene expression of the cardiac action potential determinants by interacting with Qki and *Pitx2 > Wnt* signaling

Since *Elektra* is dually expressed in both nuclear and cytoplasmic subcellular compartments in cardiomyocytes, we designed siRNAs and ASO against this lncRNA to distinctly target its cytoplasmic (siRNA) and nuclear (ASO) molecular function. Additionally, we cloned *Elektra* into an expression vector, providing therefore a mean to overexpress this lncRNA. We subsequently tested the molecular function of *Elektra* loss-of-function and gain-of-function in the most significantly signaling pathways identified by MS/GO analyses, i.e. splicing/mRNA stability, cardiac ion channel and *Wnt* signaling.

**Fig. 4:**
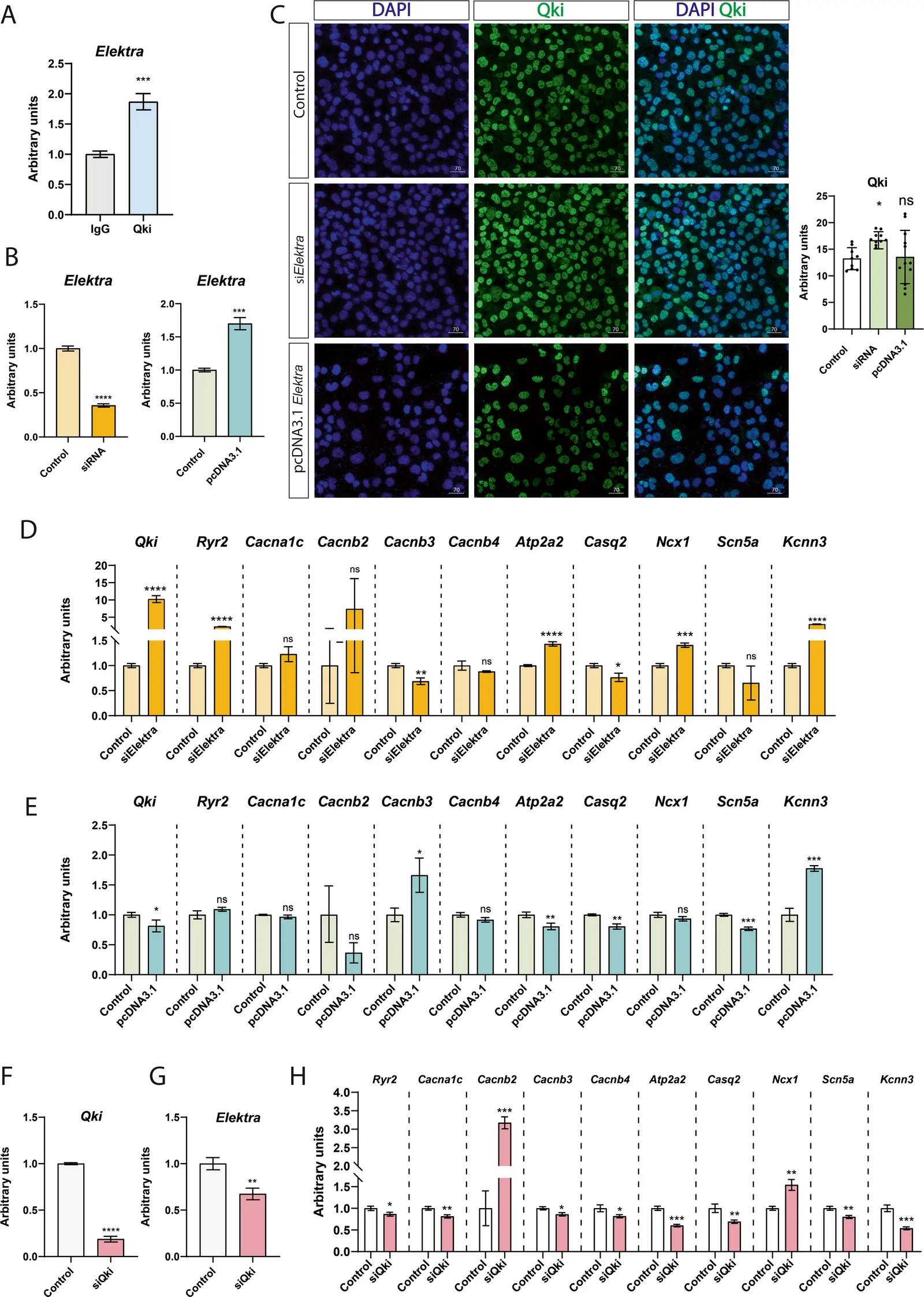
*Elektra* silencing alters cardiac ion channel expression through *Qki*-mediated regulation. Panel (**A**). RT-qPCR analyses of *Elektra* enrichment in Qki RIP assays from HL1 cardiomyocytes compared to IgG control. Panel (**B**). RT-qPCR validation of *Elektra* silencing and overexpression using siRNA and pcDNA3.1 vector, respectively, in HL1 cells (*n* = 3). Panel (**C**). Representative images for immunofluorescence analysis of Qki protein levels after *Elektra* loss- and gain-of-function. Increased Qki levels are observed upon *Elektra* inhibition (*n* = 3). Panel (**D**). RT-qPCR analyses of calcium, sodium and potassium channel gene expression in HL1 cells following *Elektra* silencing (*n* = 3). Notably, lncRNA knockdown leads to upregulation of *Qki*, *Ryr2*, *Atp2a2* and *Ncx1*, and downregulation of *Cacnb3* and *Casq2*. Panel (**E**). RT-qPCR analyses of calcium, sodium and potassium channel gene expression in HL1 cells following *Elektra* overexpression (*n* = 3). *Elektra* overexpression causes opposite effects for some genes. Both treatments increased *Kcnn3* expression. Panel (**F**). RT-qPCR analysis for *Qki* silencing validation in HL1 cardiomyocytes (*n* = 3). Panel (**G**). RT-qPCR analysis showing that knockdown of *Qki* leads to a significant decrease in *Elektra* expression levels in HL1 cardiomyocytes (*n* = 3). Panel (**H**). RT-qPCR analyses of gene expression changes in cardiac ion channel-related genes following *Qki* inhibition in HL1 cardiomyocytes. Silencing *Qki* led to upregulation of *Cacnb2* and *Ncx1*, and downregulation of *Ryr2*, *Cacna1c*, *Cacnb3*, *Cacnb4*, *Atp2a2*, *Casq2*, *Scn5a* and *Kcnn3* (*n* = 3). Statistical analysis: Fig. 4A-B, D-H unpaired t-student test; Fig. 4 C, one-way ANOVA followed by Dunnett’s multiple-comparisons test. A 95% confidence interval was used for all analyses. * *p*-value < 0.05; ** *p*-value < 0.005; *** *p*-value < 0.001; **** *p*-value < 0.0001

Previous studies have demonstrated that Qki depletion in embryonic or adult hearts, impairs cardiomyocyte contractility by disrupting the alternative splicing of key myofibrillar components (e.g., *Actn2* and *Ttn*) and calcium channels (e.g., *Cacnb1* and *Ryr2*) [78]. Given that our GO analysis of the *Elektra* dataset revealed a significant enrichment of both ion channels proteins and Qki itself, we first validated the direct interaction between *Elektra* and the Qki protein by RIP. We observed a significant enrichment of *Elektra* upon immunoprecipitation with Qki-specific antibody compared to the IgG control (Fig. 4A). Then, we validated gain- and loss-of-function of *Elektra* (Fig. 4B) and fluorescent immunohistochemistry assays revealed that *Elektra* inhibition results in Qki upregulation, whereas lncRNA overexpression did not significantly alter Qki protein levels (Fig. 4C). However, at the mRNA level changes were observed in both conditions: loss of *Elektra* function led to increased Qki expression (Fig. 4D), while gain of *Elektra* function reduced Qki levels (Fig. 4E).

In addition, inhibition of *Elektra* by siRNA lead to upregulation of genes coding for calcium handling proteins such as *Ryr2*, *Atp2a2* and *Ncx1*, downregulation of *Cacnb3* and *Casq2*, while no significant differences were observed for *Cacna1c, Cacnb2* and *Cacnb4* (Fig. 4D). On the other hand, overexpression of *Elektra* lead to downregulation of *Atp2a2* and *Casq2*, upregulation of *Cacnb3*, while no significant differences were observed for *Ryr2*, *Cacna1c*, *Cacnb2*, *Cacnb4* and *Ncx1* (Fig. 4E). Curiously, both inhibition and overexpression of *Elektra* leads to upregulation of the potassium channel *Kcnn3*, while for the sodium channel *Scn5a*, the silencing of *Elektra* does not alter its expression and the overexpression significantly decreases *Scn5a* expression (Fig. 4D-E). These results suggest that the effects observed upon siRNA-mediated silencing of *Elektra* are predominantly linked to the loss of its cytoplasmic function.

Since *Elektra* is expressed in the nucleus and cytoplasm of HL1 cardiomyocytes, the loss of function of this lncRNA was performed using an antisense oligo (ASO) to explore the functional analysis of *Elektra* in both compartments. ASO-mediated silencing of *Elektra* was evaluated at two time points: 6 h post-transfection, when the lncRNA was efficiently inhibited, and 24 h, when its expression was not significantly suppressed (Supplementary Fig. 5A-B). Thus, *Elektra* inhibition resulted in the upregulation of calcium channels genes such as *Cacnb3*, *Casq2* and *Ncx1;* sodium channel *Scn5a*, and potassium channels *Kcnn3* and *Kcnk3*. In contrast, *Ryr2* and *Cacnb4* expression was downregulated, while the levels of *Cacna1c*, *Cacnb2* and *Atp2a2* were no modified (Supplementary Fig. 5A). Furthermore, 24 h after ASO transfection, when *Elektra* expression was not modified, reduced levels of *Qki*, *Ryr2*, *Cacn1c*, *Cacnb2*, *Cacnb4*, *Atp2a2*, *Ncx1*, *Scn5a* as well as an upregulation of *Cacnb3* were identified. However, no changes in the expression levels of *Casq2*, *Kcnn3* and *Kcnk3* were observed (Supplementary Fig. 5B). Both inhibition methods of *Elektra*, siRNA and ASO, affect the expression of genes related to ion channels, showing both similarities and differences that suggest specific functions in different cellular compartments. Notably, *Ncx1* is upregulated after lncRNA inhibition by both approaches. siRNA, which primarily acts in the cytoplasm, causes a *Cacnb3* and *Casq2* downregulation, along with *Ryr2* and *Atp2a2* upregulation. In contrast, ASO inhibition, which affects both the nucleus and cytoplasm, induces upregulation of *Cacnb3*, *Casq2*, *Kcnn3*, *Kcnk3*, and *Scn5a*. These differences suggest that *Elektra* may have distinct functions in the nucleus and cytoplasm, modulating gene expression in a compartment-specific manner but, curiously, affecting in both cases the expression of ion channel genes.

Additionally, we tested whether *Qki* silencing could modulate *Elektra* and its downstream partners. *Qki* inhibition (Fig. 4F, Supplementary Fig. 5E-F) leads to downregulation of *Elektra* (Fig. 4G) as well as reduced levels of *Ryr2*, *Atp2a2*, *Casq2*, *Scn5a*, *Cacna1c, Cancb3*, *Canb4* and *Kcnn3*, while *Cacnb2* and *Ncx1* are upregulated (Fig. 4H). These data suggest the existence of a feedback mechanism between *Elektra* and Qki, which supports the hypothesis that *Elektra* regulates Qki expression. Therefore, our results propose that *Elektra* may act as an indirect regulator of ion channel gene expression by exerting post-transcriptional control through Qki.

**Fig. 5:**
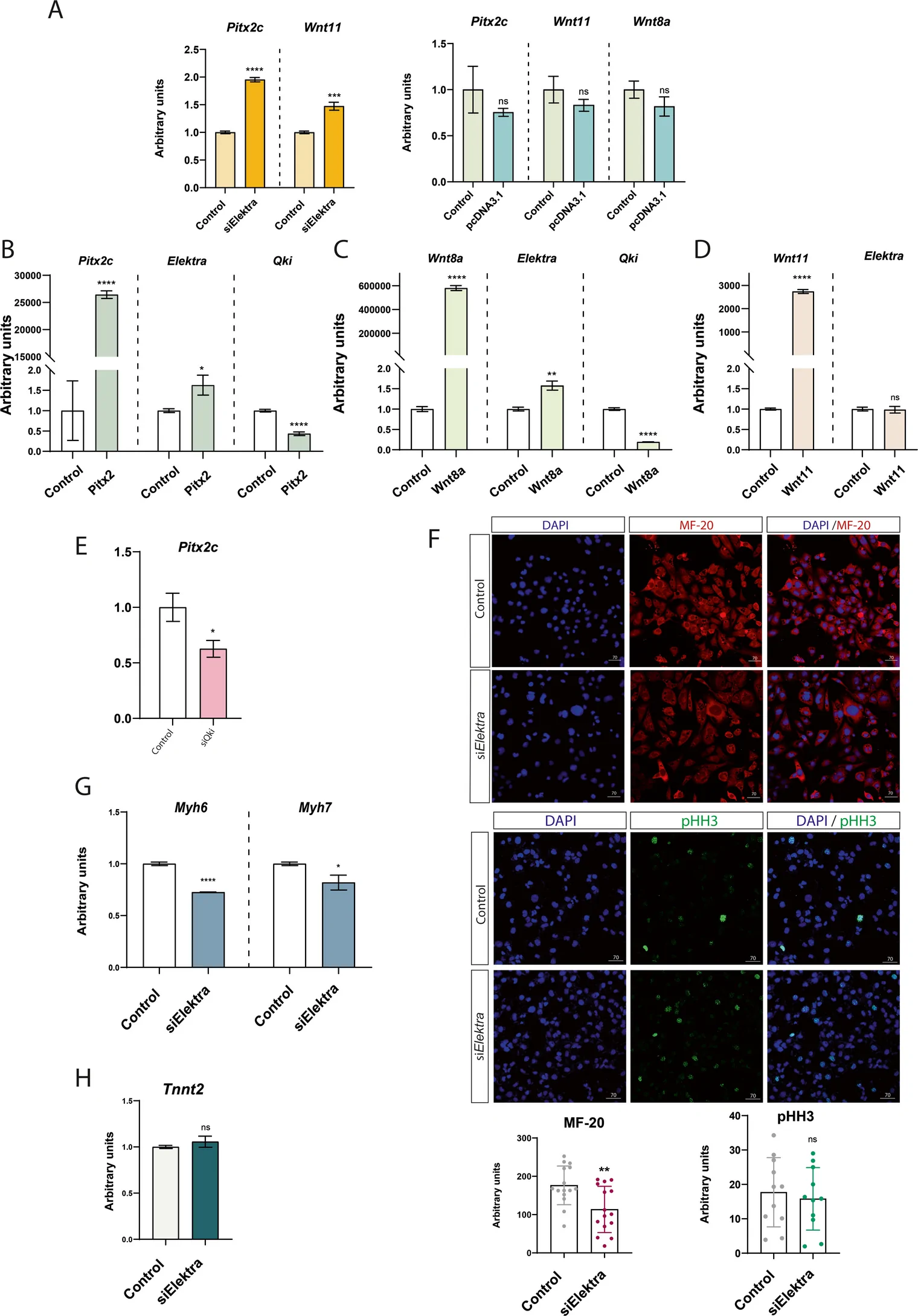
*Elektra* modulated *Pitx2c* and *Wnt* signaling, affecting cardiac ion channel and sarcomeric gene expression. Panel (**A**). RT-qPCR analyses of *Pitx2c*, *Wnt11* and *Wnt8a* expression in HL1 cardiomyocytes after silencing and overexpression of *Elektra* (n = 3). *Elektra* knockdown led to upregulation of *Pitx2c* and *Wnt11*, while *Elektra* overexpression did not modulate their expression. Panels (**B-D**). RT-qPCR analyses showing the effect of *Pitx2c* overexpression (B), *Wnt8a* overexpression (C), and *Wnt11* overexpression (D) on *Elektra* and *Qki* expression in HL1 cardiomyocytes (*n* = 3). *Pitx2c* and *Wnt8a* overexpression upregulated *Elektra* and downregulated *Qki*, whereas *Wnt11* overexpression did not affect *Elektra* levels. Panel (**E**). RT-qPCR analysis of *Pitx2c* expression following *Qki* knockdown in HL1 cells (*n* = 3), showing decreased *Pitx2c* levels. Panel (**F**). Representative images of immunofluorescence analysis of MF-20 and pHH3 protein expression in HL1 cardiomyocytes after *Elektra* inhibition (*n* = 3), showing reduced MF-20 signal compared to controls, whereas no significant changes were observed for pHH3. Panel (**G**). RT-qPCR analyses of *Myh6* and *Myh7* after *Elektra* inhibition (*n* = 3), which are significantly downregulated. Panel (**H**). RT-qPCR analysis of *Tnnt2* after *Elektra* inhibition (*n* = 3), with no significant differences. Statistical analysis: t-student (95% confidence interval); * *p*-value < 0.05; ** *p*-value < 0.005; *** *p*-value < 0.001; **** *p*-value < 0.0001

Given that pull-down and mass spectrometry analyses identified *Wnt* pathway proteins, such as *Wnt8a*, *Wnt8b*, and *Wnt11*, as potential interactors of *Elektra*, we investigated whether this lncRNA could influence *Wnt* signaling. In this context, it is important to highlight that we have previously reported the *Pitx2* > *Wnt* signaling is essential for maintaining cardiac action potential homeostasis [79]. For this reason, we subsequently analyzed the regulatory role of *Elektra* on *Pitx2c* and the *Wnt* signaling pathway. In this context, silencing *Elektra* led to upregulation of *Pitx2c* and *Wnt11*, whereas overexpression of *Elektra* had no effect on the mRNA expression of any of the genes (Fig. 5A). Furthermore, we investigated the role of *Elektra* inhibition on the expression of different Wnt signaling pathway components. Our analysis revealed a significant upregulation of canonical members as *Dkk3* and *Gsk3β*, while non-canonical members, including *Fzd6* remained unaltered (Supplementary Fig. 5C). Similarly, the loss-of-function of *Elektra* lead to increased expression of *Nr2f2* transcription factor, but no significant effects were identified in *Foxf1* or *Nr1f2* levels (Supplementary Fig. 5D). Taken together, these data demonstrate that the lncRNA *Elektra* can distinctly modulate the expression of *Qki*, *Wnt* signaling, and cardiac ion channels. Therefore, we explored the interlinks between these signaling pathways and *Elektra*. For that purpose, we analyzed *Elektra* and *Qki* expression after gain-of-function assays of *Pitx2c* and *Wnt* signaling, demonstrating that *Pitx2c* overexpression upregulated *Elektra* and downregulated *Qki* (Fig. 5B). Similarly, canonical *Wnt8a* overexpression resulted in *Elektra* upregulation and *Qki* downregulation (Fig. 5C), whereas non-canonical *Wnt11* does not alter *Elektra* expression (Fig. 5D). In addition, *Qki* knockdown led to a decrease in *Pitx2c* expression levels (Fig. 5E), suggesting a regulatory feedback loop involving *Qki*, *Pitx2c* and *Elektra*.

Since *Elektra* play a regulatory role in modulating cardiac ion channel expression and key signaling pathways such as *Qki* and *Pitx2c* > *Wnt*, both essential for proper electrical activity, we explored how this lncRNA could also modulate the contractile apparatus of cardiomyocytes. We therefore investigated myosin heavy chain protein expression after *Elektra* inhibition, which resulted in decreased MF20 immunoreactivity compared to controls (Fig. 5F), in the absence of differences in cell proliferation (Fig. 5F). Furthermore, qPCR analyses demonstrated that both sarcomeric myosin *Mhy6* and *Mhy7* transcripts were significantly downregulated after *Elektra* inhibition (Fig. 5G), in the absence of modulation of cardiac troponin T, *Tnnt2* (Fig. 5H). These results suggest that the inhibition of *Elektra* modulates the sarcomere function by regulating the expression of sarcomeric myosins, such as *Myh6* and *Myh7*. Therefore, *Elektra* may play also a significant role in regulating cardiac contraction in cardiomyocytes.

Overall, these data demonstrate that *Elektra* can modulate *Pitx2c* > *Wnt* signaling as well as Qki pathway impacting the expression of multiple genes coding for ion channels that are essential for the cardiac action potential configuration and, additionally, contributes to cardiomyocyte contractile function by controlling sarcomeric myosin expression.

#### *Alien* modulates *in vitro* expression of EMT-related genes and epicardial cell migration

Given that *Alien* displays differential expression between PE and EE, with predominant expression in MEC1 epicardial cells and primary nuclear localization, we performed loss-of-function assays using antisense oligonucleotides (ASOs) to investigate its potential role in epicardial development. ASOs were tested at two different concentrations (20 nM and 80 nM) and two post-transfection time points were analyzed (6h and 24 h). Previous PD and MS data identified several *Alien* -associated proteins involved in EMT, suggesting that this lncRNA may exert a regulatory role in this biological process.

**Fig. 6:**
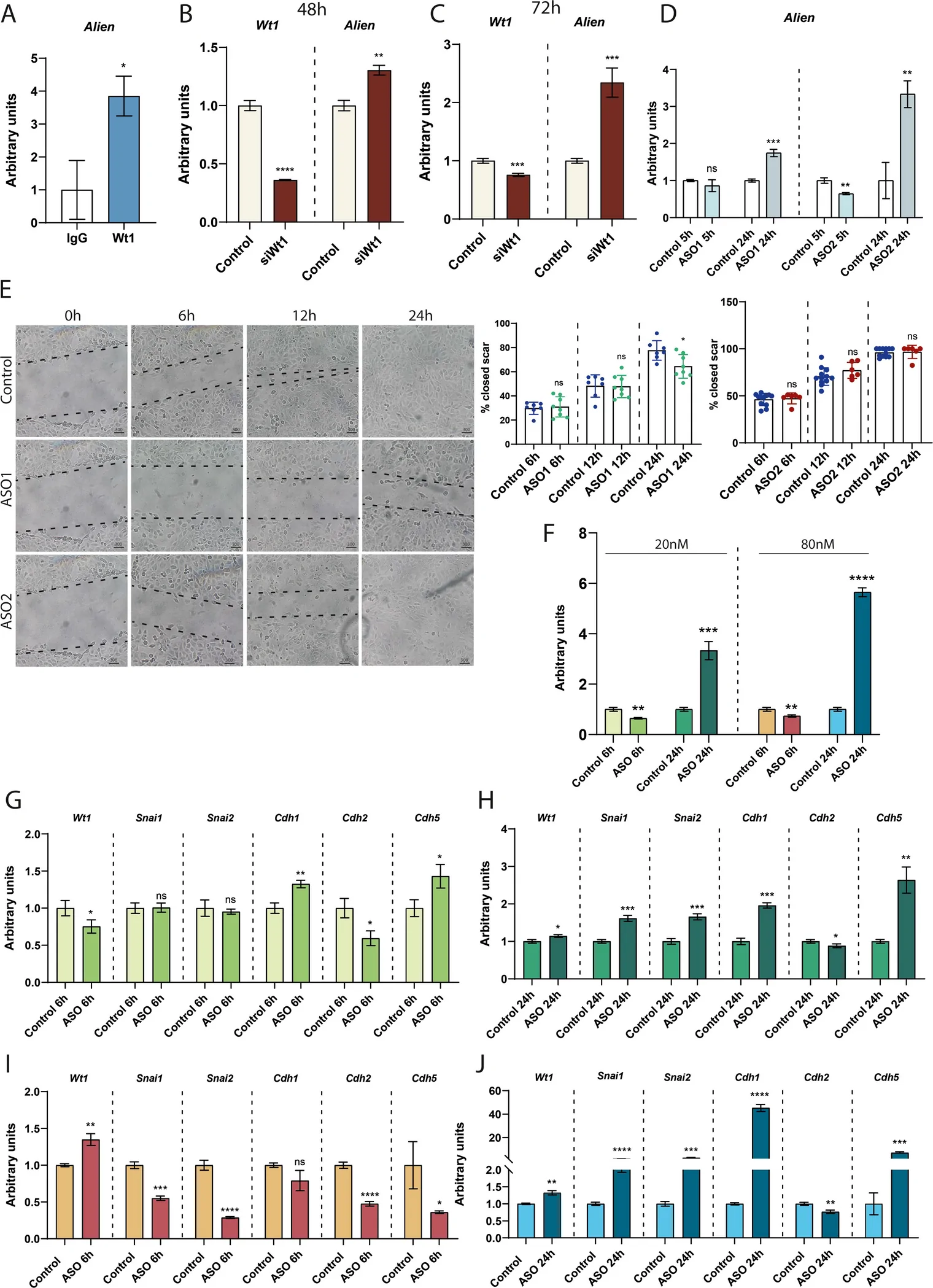
*Alien* modulates EMT-related gene expression and epicardial cell migration. Panel (**A**). RT-qPCR analysis of *Alien* enrichment following RNA immunoprecipitation (RIP) with Wt1-specific antibody versus IgG control in MEC1 epicardial cells. Panels (**B, C**). RT-qPCR analyses showing *Wt1* and *Alien* expression after *Wt1* knockdown with siRNA at 48 h (B) and 72 h (C) post-transfection (*n* = 3), demonstrating increased lncRNA levels following *Wt1* silencing. Panel (**D**). RT-qPCR analysis of *Alien* expression at 6 h and 24 h after transfection with ASO1 and ASO2 in MEC1 epicardial cells, showing overexpression with ASO1 and transient knockdown followed by overexpression with ASO2 (*n* = 3). Panel (**E**). Representative images of scratch assay of MEC1 epicardial cells at 6 h,12h and 24 h after transfection with ASO1 and ASO2. Note that ASO1 significantly reduces migration, while ASO2 shows no significant effect. Panel (**F**). RT-qPCR analysis of *Alien* levels at 6 h and 24 h following ASO2 treatment at 20nM and 80nM concentrations, confirming similar expression pattern in both conditions (*n* = 3). Panels (**G, H**). RT-qPCR analysis of EMT-related genes (*Wt1*, *Snai1*, *Snai2*, *Cdh1*, *Cdh2*, *Cdh5*) at 6 h and 24 h post-ASO2 *Alien* transfection at 20nM (*n* = 3). Panels (**I, J**). RT-qPCR analysis of EMT-related genes (*Wt1*, *Snai1*, *Snai2*, *Cdh1*, *Cdh2*, *Cdh5*) at 6 h and 24 h post-ASO2 *Alien* transfection at 80nM (*n* = 3). Statistical analysis: t-student (95% confidence interval); * *p*-value < 0.05; ** *p*-value < 0.005; *** *p*-value < 0.001; **** *p*-value < 0.0001

Among the proteins related to the EMT process, Wt1 stands out, and it has been identified as one of the proteins that directly interacts with *Alien* in MS assays (Supplementary Table 6). This interaction was validated using RNA immunoprecipitation (RIP), which revealed a significant enrichment of *Alien* in the Wt1-immunoprecipitated fraction compared to the IgG control (Fig. 6A). Furthermore, to analyze the functional relevance of this interaction, Wt1 loss-of-function assays were performed using siRNA, in which the inhibition of Wt1 at 48 h (Fig. 6B, Supplementary Fig. 6A, B) and 72 h (Fig. 6C) upregulated *Alien* expression (Fig. 6B, C). These results suggest that Wt1 may act as a negative regulator of *Alien*, controlling its expression through direct interaction.

To investigate the possible epicardial role of *Alien* through its interaction with Wt1, we also analyzed its impact on the migratory capacity of epicardial cells. To achieve this, we used two ASO molecules to generate different lncRNA expression profiles over time. Specifically, one of the ASOs (ASO1) treatment did not alter *Alien* expression at 6 h, but led to a significant upregulation at 24h. The second ASO (ASO2) caused a transient lncRNA knockdown at 6 h, followed by gain-of-function at 24 h (Fig. 6D). Scratch migration assays revealed a significant decrease in migratory capacity in ASO1-treated cells, whereas cells treated with ASO2 showed no relevant differences compared to controls (Fig. 6E). The reduced migratory capacity observed following ASO1 treatment is not related to cellular proliferation, as no significant differences in the number of pHH3^+^ cells were observed (Supplementary Fig. 6C, D). Additional Phalloidin staining revealed a decrease of F-actin polymerization in MEC1 epicardial cells after ASO treatment. Although morphological changes were no observed, the disruption of the actin cytoskeleton suggests that EMT is being triggered (Supplementary Fig. 6C, E). Overall, these results suggest that *Alien* overexpression could limit epicardial cell migration, while transient inhibition would not be sufficient to induce a significant functional change.

In addition, we analyzed the expression of several genes involved in the EMT process, such as *Snai1*, *Snai2* and different cadherins. We conducted loss-of-function assays of *Alien* using ASO2 at two concentrations (20 nM and 80 nM), evaluating their impact after 6 and 24h. A consistent pattern was observed: transient inhibition of *Alien* at 6 h, followed by lncRNA overexpression at 24 h (Fig. 6F). Due to these results, the functional effects at both times were analyzed. While the levels of inhibition and overexpression were comparable at both concentrations, the response of EMT-associated genes differed. After 6 h, the inhibition of *Alien* at 20 nM increased the expression of *Cdh1* and *Cdh5* while reducing the expression of *Wt1* and *Cdh2*, without affecting the levels of *Snai1* and *Snai2* (Fig. 6G). In contrast, *Alien* overexpression at 24 h significantly upregulated all the genes (Fig. 6H). These data suggest that overexpression of lncRNA could partially activates the EMT process without fully suppressing epithelial identity, as evidenced by the elevated increase in *Cdh1*. Overall, these results suggest that *Alien* may enhanced EMT-genes which promote an intermediate state of epithelial-to-mesenchymal transition in MEC1 epicardial cells.

On the other hand, inhibiting *Alien* for 6 h with an ASO2 at 80 nM increased the expression of *Wt1*, reduced the expression of *Snai1*, *Snai2*, *Cdh2* and *Cdh5*, without significantly altering *Cdh1* levels (Fig. 6I). However, after 24 h, when *Alien* overexpression was detected, *Wt1*, *Snai1*, *Snai2*, *Cdh1* and *Cdh5* levels remained elevated, with reduced levels observed in *Cdh2* (Fig. 6J). These findings suggest that *Alien* overexpression induces a mixed transcriptional profile that could correspond to an intermediate epithelial-to-mesenchymal state. Overall, these data support the notion that *Alien* exerts a modulatory role in EMT, whose expression levels regulate the balance between epithelial and mesenchymal phenotypes.

#### *Alien* modulates *ex vivo* EMT-associated gene expression and promotes epicardial cell EMT

**Fig. 7:**
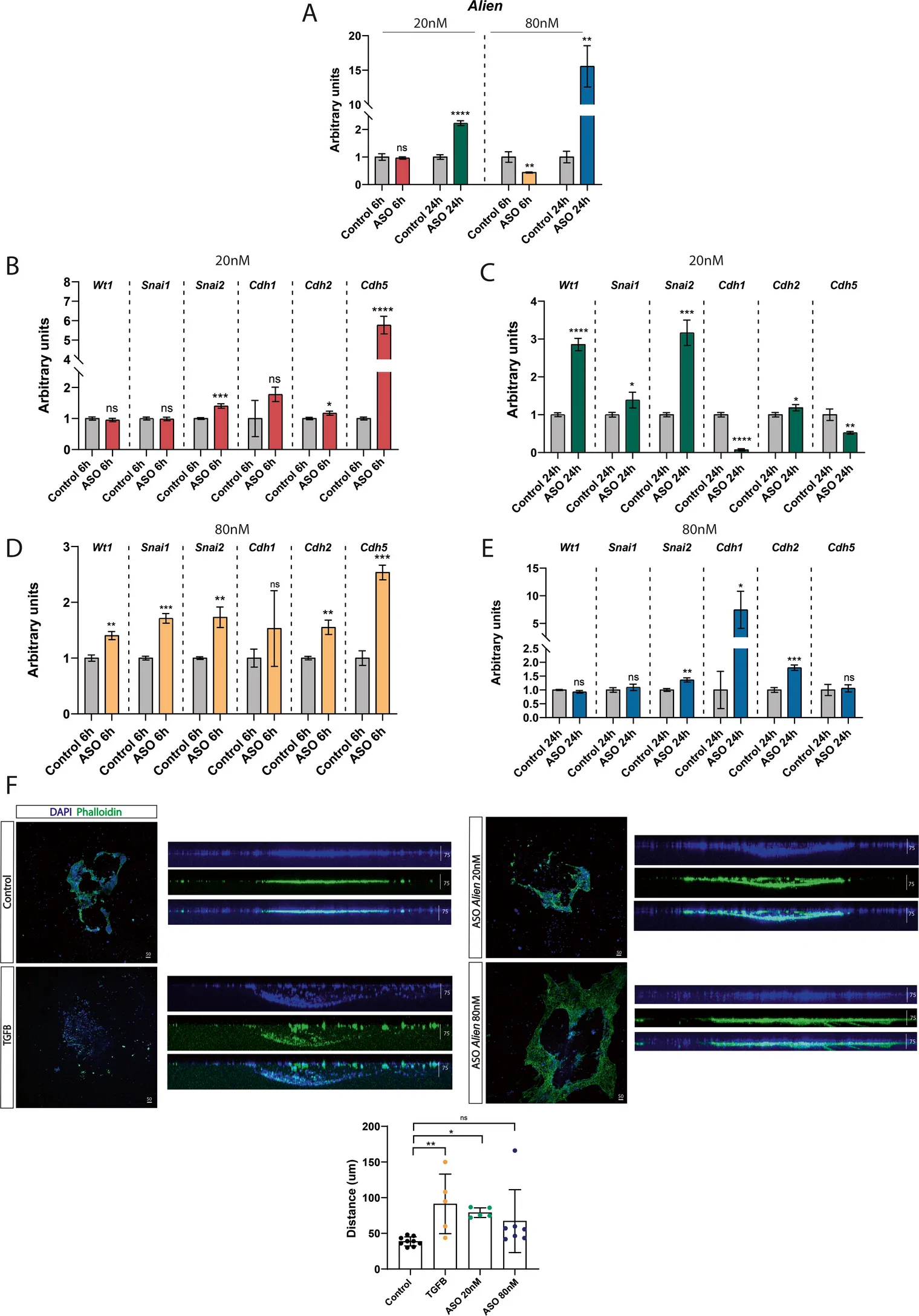
*Alien* regulates EMT-related gene expression and promotes mesenchymal transition in ex vivo epicardial cells. Panel (**A**). RT-qPCR analysis of *Alien* expression at 6 h and 24 h after ASO2 treatment at 20nM and 80nM in epicardial cells isolated from E10.5 mouse ventricular explants (each biological sample (*n*) corresponds to a pool of 7–10 explants). Note that overexpression is seen at 20nM and transient downregulation followed by overexpression is observed at 80nM. Panels (**B, C**). RT-qPCR analysis of EMT-related gene expression (*Wt1*, *Snai1*, *Snai2*, *Cdh1*, *Cdh2*, *Cdh5*) at 6 h and 24 h after 20nM ASO2 treatment (*n* = 3). At 24 h, *Alien* overexpression leads to upregulation of *Wt1*, *Snai1*, *Snai2* and *Cdh2*, with reduced *Cdh1* and *Cdh5* levels. Panels (**D, E**). RT-qPCR analysis of EMT-related genes (*Wt1*, *Snai1*, *Snai2*, *Cdh1*, *Cdh2*, *Cdh5*) at 6 h and 24 h after 80nM ASO2 treatment (*n* = 3). Panel (**F**). Representative images of collagen gel EMT assay performed ex vivo on epicardial cells from ventricular explants after TGF-β and ASO2 treatment at 20nM and 80nM. While explants do not initiate EMT without any treatment, transfection with TGF-β and ASO2 at 20nM induces EMT, in contrast to ASO2 at 80nM, for which no significant differences were observed (*n* = 5–8 explants per condition). Statistical analysis: Fig. 7A-E unpaired t-student test; Fig. 7 F, one-way ANOVA followed by Dunnett’s multiple-comparisons test. A 95% confidence interval was used for all analyses. * *p*-value < 0.05; ** *p*-value < 0.005; *** *p*-value < 0.001; **** *p*-value < 0.0001

Given the potential regulatory role of *Alien* on genes involved in the EMT and the epicardial cell migration, we also performed loss-of-function assays in *ex vivo* experiments using epicardial cells isolated from ventricular explants of E10.5 mouse embryos. Consistent with the *in vitro* results obtained in MEC1 epicardial cells, *ex vivo* treatment with 80 nM ASO2 led to a transient downregulation of *Alien* at 6 h, followed by a significant overexpression at 24 h (Fig. 7A). In contrast, treatment with a lower concentration (20 nM) did not alter *Alien* expression at 6 h but induced its overexpression at 24 h (Fig. 7A). Based on these expression dynamics, we assessed the mRNA levels of EMT-related genes (Fig. 7B-E).

Following 20 nM ASO2 treatment, a significant increase in *Snai2*, *Cdh2* and *Cdh5* mRNA levels was observed at 6 h, while no changes were detected in *Wt1*, *Snai1* or *Cdh1* expression (Fig. 7B). At 24 h, the overexpression of *Alien* resulted in downregulation of *Cdh1* and *Cdh5*, along with upregulation of *Wt1*, *Snai1*, *Snai2* and *Cdh2* (Fig. 7C). Altogether, these results indicate that the overexpression of *Alien* promotes the upregulation of several genes associated with the EMT program, including *Wt1*, *Snai1*, *Snai2* and *Cdh2*, while reducing epithelial markers such as *Cdh1*. These findings suggest that this lncRNA may play a role in promoting a mesenchymal transcriptional profile in epicardial cells. In contrast, an initial knockdown of *Alien* using a higher ASO2 concentration (80 nM) did not produce the same effect in epicardial cells. The loss-of-function of the lncRNA at 6 h led to an increase in the expression of *Wt1*, *Snai1*, *Snai2*, *Cdh2* and *Cdh5*, without affecting *Cdh1* levels (Fig. 7D). However, at 24 h, when a significant *Alien* overexpression was observed, only *Snai2*, *Cdh2* and *Cdh1* were upregulated, with *Cdh1* showing the most consistent increase. On the other hand, no significant changes were detected in the expression of *Wt1*, *Snai1* or *Cdh5* (Fig. 7E). This pattern suggests that *Alien* knockdown may partially promote the expression of EMT-related genes, although with a more limited effect. Altogether, these findings suggest that *Alien* plays a regulatory role in EMT, with prolonged expression promoting the upregulation of EMT-related genes, including *Wt1*, *Snai1* and *Snai2*, and the downregulation of *Cdh1*.

In order to confirm the transcriptional EMT modulation by *Alien*, we performed *ex vivo* functional EMT assays using collagen gels. Although epicardial cells isolated from explants did not spontaneously initiate the EMT process, we confirmed their capacity to undergo EMT upon treatment with TGF-β, a key inducer of epithelial-to-mesenchymal transition. This EMT-inducing ability was also observed in epicardial cells treated with 20 nM ASO2, whereas no significant changes were detected using the higher concentration (80 nM) (Fig. 7F). These data support the mRNA results, suggesting that sustained overexpression of *Alien* promotes the EMT in epicardial cells.

## Discussion

Long non-coding RNAs are increasingly recognized as important regulators of gene expression in different biological processes [44, 80]. However, their functional roles during embryonic development remain largely uncharacterized. In previous studies, we identified two lncRNAs, *Elektra* and *Alien*, with differential expression between the PE and EE [58]. These lncRNAs may play crucial roles in epicardial development since their expression in embryonic and adult tissues suggests diverse functions depending on the developmental stage or tissue context [81, 82]. Although both lncRNAs are expressed at low levels in the heart, we focused on this organ due to its developmental relevance. qPCR and SCRINSHOT assays demonstrated their presence in cardiomyocytes, endocardial and epicardial cells, with *Elektra* enriched in MEVEC endocardial cells and *Alien* in MEC1 epicardial cells. *Elektra* shows cell-type-specific subcellular distribution, since it was mainly cytoplasmic in MEVEC, but nuclear and cytoplasmic in the other cell types. These data suggest that *Elektra* may regulate transcriptional processes in the nucleus, while it acts as a post-transcriptional modulator in the cytoplasm, similar to other lncRNAs such as *HBL1* or *H19* [83, 84]. In the cytoplasm *HBL1* sponges *miR-1*, regulating cardiogenic differentiation, while *H19* modulates cardiomyocyte apoptosis via *miR-675* [83, 84]. However, in the nucleus, both lncRNAs participate in epigenetic regulation through the PRC2 complex [57, 85, 86]. In contrast, our data show that *Alien* displays a predominantly nuclear localization in the cardiac cell types analyzed, indicating its nuclear function. Interestingly, previous studies reported that this lncRNA exhibits nuclear, perinuclear and cytoplasmic localization in vascular progenitors, while in liver cells its expression is mainly nuclear [87], indicating that its subcellular distribution and function may depend on the developmental stage or the cell type context [51].

Several studies highlight the importance of transcription factors in modulating lncRNA expression [52, 68, 88]. We investigated the regulatory effects of cardiogenic transcriptional factors, such as *Pitx2c* [89, 90], *Mef2c* [73, 91], *Srf* [92, 93] and *Nkx2.5* [94, 95], on *Elektra* and *Alien* expression through loss- and gain-of-function experiments. Our data indicate cell-type-specific transcriptional regulation, since all transcription factors modulate *Elektra* expression in epicardial cells, whereas *Pitx2c* overexpression controls *Elektra* in myocardial cells. Similarly, *Pitx2c* regulates *Alien* in EPIC epicardial cells, while *Srf* and *Mef2c* exert stronger control in MEC1 epicardial cells. These findings demonstrate the context-dependent regulation of lncRNAs by transcriptional factors within cardiac cell types [37, 96, 97].

To explore the biological function of *Elektra* and *Alien*, we performed PD assay followed by MS to identify specific proteins directly interacting with each lncRNA. For *Elektra*, assays were conducted in the three cardiac cell types, and, surprisingly, no common shared interacting proteins were found among them, highlighting the functional specificity of this lncRNA in different cellular contexts. In MEC1 epicardial cells and MEVEC endocardial cells, *Elektra* interacted with proteins from both nuclear and cytoplasmic compartments, consistent with its localization. These findings support a dual role for *Elektra* in both transcriptional and post-transcriptional regulation, as previously reported for other cardiac lncRNAs such as *MALAT1*, which regulates gene expression in the nucleus by controlling nuclear speckle localization and splicing factors, while acts in the cytoplasm as a miRNA sponge for *miR-155* and *miR-125*, modulating different cardiac processes [98–101]. In HL1 myocardial cells, most interacting proteins were cytoplasmic, indicating a more relevant post-transcriptional role of *Elektra* in this context, in line with other lncRNAs, such as *ZFAS1*, that acts in the cytoplasm by binding to *Atp2a2* to regulate its expression and therefore modulate cardiomyocyte calcium homeostasis [102, 103].

Gene Ontology (GO) analysis of the *Elektra* interactome in cardiomyocytes revealed enrichment in cytoplasmic processes such as RNA splicing regulation. Notably, one of the interacting proteins was the KH-domain RNA-binding protein Qki, a well-established regulator of alternative splicing in cardiomyocytes [78]. Functional assays showed that *Elektra* negatively regulates *Qki*, with its knockdown increasing *Qki* levels and altering ion channel gene expression. These effects were mainly associated with the cytoplasmic fraction of *Elektra*, suggesting a post-transcriptional regulation. In contrast, nuclear *Elektra* showed a distinct regulatory pattern, with only *Ncx1* consistently affected in both compartments, emphasizing the importance of subcellular localization in lncRNA function [104, 105]. Previous studies have shown that Qki regulates the splicing of genes related to cardiac contraction and electrophysiology, including *Ryr2* and *Cacnb1* [78, 106]. Our findings suggest that *Elektra* modulates these pathways by regulating *Qki* levels, without affecting the Qki-driven alternative splicing of these ion channels (data not shown). Moreover, *Qki* inhibition reduced *Elektra* expression, in addition to altering the levels of several calcium, sodium and potassium channel genes, in line with previous data [78, 106]. These results suggest a regulatory feedback for maintaining proper splicing and ion channel expression in cardiomyocytes.

Additionally, *Elektra* interacts with proteins of the *Wnt* signaling pathway such as *Wnt8a*, *Wnt8b* and *Wnt11*, a pathway involved in cardiac electrophysiology and regulated by *Pitx2c* [30, 79, 107]. *Pitx2c* upregulates *Elektra*, in line with other lncRNAs previously investigated in our laboratory, such as *Walrad*, *Walras*, and *Walce*, that also participate in this pathway, suggesting a complex lncRNA network [65]. Furthermore, we found a cross-talk between *Qki* and *Pitx2c* > *Wnt* pathway, with reciprocal regulation. Lastly, *Elektra* also modulates sarcomeric gene expression essential for cardiac structure and contractile function and liked to atrial fibrillation (AF) [108–110]. Therefore, *Elektra* may play a key role in regulating both ion channel and sarcomeric gene expression through *Qki* and *Pitx2c* > *Wnt* signaling, and its dysregulation could contribute to arrhythmogenic cardiac pathologies such as AF.

In MEC1 epicardial cells, *Alien* interacts with both nuclear and cytoplasmic proteins, supporting the notion that lncRNA function can vary depending on the cellular context [111]. GO analysis revealed a strong enrichment in EMT, and notably identified *Wt1* among the interacting proteins. *Wt1* is a well-known transcription factor essential for epicardial EMT [14, 112], and our data showed that *Wt1* represses *Alien*, suggesting its involvement in EMT regulation. Functional assays showed that *Alien* overexpression impaired epicardial cell migration, while its inhibition had no apparent effect. For this lncRNA, functions have been described in other biological contexts, such as the liver, where *in vitro* studies have shown that it regulates the expression of *Foxa2* transcription factor, thereby modulating the progression of liver fibrosis [87]. *Alien* -Foxa2 interaction has also been described in the lung, where it forms a regulatory feedback loop that maintains epithelial barrier integrity and suppresses cell migration [113]. Numerous studies in other biological systems have demonstrated that lncRNAs can modulate cell migration and EMT through diverse mechanisms, for example, *MEG3*, *lncRNA-HIT*, and *lncTCF7* in different cancer types [114–116], or *lncRNA-ATB* and *MALAT1* in pulmonary fibrosis-associated EMT [117, 118]. However, in the cardiac context, our understanding of these processes remains limited, highlighting the need to further research to identify new lncRNAs with critical functions in epicardial EMT.

To further understand this behavior, we analyzed the expression of key EMT markers following ASO treatment, and we observed dose-dependent effects on gene expression, highlighting the significant impact of this lncRNA on EMT regulation. Such variability is consistent with studies showing that ASO activity is not only sequence-specific but also dependent on dose and tissue distribution, which can critically influence knockdown efficiency and downstream effects [119, 120]. Overall, our data support a role for *Alien* in EMT regulation, mediated through transcription factors and adhesion molecules in epicardial cells. To confirm these findings, we performed *ex vivo* EMT essays, in which collagen gel experiments demonstrated that sustained overexpression of *Alien* promotes EMT in epicardial cells, a finding consistent with qPCR data showing upregulation of EMT markers such as *Wt1*, *Snai1* and *Snai2*, and downregulation of the epicardial marker *Cdh1*. Collectively, these data support the role of *Alien* as a regulatory factor in the control of epicardial EMT, suggesting that its participation is essential during embryonic epicardial development.

**Fig. 8:**
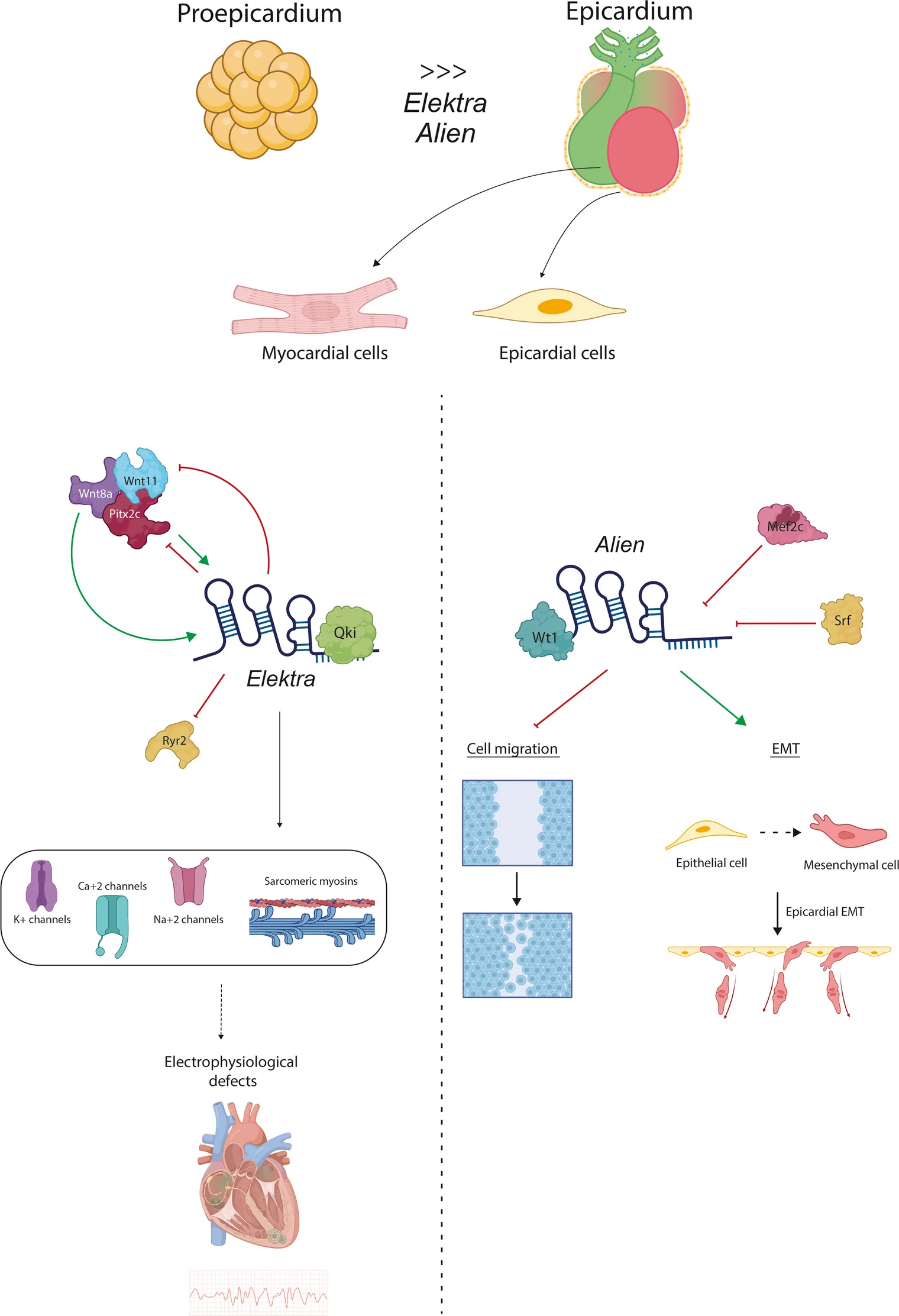
Proposed working model of *Elektra* and *Alien* function during cardiac development. Schematic representation summarizing the functional roles of *Elektra* and *Alien* in distinct cardiac lineages. In myocardial cells, *Elektra* interacts with Qki and modulates *Pitx2c* > *Wnt* signaling, thereby regulating the expression of ion channels and sarcomeric genes, contributing to electrical and contractile homeostasis. In epicardial cells, *Alien* interacts with Wt1 and regulates EMT-associated genes, influencing cell migration and promoting a partial epithelial-mesenchymal transition. Schemes were created using Biorender (https://www.biorender.com/)

In summary, our results provide evidence that both lncRNAs, *Elektra* and *Alien*, are widely expressed across multiple embryonic and adult tissues, exhibiting cell-type-specific subcellular localization and transcriptional regulation. Within the myocardial context, *Elektra* potentially regulates the expression of ion channel and sarcomeric contraction genes. Our findings point out a mechanism involving Qki binding and the *Pitx2c* > *Wnt* pathway, providing a possible molecular role in cardiac function (Fig. 8). In contrast, *Alien* directly interacts with Wt1 and regulates pivotal genes involved in the cell migration and EMT process (Fig. 8). These results provide mechanistic insights into how Alien could potentially influence epicardial behaviour during epicardial embryonic development.

While this study provides a data-rich landscape of lncRNA-protein interaction in the cardiac context, several limitations must be acknowledged. First, the current conclusions are primarily based on *in vitro* and *ex vivo* models, and their physiological relevance remains to be fully confirmed through further *in vivo* studies. Furthermore, although we identify key regulatory networks, the direct physical interaction between the candidate lncRNAs and their target proteins has yet to be definitively proven. Future research employing high-resolution techniques, such as eCLIP, will be essential to exclude "piggy-back" effects and confirm covalent binding. Finally, the lack of functional assays of action potentials (e.g., patch-clamp) means that the actual impact of these molecular changes on cellular excitability is not yet fully characterized. Acknowledging these gaps is crucial for a balanced interpretation and highlights the need for functional and systemic validation in future *in vivo* analysis.

## Conclusions

This study suggests that *Elektra* and *Alien* are key lncRNAs with different and specific functions in the developing heart. Our finding indicate that *Elektra* likely acts in myocardial cells by interacting with Qki and controlling pathways related to electrical activity and contractility, while *Alien* potentially modulates epicardial cell migration and EMT through its interaction with Wt1. These findings highlight the potential importance of lncRNAs as context-dependent regulators of cardiac development and support their potential involvement in tissue-specific processes.

## Supplementary Information

Below is the link to the electronic supplementary material.

Supplementary file1 (PDF 21975 KB)

Supplementary file2 (PDF 78088 KB)

Supplementary file3 (PDF 494 KB)

Supplementary file4 (PDF 1088 KB)

Supplementary file5 (PDF 4505 KB)

Supplementary file6 (PDF 13845 KB)

Supplementary file7 (PDF 59 KB)

Supplementary file8 (PDF 217 KB)

Supplementary file9 (PDF 169 KB)

Supplementary file10 (PDF 124 KB)

Supplementary file11 (PDF 246 KB)

Supplementary file12 (PDF 993 KB)

## Data Availability

RNAseq data were uploaded into Gene Expresssion Onmibus platform with accession number GSE189344 [53]. https://www.ncbi.nlm.nih.gov/geo/query/acc.cgi?acc=GSE189344

## Abbreviations

AF: Atrial fibrillation
ASO: Antisense oligonucleotide
BP: Biological process
CC: Cellular component
cDNA: Complementary DNA
EE: Embryonic epicardium
EMT: Epithelial to mensenchymal transition
EPDCs: Epicardial derived cells
GO: Gene ontology
HF: Heart failure
lncRNAs: Long non-coding RNAs
MF: Molecular function
miRNAs: MicroRNAs
mRNAs: Messeger RNAs
MS: Mass spectrometry
ncRNAs: Non-coding RNAs
P/S: Penicillim/streptromycin
PBS: Phosphate buffer saline
PD: Pull-down
PE: Proepicardium
PFA: Paraformaldehyde
RBPs: Ribonucleoproteins
RIP: RNA immunoprecipitation
rRNAs: Ribosomal RNAs
RT-qPCR: Reverse transcriptase-quantitative polymerase chain reaction
SCRINSHOT: Single cell resolution in situ hybridization on tissues
siRNA: Small interfering RNA
snoRNAs: Small nucleolar RNAs
snRNAs: Small nuclear RNAs
tRNAs: Transfer RNAs
WB: Western blot
