# Supplementary material for: Elektra-Qki and Alien-Wt1 lncRNA-protein interaction controls myocardial ion channel expression and epicardial EMT during heart development in mice
Source: Cell Mol Life Sci. 2026 Jul 7;83(1):356. doi: 10.1007/s00018-026-06322-x (PMC13631190; doi:10.1007/s00018-026-06322-x)
Supplement: Supplementary file 1 [file 18_2026_6322_MOESM10_ESM.pdf]

Uniprot ID	Gene	Short name	Subcellular location	Gene ontology
Q9Z331	Krt6b	Keratin, type II cytoskeletal 6B	-	Intermediate filament organization, keratinization, morphogenesis of an epithelium
Q6IFZ9	Krt74	Keratin, type II cytoskeletal 74	-	keratin filament
Q8CBY1	Samd4a	Protein Smaug homolog 1	Cytoplasm, dendrite	RNA binding, translation repressor activity, positive regulation of translation, regulation of mRNA stability
Q99NB9	Sf3b1	Splicing factor 3B subunit 1	Nucleus	B-WICH complex, chromatin, nucleolus, chromatin remodeling, mRNA splicing via spliceosome
P62267	Rps23	Small ribosomal subunit protein uS12	Nucleus, cytoplasm, endoplasmic reticulum	Cytoplasmic translation, maintenance of translational fidelity, ribosomal small subunit biogenesis, stress granule assembly, translation
Q921M3	Sf3b3	Splicing factor 3B subunit 3	Nucleus	mRNA splicing via spliceosome, nucleic acid binding, positive regulation of DNA-templated transcription, regulation of DNA repair, regulation of RNA splicing
Q05CL8	Larp7	La-related protein 7	Nucleus, nucleoplasm	7SK snRNA binding, U6 snRNA binding, mRNA processing, RNA splicing, regulation of mRNA splicing via spliceosome
P47962	Rpl5	Large ribosomal subunit protein uL18	Nucleus, nucleolus, cytoplasm	5S rRNA binding, structural constituent of ribosome, cytoplasmic translation, translation at postsynapse, translation at presynapse
Q9CPR4	Rpl17	Large ribosomal subunit protein uL22	Cytoplasm	Large ribosomal subunit rRNA binding, structural constituent of ribosome, cytoplasmic translation, translation at postsynapse, translation at presynapse
Q6DIC0	Smarca2	SWI/SNF-related matrix-associated actin-dependent regulator of chromatin subfamily A member 2	Nucleus	Chromatin organization, chromatin remodeling, nucleosome assembly, regulation of transcription by RNA polymerase II, regulation of G1/S transition of mitotic cell cycle
Q64282	Ifit1	Antiviral innate immune response effector IFIT1	Cytoplasm	RNA binding, RNA sequestering activity, defense response to virus, innate immune response, response to interferon-alpha
Q9CQV8	Ywhab	14-3-3 protein beta/alpha	Cytoplasm	Histone deacetylase binding, protein kinase inhibitor activity, protein phosphatase inhibitor activity, negative regulation of DNA-templated transcription, positive regulation of transcription by RNA polymerase II
P15864	H1-2	Histone H1.2	Nucleus	Extracellular matrix structural constituent, hematopoietic progenitor cell differentiation
Q30D77	Col24a1	Collagen alpha-1	Extracellular matrix	Extracellular matrix structural constituent, hematopoietic progenitor cell differentiation
P35979	Rpl12	Large ribosomal subunit protein uL11	Cytoplasm	RNA binding, structural constituent of ribosome, cytoplasmic translation, translation at postsynapse, translation at presynapse.

Uniprot ID	Gene	Short name	Subcellular location	Gene ontology
A2AM05	Cntln	Centlein	Cytoplasm, cytoskeleton, microtubule organizing center, centrosome, centriole	Protein domain specific binding, protein kinase binding, centriole-centriole cohesion, protein-containing complex assembly, spermatogenesis.
Q8CHG3	Gcc2	GRIP and coiled-coil domain-containing protein 2	Cytoplasm, Golgi apparatus	Identical protein binding, Golgi ribbon formation, microtubule anchoring, protein localization to Golgi apparatus, regulation of protein exit from endoplasmic reticulum.
P16627	Hspa1l	Heat shock 70 kDa protein 1-like	-	ATP-dependent protein folding chaperone, heat shock protein binding, protein refolding, cell differentiation, spermatogenesis.
Q9QYX7	Pclo	Protein piccolo	Presynaptic active zone	Calcium ion binding, cytoskeleton organization, insulin secretion, regulation of exocytosis, synaptic vesicle clustering.
P63276	Rps17	Small ribosomal subunit protein eS17	Nucleus, nucleolus, cytoplasm	Structural constituent of ribosome, cytoplasmic translation, ribosomal small subunit biogenesis, translation, synaptic function.
P15508	Sptb	Spectrin beta chain, erythrocytic	Cytoplasm, cytoskeleton	Actin binding, structural constituent of cytoskeleton, plasma membrane organization, actin filament capping, hemopoiesis.
Q6ZQ06	Cep162	Centrosomal protein of 162 kDa	Cytoplasm, cytoskeleton, microtubule organizing center, centrosome, centriole	Cilium assembly, centriole function, spindle organization, Golgi apparatus involvement, nucleoplasm localization.
P49586	Pcyt1a	Choline-phosphate cytidylyltransferase A	Cytoplasm, cytosol	Calmodulin binding, choline-phosphate cytidylyltransferase activity, molecular function inhibitor activity, phosphatidylcholine binding, phosphatidylcholine biosynthetic process.
Q9JIK5	Ddx21	Nucleolar RNA helicase 2	Nucleus, nucleolus, cytoplasm	ATP binding, RNA binding, chromatin remodeling, defense response to virus, positive regulation of transcription by RNA polymerase II.
Q80XS6	Samd4b	Protein Smaug homolog 2	Nucleus, cytoplasm	mRNA binding, translation repressor activity, cerebellar neuron development, regulation of mRNA stability.
P62889	Rpl30	Large ribosomal subunit protein eL30	Cytoplasm	RNA binding, structural constituent of ribosome, cytoplasmic translation, antimicrobial humoral immune response mediated by antimicrobial peptide, defense response to Gram-negative bacterium.
Q9WTM5	Ruvbl2	RuvB-like 2	Nucleus, nucleolus, cytoplasm	Centrosome, ciliary basal body, ATP binding, chromatin remodeling, regulation of DNA repair.
Q3URD3	Slmap	Sarcolemmal membrane-associated protein	Cell membrane, sarcolemm	Protein-macromolecule adaptor activity, negative regulation of Hippo signaling, centrosome function, membrane-related processes, structural components of muscle
