# Supplementary material for: Elektra-Qki and Alien-Wt1 lncRNA-protein interaction controls myocardial ion channel expression and epicardial EMT during heart development in mice
Source: Cell Mol Life Sci. 2026 Jul 7;83(1):356. doi: 10.1007/s00018-026-06322-x (PMC13631190; doi:10.1007/s00018-026-06322-x)
Supplement: Supplementary file 2 [file 18_2026_6322_MOESM11_ESM.pdf]

Uniprot ID	Gene	Short name	Subcellular location	Gene ontology
P01740	-	T-cell receptor gamma chain V region V108A	-	Adaptive immune response
Q3THG9	Aarsd1	Alanyl-tRNA editing protein Aarsd1	Cytoplasm	Alanyl-tRNA aminoacylation, regulation of translational fidelity
Q9DBM0	Abcg8	ATP-binding cassette sub-family G member 8	Plasma membrane	Cholesterol efflux, response to muscle activity, triglyceride homeostasis
Q923W9	Adam33	Disintegrin and metalloproteinase domain-containing protein 33	Plasma membrane	Proteolysis, regulation of cell population proliferation, regulation of citokine production
Q8BM96	Adgrg7	Adhesion G-protein coupled receptor G7	Plasma membrane	Cell surface receptor signaling pathway
O35423	Agxt	Alanine--glyoxylate aminotransferase	Mitochondrial matrix, peroxisome	Glycine biosynthetic process, Notch signaling pathway, response to cAMP
Q3TY86	Aifm3	Apoptosis-inducing factor 3	Cytosol, endoplasmic reticulum, mitochondrion	Execution phase of apoptosis
Q91WR5	Akr1c21	Aldo-keto reductase family 1 member C21	Cytoplasm	Steroid biosynthetic process
P55249	Alox12e	Polyunsaturated fatty acid (12S)/(13S)-lipoxygenase	Cytoplasm	Arachidonic acid metabolic process, lipid oxidation, lipoxygenase pathway
Q8K1C0	Angel2	Protein angel homolog 2	Cytoplasm	3'-UTR-mediated mRNA stabilization, mitochondrial mRNA 3'-end processing, negative regulation of mitotic cell cycle
Q8C5H1	Ano4	Anoctamin-4	Plasma membrane	Calcium activated galactosylceramide scrambling, chloride transmembrane transport, establishment of localization in cell
Q8CJ69	Bmper Cv2	BMP-binding endothelial regulator protein	Extracellular region	Blood vessel endothelial cell proliferation involved in sprouting angiogenesis, BMP signaling pathway, SMAD protein signal transduction
Q810B3	Bsx	Brain-specific homeobox protein homolog	Nucleus, cytoplasm	Brain development, eating and locomotory behavior, mammary gland involution, positive regulation of transcription by RNA polymerase II
Q3UZ09	C1rl	Complement C1r subcomponent-like protein	Extracellular region	Innate immune response, proteolysis
Q8R0S4	Cacnb4	Voltage-dependent L-type calcium channel subunit beta-4	Nucleus, cytoplasm, voltage-gated calcium channel complex	calcium ion transport, muscle cell development, regulation of synaptic vesicle exocytosis, regulation of voltage-gated calcium channel activity, T cell receptor signaling pathway
Q8VEC4	Calhm2	Calcium homeostasis modulator protein 2	Plasma membrane	ATP export, calcium ion import, monoatomic ion transmembrane transport, positive regulation of apoptotic process
Q8BUG5	Caps2	Calcyphosin-2	Nucleus, cytoplasm	Calcium ion binding

Uniprot ID	Gene	Short name	Subcellular location	Gene ontology
P51637	Cav3	Caveolin-3	Caveola, endoplasmic reticulum,Golgi membrane, plasma membrane, sarcolemma	Actin filament organization, calcium ion transport, cardiac muscle cell development, caveola assembly, ventricular cardiac muscle cell action potential
**Q9CR29**	Ccdc43	Coiled-coil domain-containing protein 43	Cytosol	-
**D3Z3K2**	Ccnb1ip1	E3 ubiquitin-protein ligase CCNB1IP1	Nucleus, cytosol	Blastocyst formation, protein ubiquitination, condensed nuclear chromosome
**Q52KE7**	Ccnl1	Cyclin-L1	Nucleus, cytosol, transcription elongation factor complex	Regulation of apoptotic process, regulation of cell cycle, regulation of centrosome cycle, regulation of RNA splicing, regulation of transcription by RNA polymerase II [
**Q8BHL7**	Cdc42se1	CDC42 small effector protein 1	Cytoskeleton, plasma membrane	Negative regulation of JNK cascade, phagocytosis, regulation of cell shape, regulation of Rho protein signal transduction
**Q8BI72**	Cdkn2aip	CDKN2A-interacting protein	Nucleus	DNA damage response, regulation of cell growth, protein stability
**Q921W0**	Chmp1a	Charged multivesicular body protein 1a	Nucleus, cytoplasm, endosome, lysosome, plasma membrane, extracelullar space	Autophagy, mitotic chromosome condensation, multivesicular body assembly, nucleus organization, protein transport
**Q3TZ65**	Cimap1d	Protein CIMAP1D	Cytoplasmic microtubule	-
**Q99LI2**	Clcc1	Chloride channel CLIC-like protein 1	Endoplasmic reticulum, mitochondrion	Endoplasmic reticulum calcium ion homeostasis
**O88587**	Comt	Catechol O-methyltransferase	Cytosol, mitochondrion, plasma membrane,	Artery development, dopamine secretion, response to hypoxia, multicellular organism growth, cerebellar cortex morphogenesis
**Q8BT60**	Cpne3	Copine-3	Nucleus, cytoplasm, mitochondrion, plasma membrane	Cellular response to calcium ion, cellular response to growth factor stimulus, positive regulation of cell migration
**Q9JHS9**	Cwc15	Spliceosome-associated protein CWC15 homolog	Nucleus, mitochondrion	mRNA splicing, via spliceosome
**Q8CI33**	Cwf19l1	CWF19-like protein 1	-	-
**Q9DBN9**	Ddx59	Probable ATP-dependent RNA helicase DDX59	Nucleus, cytoplasm	ATP, RNA binding, RNA helicase activity
**Q921R4**	Dnajc14	DnaJ homolog subfamily C member 14	Endoplasmic reticulum membrane	Protein transport
**Q99LN9**	Dohh	Deoxyhypusine hydroxylase	-	Peptidyl-lysine modification to peptidyl-hypusine
**Q9CWH0**	Dppa2	Developmental pluripotency-associated protein 2	Nucleoplasm, nucleus	Epigenetic regulation of gene expression, positive regulation of stem cell proliferation, stem cell population maintenance
**Q99N11**	Dusp22	Dual specificity protein phosphatase 22	Cytoplasm, plasma membrane	Cellular response to epidermal growth factor stimulus, immune system process, negative regulation of cell migration

Uniprot ID	Gene	Short name	Subcellular location	Gene ontology
Q61501	E2f1	Transcription factor E2F1 (E2F-1)	Nucleus, cytoplasm	Cellular response to xenobiotic stimulus, DNA-templated transcription, ranscription by RNA polymerase II, positive regulation of apoptotic process, positive regulation of fibroblast proliferation
Q9QZH6	Ecsit	Evolutionarily conserved signaling intermediate in Toll pathway	Nucleus, cytoplasm, mitochondrion	BMP signaling pathway, innate immune response, mesoderm formation, regulation of transcription by RNA polymerase II, regulation of protein complex stability
P52800	Efnb2	Ephrin-B2	Plasma membrane, adherens junction	Adherens junction organization, animal organ morphogenesis, blood vessel morphogenesis, cell adhesion, cell differentiation, cell migration involved in sprouting angiogenesis, nervous system development, positive regulation of cardiac muscle cell differentiation
Q80VP1	Epn1	Epsin-1	Nucleus, cytoplasm, plasma membrane	Embryonic organ development, endocytosis, Notch signaling pathway
Q3UNU4	Erich4	Glutamate-rich protein 4	-	-
Q6KAR6	Exoc3	Exocyst complex component 3	Golgi apparatus, cytoplasm	Membrane fission, mitotic cytokinesis, protein transport, exocytosis
Q8K097	Faim2	Protein lifeguard 2	Endoplasmic reticulum, Golgi apparatus, plasma membrane	Apoptotic signaling pathway, cerebellum development, response to ischemia
P97805	Fam3d	Protein FAM3D	Extracellular space	Negative regulation of insulin secretion
Q9QXW0	Fbxl6	F-box/LRR-repeat protein 6	-	SCF ubiquitin ligase complex
Q8CHQ0	Fbxo4	F-box only protein 4	Cytoplasm, SCF ubiquitin ligase complex	Cellular homeostasis, regulation of protein ubiquitination, telomere maintenance, SCF ubiquitin ligase complex, negative regulation of fibroblast proliferation, negative regulation of protein localization to nucleus
Q9CTH6	Fcf1	rRNA-processing protein FCF1 homolog	Nucleolus, small-subunit processome	Endonucleolytic cleavage in 5'-ETS of tricistronic rRNA transcript, ribosomal small subunit biogenesis
Q7TMA4	Ffar4	Free fatty acid receptor 4	Cilium, lisosomal membrane, endosome membrane, plasma membrane	Adenylate cyclase-activating G protein-coupled receptor signaling pathway, fat cell differentiation, inflammatory response, negative regulation of apoptotic process, negative regulation of cytokine production
Q91ZT5	Fgd4	FYVE	Cytoplasm, cytoskeleton, filopodium, lamellipodium	Lamellipodium assembly, microspike assembly
Q00342	Flt3	Receptor-type tyrosine-protein kinase FLT3	Endoplasmic reticulum, plasma membrane	Antigen processing and presentation, cytokine-mediated signaling pathway, cell population proliferation, protein phosphorylation
A2AED3	Fndc7	Fibronectin type III domain-containing protein 7	Extracellular region	-

Uniprot ID	Gene	Short name	Subcellular location	Gene ontology
P97376	Frg1	Protein FRG1	Nucleolus, Z disc, spliceosomal complex	mRNA processing, muscle organ development, RNA splicing
Q9Z186	G6pc2	Glucose-6-phosphatase 2	Endoplasmic reticulum	Gluconeogenesis, glucose homeostasis, regulation of insulin secretion
Q9JHE4	Gal3st1	Galactosylceramide sulfotransferase	Golgi membrane	Galactosylceramide biosynthetic process, glycerolipid metabolic process, myelination, spermatogenesis
P11862	Gas2	Growth arrest-specific protein 2	Cytoplasm	Apoptotic process, basement membrane organization, initiation of primordial ovarian follicle growth, regulation of cell cycle, regulation of cell shape, regulation of Notch signaling pathway
Q9JL56	Gde1	Glycerophosphodiester phosphodiesterase 1	Plasma membrane, cytoplasmic vesicle membrane	Ethanolamine metabolic process, G protein-coupled receptor signaling pathway, phospholipid metabolic process
Q9D7X8	Ggct	Gamma-glutamylcyclotransferase	Cytosol	Release of cytochrome c from mitochondria
Q99JY3	Gimap4	GTPase IMAP family member 4	Cytosol	-
Q75N62	Gimap8	GTPase IMAP family member 8	Cytosol, endoplasmic reticulum, Golgi apparatus, mitochondrion	Regulation of T cell apoptotic process
Q8R5M0	Gipc3	PDZ domain-containing protein GIPC3	-	-
Q6XP49	Glis3	Zinc finger protein GLIS3	Nucleus	Regulation of transcription by RNA polymerase II
P63216	Gng3	Guanine nucleotide-binding protein G(I)/G(S)/G(O) subunit gamma-3	Dendrite, cell body	G protein-coupled receptor signaling pathway, positive regulation of cytosolic calcium ion concentration
Q8C0N2	Gpat3	Glycerol-3-phosphate acyltransferase 3	Endoplasmic reticulum	CDP-diacylglycerol biosynthetic process, phospholipid biosynthetic process, regulation of TOR signaling, triglyceride biosynthetic process
Q3UFS4	Gpatch11	G patch domain-containing protein 11	Kinetochore	Nucleic acid binding
Q8BL07	Gpr150	Probable G-protein coupled receptor 150	Plasma membrane	-
Q8BXS7	Gpr152	Probable G-protein coupled receptor 152	Plasma membrane	G protein-coupled receptor activity, identical protein binding
Q8BS95	Gpr89	Golgi pH regulator	Golgi apparatus	Intracellular pH reduction, protein transport, skin development, T cell differentiation
Q8VEF1	Gramd1a	Protein Aster-A	Plasma membrane, cytosol, endoplasmic reticiulum	Autophagy, cellular response to cholesterol
P14439	H2-Ea	H-2 class II histocompatibility antigen	Plasma membrane, lysosome	Positive regulation of immune response, antigen processing and presentation of exogenous peptide antigen via MHC class II

Uniprot ID	Gene	Short name	Subcellular location	Gene ontology
P14429	H2-Q7	H-2 class I histocompatibility antigen	Endoplasmic reticulum, MHC class I protein complex	Antigen processing and presentation of peptide antigen via MHC class I, cellular response to type II interferón, embryo implantation
Q9CYQ5	Hcfc1r1	Host cell factor C1 regulator 1	Nucleoplasm, cytoplasm	-
Q8BQM4	Heatr3	HEAT repeat-containing protein 3	-	Erythrocyte maturation, protein import into nucleus, ribosomal large subunit biogenesis
P18608	Hmgn1	Non-histone chromosomal protein HMG-14	Nucleus, nucleoplasm, cytoplasm	Chromatin organization, egulation of epithelial cell proliferation, regulation of transcription by RNA polymerase II, post-embryonic camera-type eye morphogenesis
Q8R1H0	Hopx	Homeodomain-only protein	Nucleus, cytoplasm	Chaperone-mediated protein complex assembly, heart development, lung alveolus development, negative regulation of cell differentiation, negative regulation of transcription by RNA polymerase II,positive regulation of skeletal muscle tissue regeneration, regulation of heart contraction
P31257	Hoxc10	Homeobox protein Hox-C10	Nucleus	Anterior/posterior pattern specification, embryonic limb morphogenesis, neuromuscular process, proximal/distal pattern formation
P49429	Hpd	4-hydroxyphenylpyruvate dioxygenase	Endoplasmic reticulum, Golgi apparatus	L-phenylalanine catabolic process, tyrosine catabolic process
Q8BQY8	Hus1	Checkpoint protein HUS1	Nucleus, cytoplasm	Cellular response to ionizing radiation, DNA damage checkpoint signaling, regulation of protein phosphorylation
P01869	Ighg1	Ig gamma-1 chain C region	Plasma membrane, cytoplasm	Defense response to bacterium, immune response
Q64385	Il11ra1	Interleukin-11 receptor subunit alpha-1	Extracellular region	Cell population proliferation, developmental process, natural killer cell differentiation, cytokine-mediated signaling pathway
Q61098	Il18r1	Interleukin-18 receptor 1	Plasma membrane	Inflammatory response
Q99PT3	Ino80b	INO80 complex subunit B	Nucleolus	Chromatin remodeling, DNA repair, regulation of embryonic development, telomere maintenance
P01592	Jchain	Immunoglobulin J chain	Extracellular space	Adaptive immune response, glomerular filtration, innate immune response, protein-containing complex assembly
Q9JHD2	Kat2a	Histone acetyltransferase KAT2A	Nucleus	Cellular response to nerve growth factor stimulus, fibroblast proliferation, heart development, intracellular distribution of mitocondria, nervous system development, regulation of transcription by RNA polymerase II
P58391	Kcnn3	Small conductance calcium-activated potassium channel protein 3	Cytoplasm, plasma membrane, Z disc	Inward rectifier potassium channel activity, small conductance calcium-activated potassium channel activity

Uniprot ID	Gene	Short name	Subcellular location	Gene ontology
Q8BGV7	Kctd13	BTB/POZ domain-containing adapter for CUL3-mediated RhoA degradation protein 1	Nucleus, cytoplasm	Cell migration, regulation of Rho protein signal transduction, regulation of synaptic transmisión, proteasome-mediated ubiquitin-dependent protein catabolic process
Q9WU01	Khdrbs2	KH domain-containing, RNA-binding, signal transduction-associated protein 2	Nucleus	mRNA processing, regulation of mRNA splicing, via spliceosome
Q60682	Klra8	Killer cell lectin-like receptor 8	Plasma membrane	Cell adhesion
Q99KR3	Lactb2	Endoribonuclease LACTB2	Mitochondrion	RNA endonuclease activity, single-stranded RNA binding
O70263	Lnx1	E3 ubiquitin-protein ligase LNX	Cytoplasm	Protein ubiquitination, synapse maturation, ubiquitin-dependent protein catabolic process
Q91XL2	Lnx2	Ligand of Numb protein X 2	Plasma membrane	Neural precursor cell proliferation, neuron differentiation
Q921G6	Lrch4	Leucine-rich repeat and calponin homology domain-containing protein 4	Plasma membrane	Membrane raft assembly, positive regulation of toll-like receptor signaling pathway
Q8CI70	Lrrc20	Leucine-rich repeat-containing protein 20	-	-
O35125	Lrrc23	Leucine-rich repeat-containing protein 23	Cytosol, cytoskeleton	Flagellated sperm motility, radial spoke assembly
G3XA59	Lrrc32	Transforming growth factor beta activator LRRC32	Plasma membrane	Establishment of protein localization to extracellular region, negative regulation of activated T cell proliferation, negative regulation of cytokine production, transforming growth factor beta receptor signaling pathway
Q9WUC3	Ly6h	Lymphocyte antigen 6H	Plasma membrane	Acetylcholine receptor signaling pathway
P54843	Maf	Transcription factor Maf	Nucleus, nucleoplasm, cytoplasm	Cell development, megakaryocyte differentiation, regulation of transcription by RNA polymerase II
Q3UU94	Mansc4	MANSC domain-containing protein 4	Membrane	-
Q8R3I2	Mboat2	Lysophospholipid acyltransferase 2	Endoplasmic reticulum membrane	Phosphatidylcholine acyl-chain remodeling, phospholipid biosynthetic process, regulation of chondrocyte differentiation
Q9D967	Mdp1	Magnesium-dependent phosphatase 1	-	Metal ion binding, protein tyrosine phosphatase activity
Q99K74	Med24	Mediator of RNA polymerase II transcription subunit 24	Nucleus, nucleoplasm	Regulation of transcription by RNA polymerase II, somatic stem cell population maintenance
Q60929	Mef2a	Myocyte-specific enhancer factor 2A	Nucleus, nucleoplasm, cytosol	Apoptotic process, cardiac conduction, cellular response to calcium ion, DNA-templated transcription, mitochondrion distribution, negative regulation of transcription by RNA polymerase II, nervous system development, positive regulation of cardiac muscle hypertrophy
Q922T2	Mfap3	Microfibril-associated glycoprotein 3	-	Plasma membrane

Uniprot ID	Gene	Short name	Subcellular location	Gene ontology
Q3U481	Mfsd12	Major facilitator superfamily domain-containing protein 12	Endosome, lysosome, melanosome	Carbohydrate transport, cysteine transmembrane transport, melanin biosynthetic process, pigment metabolic process
Q9CQL7	Mrfap1	MORF4 family-associated protein 1	Nucleus	-
Q9Z2Q5	Mrpl40	Large ribosomal subunit protein mL40	Nucleolus, mitochondrion	Mitochondrial translation
Q8BIL2	Msantd1	Myb/SANT-like DNA-binding domain-containing protein 1	-	-
P03888	Mtnd1	NADH-ubiquinone oxidoreductase chain 1	Mitochondrion	Aerobic respiration, mitochondrial respiratory chain complex I assembly, response to hypoxia, response to organic cyclic compound
P97457	Myl11	Myosin regulatory light chain 11	Cytosol, cytoskeleton, lysosome	Immune response, muscle contraction, skeletal muscle tissue development
Q9QZ23	Nfu1	NFU1 iron-sulfur cluster scaffold homolog	Cytosol, mitochondrion	Iron-sulfur cluster assembly
P01139	Ngf	Beta-nerve growth factor	Endoplasmic reticulum, endosome, extracellular region	Axon extension, circadian rhythm, extrinsic apoptotic signaling pathway in absence of ligand, neuron projection development, positive regulation of DNA binding, positive regulation of protein phosphorylation, positive regulation of protein ubiquitination
Q6DIB4	Nol4l	Nucleolar protein 4-like	-	-
Q8R2U4	Ntmt1	N-terminal Xaa-Pro-Lys N-methyltransferase 1	Nucleus, nucleoplasm, cytosol	Chromosome segregation, spindle organization
Q9D142	Nudt14	Uridine diphosphate glucose pyrophosphatase NUDT14	Cytoplasm	ADP-ribose diphosphatase activity, identical protein binding, metal ion binding
Q6P3D0	Nudt16	U8 snoRNA-decapping enzyme	Nucleus, nucleoplasm, cytosol	Chromosome organization, mRNA catabolic process, negative regulation of rRNA processing, positive regulation of cell cycle process, sno(s)RNA catabolic process
Q8CEC0	Nup88	Nuclear pore complex protein Nup88	Nucleus, nucleoplasm	mRNA transport, nucleocytoplasmic transport, protein transport, ribosomal large subunit export from nucleus
O54943	Per2	Period circadian protein homolog 2	Nucleus, nucleoplasm, cytosol	Chromatin remodeling, circadian rhythm, fatty acid metabolic process, gluconeogénesis, negative regulation of DNA-templated transcription, negative regulation of protein ubiquitination, negative regulation of transcription regulatory region DNA binding, regulation of cell cycle, response to ischemia
Q9DAG9	Phf7	PHD finger protein 7	Cytosol, Golgi apparatusm plasma membrane	-
O54714	Pias3	E3 SUMO-protein ligase PIAS3 (EC 2.3.2.-) (E3 SUMO-protein transferase PIAS3) (Protein inhibitor of activated STAT protein 3)	Nucleus, cytoplasm	Negative regulation of DNA-templated transcription, negative regulation of osteoclast differentiation, negative regulation of transcription by RNA polymerase II

Uniprot ID	Gene	Short name	Subcellular location	Gene ontology
Q9CZX7	Pip4p2	Type 2 phosphatidylinositol 4,5-bisphosphate 4-phosphatase	Plasma membrane, endosome membrane, lysosome membrane	Negative regulation of phagocytosis, phosphatidylinositol dephosphorylation
Q04758	Pkib	cAMP-dependent protein kinase inhibitor beta	Nucleus	Negative regulation of protein kinase activity
B2RXZ1	Pnldc1	Poly(A)-specific ribonuclease PNLDC1	Endoplasmic reticulum	Blastocyst formation, nuclear-transcribed mRNA catabolic process, nonsense-mediated decay, nuclear-transcribed mRNA poly(A) tail shortening, piRNA processing
Q9D7M8	Polr2d	DNA-directed RNA polymerase II subunit RPB4	Nucleus, cytoplasm	DNA-templated transcription initiation, transcription by RNA polymerase II
Q8R4S0	Ppp1r14c	Protein phosphatase 1 regulatory subunit 14C	Cytoplasm	Regulation of phosphorylation, signal transduction
Q925E7	Ppp2r2d	Serine/threonine-protein phosphatase 2A 55 kDa regulatory subunit B delta isoform	Cytoplasm	Cell división, exit from mitosis, mitotic cell cycle
Q63810	Ppp3r1	Calcineurin subunit B type 1	Cytosol	Branching involved in blood vessel morphogenesis, epithelial to mesenchymal transition, heart development, lung epithelial cell differentiation, myelination in peripheral nervous system, negative regulation of calcium ion import across plasma membrane, positive regulation of transcription by RNA polymerase II , protein import into nucleus
Q810Y8	Pramel7	Preferentially expressed antigen in melanoma-like protein 7	-	Blastocyst formation, negative regulation of apoptotic process, negative regulation of cell differentiation, negative regulation of DNA-templated transcription, positive regulation of cell population proliferation
O70274	Ptp4a2	Protein tyrosine phosphatase type IVA 2	Plasma endosome, endosome	Protein dephosphorylation
Q9QYS9	Qki	KH domain-containing RNA-binding protein QKI	Nucleus, cytosol, cytoplasm	3'-UTR-mediated mRNA destabilization, intracellular mRNA localization, long-chain fatty acid biosynthetic process, microglia differentiation, mRNA stabilization, mRNA transport, myofibroblast contraction, negative regulation of angiogenesis, negative regulation of miRNA catabolic process,regulation of epithelial to mesenchymal transition, regulation of mRNA splicing, via spliceosome, spliceosome-depend formation of circular RNA, vascular associated smooth muscle cell differentiation, vasculogenesis
A2AU37	Rad21l1	Double-strand-break repair protein rad21-like protein 1	Nucleus	Double-strand break repair, homologous chromosome pairing at meiosis, meiotic attachment of telomere to nuclear
Q6VN19	Ranbp10	Ran-binding protein 10	Nucleus, cytoplasm, microtubule cytoskeleton	Microtubule cytoskeleton organization
P61226	Rap2b	Ras-related protein Rap-2b	Cytosol, plasma membrane, endosome	Negative regulation of cell migration, platelet aggregation regulation of protein tyrosine kinase activity

Uniprot ID	Gene	Short name	Subcellular location	Gene ontology
Q8CEE7	Rdh13	Retinol dehydrogenase 13	Mitochondrion	Eye photoreceptor cell development, retinal metabolic process
D3YUG0	Samd13	Sterile alpha motif domain-containing protein 13	Cytosol	-
Q9WU39	Scnn1g	Amiloride-sensitive sodium channel subunit gamma	Nucleoplasm, plasma membrane	Cellular response to acidic pH, cellular response to aldosterone, cellular response to vasopressin , intracellular sodium ion homeostasis,sodium ion transport
Q61112	Sdf4	45 kDa calcium-binding protein	Golgi apparatus, endosome	Cerebellum development, fat cell differentiation
Q8BMC3	Shc2	SHC-transforming protein 2	Nucleus, cytoplasm, plasma membrane	Intracellular signal transduction
Q06985	Siah1b	E3 ubiquitin-protein ligase SIAH1B	Nucleus, cytosol	Neuron apoptotic process, protein ubiquitination
P51912	Slc1a5	Neutral amino acid transporter B(0)	Plasma membrane	Glutamine transport, L-aspartate import across plasma membrane, protein homotrimerization, transport across blood-brain barrier
Q920G8	Slc25a37	Mitoferrin-1	Mitochondrion	Iron import into the mitochondrion, positive regulation of hemoglobin biosynthetic process
Q9R155	Slc26a4	Pendrin	Plasma membrane	Animal organ morphogenesis, regulation of protein localization
Q9ERH8	Slc28a3	Solute carrier family 28 member 3	Plasma membrane	Purine nucleoside transmembrane transport, pyrimidine nucleoside transport
Q3T9X0	Slc2a9	Solute carrier family 2, facilitated glucose transporter member 9	Plasma membrane, peroxisome	Glucose transmembrane transport, urate metabolic process, urate transport
Q8K0E3	Slc5a11	Sodium/myo-inositol cotransporter 2	Plasma membrane	Apoptotic process, myo-inositol transport, sodium ion transport
Q9Z1K8	Slc7a7	Y+L amino acid transporter 1	Plasma membrane	L-arginine transmembrane transport, L-leucine transport, regulation of arginine metabolic process
Q9DA21	Smco2	Single-pass membrane and coiled-coil domain-containing protein 2	Membrane	-
Q62376	Snrnp70	U1 small nuclear ribonucleoprotein 70 kDa	Nucleus	mRNA splicing, via spliceosome, regulation of RNA splicing
P62317	Snrpd2	Small nuclear ribonucleoprotein Sm D2	Nucleus, cytoplasm	mRNA splicing, via spliceosome, spliceosomal snRNP assembly
Q80W37	Snupn	Snurportin-1	Nucleoplasm, cytosol, plasma membrane	RNA, snRNA and protein import into nucleus
Q9DCI3	Stard3nl	STARD3 N-terminal-like protein	Endoplasmic reticulum, endosome	Vesicle tethering to endoplasmic reticulum
Q6J9G1	Styk1	Tyrosine-protein kinase STYK1	Plasma membrane	Protein phosphorylation
P18419	Svs4	Seminal vesicle secretory protein 4	Extracellular region	-

Uniprot ID	Gene	Short name	Subcellular location	Gene ontology
P47937	Tacr3	Neuromedin-K receptor	Dendrite membrane	Positive regulation of blood pressure, positive regulation of flagellated sperm motility, positive regulation of heart rate, positive regulation of uterine smooth muscle contraction, regulation of dopamine metabolic process
Q8K3F7	Tdh	L-threonine 3-dehydrogenase, mitochondrial	Mitochondrion	Threonine catabolic process
Q5SSF7	Tent5c	Terminal nucleotidyltransferase 5C	Nucleus, nuckeoplasm, cytoplasm, centrosome	mRNA stabilization, negative regulation of cell differentiation, in utero embryonic development
Q9D305	Thap2	THAP domain-containing protein 2	Nucleolus	-
Q8BH58	Tiprl	TIP41-like protein	Cytoplasm	DNA damage checkpoint signaling, negative regulation of phosphoprotein phosphatase activity
Q7TN58	Tmc8	Transmembrane channel-like protein 8	Nuclear membrane, plasma membrane, endoplasmic reticulum, Golgi apparatus	Intracellular zinc ion homeostasis, negative regulation of protein-containing complex assembly, positive regulation of apoptotic process, protein stabilization, regulation of extrinsic apoptotic signaling pathway via death domain receptors
Q8BQX5	Tmco6	Transmembrane and coiled-coil domain-containing protein 6	Membrane	Protein import into nucleus
Q9CR64	Tmem167a	Protein kish-A	Golgi membrane	Constitutive secretory pathway
P62996	Tra2b	Transformer-2 protein homolog beta	Nucleus, nucleoplasm	Cellular response to glucose stimulus, cerebral cortex regionalization, embryonic brain development, regulation of mRNA splicing, via spliceosome
Q9R1A9	Trex2	Three prime repair exonuclease 2	Nucleus	DNA metabolic process, DNA repair
Q99PP6	Trim34a	E3 ubiquitin-protein ligase TRIM34A	Mitochondrion	Protein ubiquitination
Q8BG34	Ubxn10	UBX domain-containing protein 10	Cilium	Cilium assembly
Q80YF6	Upk3b	Uroplakin-3b	Plasma membrane	Negative regulation of glucose import
Q9WTV6	Usp18	Ubl carboxyl-terminal hydrolase 18	Nucleus, cytosol	Protein deubiquitination, proteolysis, regulation of inflammatory response
P48615	Wnt11	Protein Wnt-11	Cytoplasm, extracellular region	Animal organ morphogenesis, apoptotic process, artery morphogenesis, atrial septum development, bicellular tight junction assembly, canonical Wnt signaling pathway, cell-cell signaling, embryonic skeletal system development, epithelial to mesenchymal transition, intracellular signal transduction, primary and secondary heart field specification

Uniprot ID	Gene	Short name	Subcellular location	Gene ontology
Q64527	Wnt8a	Protein Wnt-8a	Extracellular region	Cell morphogenesis, endoderm development, establishment of animal organ orientation, polarity specification of anterior/posterior axis, polarity specification of proximal/distal axis, Wnt signaling pathway
Q9WUD6	Wnt8b	Protein Wnt-8b	Extracellular region	Animal organ morphogenesis, cell-cell signaling, regulation of gene expression, signal transduction, Wnt signaling pathway
Q9JM98	Wrap73	WD repeat-containing protein WRAP73	Cytoplasm, centrosome	Cell projection organization, mitotic spindle assembly, positive regulation of non-motile cilium assembly
P43404	Zap70	Tyrosine-protein kinase ZAP-70	Cytosol, cytoplasm	Adaptive immune response, intracellular signal transduction, positive regulation of alpha-beta T cell differentiation, positive regulation of calcium-mediated signaling, regulation of T cell differentiation
Q91X58	Zfand2b	AN1-type zinc finger protein 2B	Endoplasmic reticulum	Proteasome-mediated ubiquitin-dependent protein catabolic process, protein targeting to ER, regulation of insulin-like growth factor receptor signaling pathway
P22227	Zfp42	Zinc finger protein 42	Nucleus, cytoplasm	Cellular response to leukemia inhibitory factor, genomic imprinting, in utero embryonic development, positive regulation of transcription by RNA polymerase II
Q99LH4	Znf672	Zinc finger protein 672	Nucleoplasm	-
Q569E7	Znf697	Zinc finger protein 697	Nucleus	-
Q62522	Zpbp	Zona pellucida-binding protein 1	Acrosomal membrane	Acrosome assembly, binding of sperm to zona pellucida
Q8BGZ8	Zpld1	Zona pellucida-like domain-containing protein 1	Cytoplasmic vesicle membrane, extracellular region	Vestibular reflex
