# Supplementary material for: Elektra-Qki and Alien-Wt1 lncRNA-protein interaction controls myocardial ion channel expression and epicardial EMT during heart development in mice
Source: Cell Mol Life Sci. 2026 Jul 7;83(1):356. doi: 10.1007/s00018-026-06322-x (PMC13631190; doi:10.1007/s00018-026-06322-x)
Supplement: Supplementary file 3 [file 18_2026_6322_MOESM12_ESM.pdf]

Uniprot ID	Gene	Short name	Subcellular location	Gene ontology
Q5DU57	Spata13	Spermatogenesis-associated protein 13	Cytoplasm, cytosol	Cell migration, filopodium assembly, intracellular signal transduction, lamellipodium assembly
Q8R0C0	Polr3gl	DNA-directed RNA polymerase III subunit RPC7-like	Nucleus	Transcription by RNA polymerase III
Q9DBT9	Dmgdh	Dimethylglycine dehydrogenase, mitochondrial	Cytoplasm, cytosol, mitochondrion	Amino-acid betaine catabolic process, choline catabolic process, tetrahydrofolate interconversion
Q9D8C8	Ppp1r35	Protein phosphatase 1 regulatory subunit 35	Cytoplasm, centriole. centrosome	Notochord morphogenesis, positive regulation of centriole elongation, positive regulation of cilium assembly
Q8BJQ2	Usp1	Ubiquitin carboxyl-terminal hydrolase 1	Nuckeus, cytoplasm, cytosol	DNA repair, protein deubiquitination, proteolysis, response to UV, skeletal system development
Q91WD9	Scgn	Secretagogin	Nucleus, cytoplasm, cytosol, dendrite	Regulation of long-term synaptic potentiation
Q8BK48	Ces2e	Pyrethroid hydrolase Ces2e	Endoplasmic reticulum	-
Q9WTN6	Slc22a21	Solute carrier family 22 member 21	Cytoplasm, cytosol	(R)-carnitine transmembrane transport, carnitine transport, monoatomic ion transport, quaternary ammonium group transport
Q80Z37	Topors	E3 ubiquitin-protein ligase Topors	Nucleus, centriole	DNA damage response, intrinsic apoptotic signaling pathway in response to DNA damage, positive regulation of DNA-templated transcription, protein monoubiquitination, regulation of cell population proliferation
Q6IFX4	Krt39	Keratin, type I cytoskeletal 39	Cytoskeleton	Epithelial cell differentiation, intermediate filament organization
O35316	Slc6a6	Taut	Cytoplasm	Amino acid import across plasma membrane, Amino acid transport, Neurotransmitter transport, Sodium ion transmembrane transport, Taurine transport
Q3TB92	Milr1	Milr1	Extracellular region, Plasma membrane	Mast cell degranulation, Negative regulation of mast cell activation
Q8K299	Scara5	Scara5	Cell surface, Dendrite, Plasma membrane, Endocytic vesicle membrane, Synaptic cleft	Cellular response to heat, Endocytosis, Intracellular iron ion homeostasis, Iron ion transmembrane transport, Protein homotrimerization
Q9Z1Y4	Trip6	Trip6	Cytoplasm, Cytoskeleton, Cytosol, Nucleus	Cell adhesion, Chordate embryonic development, Positive regulation of cell migration, Positive regulation of non-canonical NF-kappaB signal transduction, Signal transduction
Q9QUK0	Cdkl2	Cdkl2	Cytoplasm, Nucleoplasm, Nucleus	Phosphorylation

Uniprot ID	Gene	Short name	Subcellular location	Gene ontology
Q8CB77	Eloa	Elongin-A	Nucleus	Regulation of transcription by RNA polymerase II, transcription elongation by RNA polymerase II, transcription initiation at RNA polymerase II promoter
O88520	Shoc2	Leucine-rich repeat protein SHOC-2	Cytoplasm, cytosol, nucleoplasm, nucleus	Cellular response to growth hormone stimulus, negative regulation of neuron differentiation, positive regulation of neuron differentiation, positive regulation of Ras protein signal transduction, signal transduction
Q3UTA9	Eif4e1b	Eukaryotic translation initiation factor 4E type 1B	-	Translational initiation
Q6PCL9	Papolg	Poly(A) polymerase gamma	Cytosol, nucleus	Mrna polyadenylation
Q8C522	Endod1	Endonuclease domain-containing 1 protein	Extracellular region	Innate immune response
Q8BI72	Cdkn2aip	CDKN2A-interacting protein	Nucleus	DNA damage response, regulation of cell growth, protein stability
P28660	Nckap1	Nck-associated protein 1	Cytoskeleton	Apical protein localization, basal protein localization, cell migration, cortical actin cytoskeleton organization, lamellipodium assembly
Q9D0R9	Wdr89	WD repeat-containing protein 89	–	Corpus callosum development, ventricular system development
Q8CFE2	Hpf1	Histone PARylation factor 1	Nucleus, chromatin, site of DNA damage	DNA damage response, DNA repair-dependent chromatin remodeling, double-strand break repair
Q9D7S1	Tmem54	Transmembrane protein 54	Membrane	–
Q8K0Y2	Krt33a	Keratin, type I cuticular Ha3-I	Cytoskeleton, intermediate filament	Epithelial cell differentiation, intermediate filament organization
Q8K440	Abca8b	ABC-type organic anion transporter ABCA8B	Plasma membrane, endoplasmic reticulum,	Cholesterol efflux, cholesterol transport, lipid transport, positive regulation of cholesterol efflux, regulation of cholesterol efflux
O54754	Aox1	Aldehyde oxidase 1	Cytosol	Lipid metabolic process, xenobiotic metabolic process
Q3TPJ7	Midn	Midnolin	Cytosol, nucleolus, nucleus	Negative regulation of insulin secretion
Q17ST2	Kcna7	Potassium voltage-gated channel subfamily A member 7	Voltage-gated potassium channel complex	Potassium ion transmembrane transport, protein homooligomerization
Q6NZN1	Pprc1	Peroxisome proliferator-activated receptor gamma coactivator-related protein 1	Nucleoplasm, nucleus	Positive regulation of transcription by RNA polymerase II
Q8K1R7	Nek9	Serine/threonine-protein kinase Nek9	Centrosome, cytoplasm, nucleus	Cell division, mitotic cell cycle, phosphorylation, regulation of mitotic cell cycle

Uniprot ID	Gene	Short name	Subcellular location	Gene ontology
Q8R3C1	C2orf42	Uncharacterized protein C2orf42 homolog	Nucleoplasm, nucleus	–
Q91VN6	Ddx41	Probable ATP-dependent RNA helicase DDX41	Endoplasmic reticulum, nucleus, spliceosomal complex	Cell differentiation, cell population proliferation, cellular response to interferon-beta, defense response to virus, mRNA splicing, via spliceosome
Q9WTK3	Gpaa1	Glycosylphosphatidylinositol anchor attachment 1 protein	Centrosome, cytosol, GPI-anchor transamidase complex, mitochondrion, plasma membrane	Attachment of GPI anchor to protein, GPI anchor biosynthetic process, protein-containing complex assembly
Q02819	Nucb1	Nucleobindin-1	Early endosome, endoplasmic reticulum-Golgi	Regulation of protein targeting, small GTPase-mediated signal transduction
Q8BHM9	Tmprss11f	Transmembrane protease serine 11F	Cell surface, extracellular region, plasma membrane	Establishment of skin barrier, proteolysis
Q8K4L0	Ddx54	ATP-dependent RNA helicase DDX54	Golgi apparatus, nucleolus, nucleoplasm, nucleus	RNA metabolic process, RNA processing, rRNA processing
Q80XJ3	Ttc28	Tetratricopeptide repeat protein 28	Centrosome, cytoplasm, midbody, mitotic spindle, mitotic spindle midzone	Cell cycle, cell division, regulation of mitotic cell cycle
Q8JZM7	Cdc73	Parafibromin	Cdc73/Paf1 complex, cytosol, nucleus	Cell cycle, cellular response to lipopolysaccharide, endodermal cell fate commitment, mRNA 3'-end processing, negative regulation of apoptotic process
O35963	Rab33b	Ras-related protein Rab-33B	Endosome, Golgi apparatus, Golgi lumen, Golgi membrane, presynapse	Autophagosome assembly, intra-Golgi vesicle-mediated transport, negative regulation of constitutive secretory pathway, protein localization to Golgi apparatus, protein transport
P70211	Dcc	Netrin receptor DCC	Axon, cell surface, growth cone, plasma membrane, postsynaptic density	Anterior/posterior axon guidance, apoptotic process, axon guidance, cell-cell adhesion, dorsal/ventral axon guidance
Q8CHV6	Tada2a	Transcriptional adapter 2-alpha	Nucleus, mitotic spindle, chromatin complex	Chromatin organization, chromatin remodeling, mitotic cell cycle, regulation of cell cycle, regulation of cell division
Q8BZM1	Glmn	Glomulin	Cytoplasm, ubiquitin ligase complex	Cell surface receptor signaling pathway, circulatory system development, epigenetic regulation of gene expression, muscle cell differentiation, negative regulation of cell population proliferation
Q8BMD8	Slc25a24	Mitochondrial adenyl nucleotide antiporter SLC25A24	Mitochondrion, mitochondrial inner membrane, membrane	Adenine nucleotide transport, ADP transport, ATP transport, cellular response to calcium ion, cellular response to oxidative stress
Q6A085	Znf629	Zinc finger protein 629	Nucleus	Regulation of transcription by RNA polymerase II

Uniprot ID	Gene	Short name	Subcellular location	Gene ontology
Q8BZ71	Stac3	SH3 and cysteine-rich domain-containing protein 3	Cytosol, plasma membrane, nucleoplasm, synapse, T-tubule	Neuromuscular synaptic transmission, positive regulation of protein localization to plasma membrane, positive regulation of voltage-gated calcium channel activity, skeletal muscle contraction, skeletal muscle fiber development
Q9D1C9	Rrp7a	Ribosomal RNA-processing protein 7 homolog A	Cytoplasm, nucleolus, centrosome, cilium	Blastocyst formation, cilium disassembly, protein localization to nucleolus, ribosomal small subunit assembly, ribosomal small subunit biogenesis
Q8CIG0	Depdc1a	DEP domain-containing protein 1A	Nucleus	Intracellular signal transduction, negative regulation of DNA-templated transcription
A2AF53	Tmem185a	Transmembrane protein 185A	Membrane, dendrite	No GO terms available
Q8VD53	Acer2	Alkaline ceramidase 2	Golgi membrane	Cellular response to xenobiotic stimulus, ceramide catabolic process, DNA damage response, negative regulation of cell adhesion mediated by integrin, negative regulation of cell-matrix adhesion
A2A9T0	Tbkbp1	TANK-binding kinase 1-binding protein 1	Cytoplasm	Canonical NF-kappaB signal transduction, innate immune response
P23927	Cryab	Alpha-crystallin B chain	Cytoplasm, nucleus, plasma membrane, extracellular region, mitochondrion	Negative regulation of apoptotic process, response to heat, protein folding, protein stabilization, muscle organ development
Q9DBX6	Cyp2s1	Cytochrome P450 2S1	Cytoplasm, endoplasmic reticulum membrane	Xenobiotic metabolic process, prostaglandin metabolic process, retinoic acid metabolic process, epoxygenase P450 pathway
P55012	Slc12a2	Solute carrier family 12 member 2	Plasma membrane, cell projection, cytoplasm	Chloride ion homeostasis, potassium ion transport, sodium ion transport, cell volume homeostasis, response to lipopolysaccharide
Q5DQR4	Stxbp5l	Syntaxin-binding protein 5-like	Cytoplasm, plasma membrane	Regulation of exocytosis, regulation of synaptic vesicle exocytosis, glucose homeostasis, protein transport, negative regulation of insulin secretion
Q6DI92	Zswim9	ZSWIM9	-	Hematopoietic progenitor cell differentiation
Q3TZZ7	Esyt2	Extended synaptotagmin-2	Cytoplasm, endoplasmic reticulum membrane, plasma membrane	Lipid transport, endocytosis, endoplasmic reticulum-plasma membrane tethering
Q6IR34	Gpsm1	G-protein-signaling modulator 1	Plasma membrane; cytosol; ER; Golgi; nucleus	Cell differentiation; nervous system development; regulation of G protein-coupled receptor signaling; establishment of mitotic spindle orientation; intracellular signal transduction

Uniprot ID	Gene	Short name	Subcellular location	Gene ontology
P0C5E4	Ptprq	Phosphatidylinositol phosphatase PTPRQ	Plasma membrane; apical plasma membrane; cell surface	Dephosphorylation; detection of mechanical stimulus involved in sensory perception of sound; hematopoietic progenitor cell differentiation; inner ear morphogenesis; neuromuscular process controlling balance
Q9DB16	Cab39l	Calcium-binding protein 39-like MO25beta	Cytoplasm	Intracellular signal transduction; signal transduction; protein phosphorylation; kinase activity; cellular response to stimulus
Q9D8Y8	Ing5	Inhibitor of growth protein 5	Nucleus	Apoptotic signaling pathway; regulation of cell cycle; negative regulation of cell proliferation; positive regulation of apoptosis; DNA replication-dependent chromatin disassembly
Q8BK30	Ndufv3	NADH dehydrogenase flavoprotein 3 mitochondrial	Mitochondrion; mitochondrial inner membrane	Aerobic respiration; mitochondrial ATP synthesis; electron transport chain; proton motive force-driven ATP synthesis; mitochondrial respiratory chain complex I assembly
P59267	Zdhhc2	Palmitoyltransferase ZDHHC2	Endoplasmic reticulum; Golgi; Plasma membrane	Peptidyl-L-cysteine S-palmitoylation; positive regulation of synaptic potentiation; protein localization to plasma membrane; regulation of cell-cell adhesion; synapse assembly
Q8K099	Lrit1	Leucine-rich repeat immunoglobulin-like domain transmembrane protein 1	Endoplasmic reticulum membrane	(Sin anotaciones GO relevantes para extracción)
Q9QZD5	Chml	Rab proteins geranylgeranyltransferase component A 2	Cytoplasm; nucleus	Intracellular protein transport; protein geranylgeranylation; small GTPase-mediated signal transduction; vesicle-mediated transport; protein transport
P50396	Gdi1	Rab GDP dissociation inhibitor alpha	Cytoplasm; endoplasmic reticulum; Golgi	Negative regulation of axonogenesis; protein transport; Rab protein signal transduction; response to calcium ion; vesicle-mediated transport
Q91YE7	Rbm5	RNA-binding protein 5	Nucleus; nucleoplasm	Apoptotic process; mRNA splicing via spliceosome; positive regulation of apoptotic process; regulation of alternative mRNA splicing; spliceosomal complex assembly
Q6ZPJ0	Tex2	Testis-expressed protein 2	Endoplasmic reticulum; nuclear membrane	Lipid transport; intracellular lipid transport; lipid metabolic process; membrane organization; transport
Q60772	Cdkn2c	Cyclin-dependent kinase 4 inhibitor C	Cytoplasm; nucleus	Cell cycle; negative regulation of cell growth; cell population proliferation; negative regulation of stem cell proliferation; regulation of G1/S transition of mitotic cell cycle
P47226	Tes	Testin	Cytoplasm; focal adhesion	Negative regulation of cell population proliferation; cell adhesion; actin cytoskeleton organization; cell migration; signal transduction
Q6PFR5	Tra2a	Transformer-2 protein homolog alpha	Nucleus	mRNA processing; positive regulation of mRNA splicing; RNA splicing; regulation of splicing; spliceosome assembly

Uniprot ID	Gene	Short name	Subcellular location	Gene ontology
Q8CHS8	Vps37a	Vacuolar protein sorting-associated protein 37A	Cytosol; centrosome; nucleus	Protein targeting to membrane; ubiquitin-dependent protein catabolic process; protein transport; endosome sorting; vesicle-mediated transport
Q91V51	Ttll1	Polyglutamylase complex subunit TTLL1	Cytosol; extracellular region; ciliary basal body	Microtubule cytoskeleton organization; axoneme assembly; epithelial cilium movement; sperm motility; protein modification process
Q3ULW8	Parp3	Protein mono-ADP-ribosyltransferase PARP3	Cytoplasm; centrosome; nucleolus	DNA ADP-ribosylation; double-strand break repair; positive regulation of DNA ligation; telomere maintenance; regulation of mitotic spindle organization
Q5SX40	Myh1	Myosin-1	A band; cytoplasm; intercalated disc	Muscle contraction; muscle myosin complex; myosin filament; myosin II complex; cytoplasmic granule
Q8K1M6	Dnm1l	Dynamin-1-like protein	Brush border; clathrin-coated pit; cytoplasm; mitochondrion; peroxisome	Mitochondrial fission; membrane fusion; endocytosis; calcium ion transport; mitochondrial organization
Q9R1N9	Col13a1	Collagen alpha-1(XIII) chain	Cell-cell junction; collagen-containing extracellular matrix; extracellular space	Cell differentiation; cell-cell adhesion; extracellular matrix organization; skeletal system development; morphogenesis
A2BDX3	Mocs3	Adenylyltransferase and sulfurtransferase MOCS3	Cytoplasm; cytosol	Mo-molybdopterin cofactor biosynthetic process; molybdopterin cofactor biosynthetic process; protein urmylation; tRNA thio-modification; tRNA wobble uridine thiolation
P62274	Rps29	Small ribosomal subunit protein uS14	Cytoplasm; cytosol	Cytoplasmic translation; translation; ribosome; cytosolic small ribosomal subunit; ER membrane
Q80VI1	Trim56	E3 ubiquitin-protein ligase TRIM56	Cytoplasm	Defense response to virus; innate immune response; positive regulation of interferon-beta production; protein K63-linked ubiquitination; protein monoubiquitination
Q8VEL2	Mtmr14	Myotubularin-related protein 14	Perinuclear region of cytoplasm; ruffle	Dephosphorylation
Q80Y20	Alkbh8	Alkylated DNA repair protein alkB homolog 8	Cytoplasm; cytosol; nucleus	DNA damage response; tRNA methylation; tRNA wobble uridine modification
Q8BJU9	Mtrf1l	Peptide chain release factor 1-like, mitochondrial	Mitochondrion	Mitochondrial translational termination
G5E8B9	Zbtb11	Zinc finger and BTB domain-containing protein 11	Nucleolus; nucleoplasm; nucleus	Regulation of DNA-templated transcription
Q7TQF7	Amph	Amphiphysin, Amph1	Axon terminus; cytoskeleton; synaptic vesicle membrane; presynapse	Learning; positive regulation of endocytosis; synaptic vesicle endocytosis
Q8VD12	Znf385a	Zinc finger protein 385A, Hzf, Zfp385a	Chromatin; cytosol; dendrite; neuronal cell body; nucleolus; nucleus	Apoptotic process; cell cycle; DNA damage response; hemostasis; intracellular mRNA localization

Uniprot ID	Gene	Short name	Subcellular location	Gene ontology
Q8BG80	Fbxo46	F-box only protein 46, Fbxo34-like	—	—
P08101	Fcgr2	Low affinity immunoglobulin gamma Fc receptor II	Cell body; cell surface; cytoskeleton; dendritic spine; plasma membrane	Antibody-dependent cellular cytotoxicity; antigen processing via MHC II; B cell proliferation; defense response; phagocytosis
Q61495	Dsg1a	Desmoglein-1-alpha, Dsg1	Apical plasma membrane; cytoplasm; desmosome; lateral plasma membrane; nucleus	Cell-cell adhesion; homophilic cell adhesion via plasma membrane adhesion molecules
Q8BP48	Metap1	Methionine aminopeptidase 1	Cytosol	Protein maturation; proteolysis
Q8C6B2	Rtkn	Rhotekin	Actomyosin contractile ring; cytosol	Actomyosin contractile ring assembly; apoptotic process; mitotic cytokinesis; regulation of apoptotic process; Rho signaling
Q8K2Y0	Obi1	ORC ubiquitin ligase 1, RING finger protein 219	Chromatin	Positive regulation of mRNA splicing via spliceosome; protein autoubiquitination; regulation of DNA replication
Q9DAF8	—	Uncharacterized protein C4orf51 homolog	—	—
Q9CYD3	Crtap	Cartilage-associated protein	Endoplasmic reticulum; extracellular space	Chaperone-mediated protein folding; collagen fibril organization; negative regulation of post-translational protein modification; protein stabilization; spermatogenesis
P15972	Phxr2	Putative per-hexamer repeat protein 2	—	—
Q6RI63	Fam120b	Constitutive coactivator of PPAR-gamma	Nucleoplasm; nucleus	Fat cell differentiation; peroxisome proliferator activated receptor signaling pathway
Q99N92	Mrpl27	Large ribosomal subunit protein bL27m, L27mt	Cytosolic large ribosomal subunit; mitochondrial inner membrane; mitochondrion	Mitochondrial translation; translation
Q9WUH5	Trim10	Tripartite motif-containing protein 10, Herf1	Cytoplasm	Erythrocyte differentiation; innate immune response; negative regulation of viral entry; protein ubiquitination
Q8VEM9	Klhdc3	Kelch domain-containing protein 3, Peas	Chromatin; Cul2-RING ubiquitin ligase complex; cytoplasm	Meiotic cell cycle; proteasome-mediated ubiquitin-dependent protein catabolic process; protein ubiquitination
Q99K99	—	Uncharacterized protein C4orf19 homolog	—	—
Q60769	Tnfaip3	Tumor necrosis factor alpha-induced protein 3 (A20)	Cytoplasm, lysosome, nucleus	Apoptotic process, inflammatory response, negative regulation of NF-kappaB signaling, immune response regulation, protein deubiquitination, etc.
Q9JKV2	Cilk1	Serine/threonine-protein kinase ICK	Ciliary basal body, ciliary base, ciliary tip, cilium, cytoplasm, cytosol, fibrillar center, nucleus	Cilium assembly, intracellular signal transduction, protein phosphorylation, intraciliary transport

Uniprot ID	Gene	Short name	Subcellular location	Gene ontology
Q3TZ65	Cimap1d	Protein CIMAP1D (Outer dense fiber protein 3-like 2)	Cytoplasmic microtubule, cytoskeleton	-
P08551	Nefl	Neurofilament light polypeptide (NF-L)	Axon, intermediate filament, cytoplasm, myelin sheath	Axonal transport, axonogenesis, intermediate filament organization, neurofilament assembly, neuron projection morphogenesis
Q3TT99	Gdpd4	Glycerophosphodiester phosphodiesterase domain-containing protein 4	Cytoplasm, membrane	Lipid metabolic process
P16374	Zfp60	Zinc finger protein 60	Nucleus	Regulation of transcription by RNA polymerase II
P97490	Adcy8	Adenylate cyclase type 8	Plasma membrane, axon, dendrite, postsynaptic density	cAMP biosynthesis, protein kinase A activation, synaptic plasticity, memory, signal transduction
P21836	Ache	Acetylcholinesterase (AChE)	Plasma membrane, synapse, axon, neuromuscular junction	Acetylcholine catabolism, synapse assembly, receptor internalization, positive regulation of axonogenesis and dendrite morphogenesis
Q99PT9	Kif19	Kinesin-like protein KIF19	Cilium, axoneme, microtubule, kinesin complex	Microtubule depolymerization, microtubule-based movement
Q3V1G4	Olfml2b	Olfactomedin-like protein 2B	Extracellular matrix, extracellular space	Extracellular matrix organization, signal transduction
O35486	Crygs	Gamma-crystallin S	-	Lens development, epithelial morphogenesis, visual perception
Q5HZI2	C2cd4c	C2 calcium-dependent domain-containing protein 4c nuclear-localized factor 3 protein fam148c	Cytosol	-
Q8VI93	Oas3	2'-5'-oligoadenylate synthase 3	Cytoplasm; cytosol; membrane; nucleoplasm; plasma membrane	Defense response to bacterium; defense response to virus; innate immune response; MDA-5 signaling pathway; response to virus
P16460	Ass1	Argininosuccinate synthase	Cytoplasm; cytosol; mitochondrion;	Acute-phase response; arginine biosynthetic process; urea cycle; cellular response to amino acid stimulus; circadian rhythm
P08920	Cd2	T-cell surface antigen cd2	Plasma membrane;golgi apparatus; extracellular region	Cell-cell adhesion; natural killer cell activation; natural killer cell mediated cytotoxicity; positive regulation of tumor necrosis factor production; positive regulation of interleukin-8 production
Q5RJI5	Brsk1	Serine/threonine-protein kinase brsk1	Cytoplasm; nucleus; centrosome; nucleoplasm	Neuron differentiation; protein phosphorylation; associative learning; regulation of synaptic plasticity; axonogenesis
A0A0B4J1F4	Arrdc4	Arrestin domain-containing protein 4	Cytoplasm; endosome, plasma membrane; extracellular vesicle	Protein ubiquitination; extracellular vesicle biogenesis; protein transport; positive regulation of interferon-beta production; protein K63-linked ubiquitination

Uniprot ID	Gene	Short name	Subcellular location	Gene ontology
Q6PAV2	Herc4	Probable e3 ubiquitin-protein ligase herc4	Cytoplasm; cytosol; fibrillar center	Protein ubiquitination; cell differentiation; spermatogenesis; ubiquitin-dependent protein catabolic process; negative regulation of smoothened signaling pathway
Q8BZ98	Dnm3	Dynamin-3	Cytoplasm; plasma membrane; synapse; postsynaptic density; mitochondrion	Endocytosis; synapse assembly; regulation of dendritic spine morphogenesis; synaptic vesicle endocytosis; filopodium assembly
Q3TPE9	Ankmy2	Ankyrin repeat and mynd domain-containing protein 2	Cilium	-
O54825	Bysl	Bystin	Apical part of cell; cell projection; chromosome; cytoplasm; nucleolus	Blastocyst formation; in utero embryonic development; rRNA processing; stem cell proliferation; trophectodermal cell differentiation
Q6P4P1	Serpina3a	Serine protease inhibitor A3A	Extracellular space	–
P18242	Ctsd	Cathepsin D	Endosome lumen; lysosome; mitochondrion	Autophagosome assembly; insulin catabolic process; lipoprotein catabolic process; proteolysis; positive regulation of apoptotic process
Q9CY66	Gar1 Nola1	H/ACA ribonucleoprotein complex subunit 1	Box H/ACA snoRNP complex; cajal body; chromosome, telomeric region	SnoRNA guided rRNA pseudouridine synthesis; telomere maintenance via telomerase
Q9CQV8	Ywhab	14-3-3 protein beta/alpha	Cytoplasm; cytosol;	Cytoplasmic sequestering of protein; negative regulation of DNA-templated transcription; negative regulation of G protein-coupled receptor signaling; signal transduction; protein targeting
Q07139	Ect2 mKIAA4037	Protein ECT2	Bicellular tight junction; cell cortex; cytoplasm; nucleus	Activation of GTPase activity; cell differentiation; mitotic cytokinesis; protein transport; regulation of protein kinase activity
Q9D479	Uvssa Kiaa1530	UV-stimulated scaffold protein A	Chromosome; nucleoplasm	Protein ubiquitination; response to UV; transcription-coupled nucleotide-excision repair
Q9CZR2	Naalad2	N-acetylated-alpha-linked acidic dipeptidase 2	Membrane; plasma membrane	Carboxylic acid catabolic process; organic substance catabolic process; proteolysis
Q99JR6	Nmnat3	Nicotinamide/nicotinic acid mononucleotide adenylyltransferase 3	Axon; mitochondrion; neuronal cell body	NAD biosynthetic process; response to tumor necrosis factor; response to wounding
Q66L42	Map3k10	Mitogen-activated protein kinase kinase kinase 10	Cytoplasm	Negative regulation of DNA-templated transcription; phosphorylation; positive regulation of apoptotic process
Q8VC48	Pex12	Peroxisome assembly protein 12	Peroxisome	Cellular response to reactive oxygen species; peroxisome organization; protein import into peroxisome matrix; protein monoubiquitination; protein polyubiquitination
Q60900	Elavl3 Huc	ELAV-like protein 3	Ribonucleoprotein complex	Cell differentiation; nervous system development
P49194	Rbp3	Retinol-binding protein 3	Extracellular region	Proteolysis

Uniprot ID	Gene	Short name	Subcellular location	Gene ontology
Q9ESD7	Dysf Fer1l1	Dysferlin	Cytoplasm; Golgi apparatus; plasma membrane; sarcolemma; T-tubule	Angiogenesis; cellular response to osmotic stress; fat cell differentiation; muscle cell development; vesicle fusion
Q6P1I6	Psd2 Efa6c	PH and SEC7 domain-containing protein 2	Cleavage furrow; dendrite; neuronal cell body; postsynapse	Regulation of ARF protein signal transduction
Q6P9Q4	Fhod1 Fhos1	FH1/FH2 domain-containing protein 1	Cytoplasm; cytoskeleton; intercalated disc; nucleoplasm; stress fiber	Cortical actin cytoskeleton organization; establishment of centrosome localization; nuclear migration; positive regulation of stress fiber assembly; positive regulation of transcription
Q8BZR9	Ncbp3	Nuclear cap-binding protein subunit 3	Cytoplasm; nuclear cap binding complex; nuclear speck; nucleus	7-methylguanosine mRNA capping; defense response to virus; mRNA export from nucleus; transcription by RNA polymerase II; regulatory ncRNA-mediated post-transcriptional gene silencing
Q8BJH1	Zc2hc1a Fam164a	Zinc finger C2HC domain-containing protein 1A	–	–
P97287	Mcl1	Induced myeloid leukemia cell differentiation protein Mcl-1 homolog	Mitochondrial outer membrane, nucleoplasm, cytoplasm	Apoptosis, response to DNA damage, mitochondrial fusion, regulation of apoptotic process, response to cytokine
Q91YR5	Mettl13	eEF1A lysine and N-terminal methyltransferase	Nucleus, cytosol, mitochondrion	Methylation, negative regulation of G1/S transition, negative regulation of transcription, mRNA processing
Q8BKN5	Tubgcp5	Gamma-tubulin complex component 5	Centrosome, microtubules, gamma-tubulin ring complex	Microtubule organization, mitotic cell cycle, microtubule nucleation, mitotic spindle assembly
Q9R160	Adam24	Disintegrin and metalloproteinase domain-containing protein 24	Plasma membrane, sperm head	Spermatogenesis, cell differentiation, male gonad development, proteolysis
Q0ZLH2	Pjvk	Pejvakin	Peroxisomal membrane, stereocilium base, cytosol	Response to reactive oxygen species, stereocilium maintenance, pexophagy, sensory perception of sound
Q3TUA9	Pomk	Protein O-mannose kinase	Endoplasmic reticulum membrane	Brain development, O-linked glycosylation, neuromuscular process, pain perception
P52194	Clgn	Calmegin	Endoplasmic reticulum membrane	Spermatogenesis, protein folding, meiotic cell cycle, sperm–zona pellucida binding
Q497M3	Apobec4	Putative C->U-editing enzyme APOBEC-4	—	mRNA processing
Q3TJM4	Cenpt	Centromere protein T	Nucleus, centromeric region of chromosome	Cell division, kinetochore assembly, chromosome organization, chromosome segregation
Q60973	Rbbp7	Histone-binding protein RBBP7	Nucleus, NuRD complex, nucleoplasm	Chromatin remodeling, transcription regulation, brain development, stem cell regulation
Q8R197	Olah	S-acyl fatty acid synthase thioesterase, medium chain	Cytosol	Lipid biosynthesis, medium-chain fatty acid metabolism

Uniprot ID	Gene	Short name	Subcellular location	Gene ontology
Q8C8U0	Ppfibp1	Liprin-beta-1	Presynaptic active zone, plasma membrane	Neuromuscular junction development
Q9CX86	Hnrnpa0	Heterogeneous nuclear ribonucleoprotein A0	Nucleus, nucleoplasm, spliceosome	mRNA splicing, mRNA stabilization, response to LPS, inflammation
Q99J27	Slc33a1	Acetyl-coenzyme A transporter 1	Endoplasmic reticulum membrane	Acetyl-CoA transport
Q3TLI0	Trappc10	Trafficking protein particle complex subunit 10	Cytosol, TRAPPII complex	ER-to-Golgi transport, endosome-to-Golgi transport, vesicle coating
Q6P1G0	Heatr6	HEAT repeat-containing protein 6	—	—
O88685	Psmc3	26S proteasome regulatory subunit 6A	Nucleus, proteasome	Protein degradation, blastocyst development
Q7M6Y3	Picalm	Phosphatidylinositol-binding clathrin assembly protein	Early endosome, plasma membrane, synaptic vesicle	Endocytosis, endosomal transport, dendrite morphogenesis, iron homeostasis, learning and memory
P59708	Sf3b6	Splicing factor 3B subunit 6	Nucleus, spliceosome	mRNA splicing, blastocyst formation
P29352	Ptpn22	Tyrosine-protein phosphatase non-receptor type 22	Cytoplasm, nucleus	Negative regulation of t cell activation, positive regulation of erk1 and erk2 cascade, protein dephosphorylation, autophagy, regulation of leukocyte migration
Q5RI75	Rasef	Ras and EF-hand domain-containing protein homolog	Cytosol	Vesicle-mediated transport
P11033	Gzmd	Granzyme D	Cytolytic granule, cytoplasm	Killing of cells of another organism, proteolysis
Q6PDJ1	Cachd1	VWFA and cache domain-containing protein 1	Voltage-gated calcium channel complex	–
Q9D486	Cmip	C-Maf-inducing protein	Cytosol, nucleoplasm	In utero embryonic development
Q9DC71	Mrps15	Small ribosomal subunit protein uS15m	Mitochondrion, nucleoplasm	Mitochondrial translation
Q8BVN8	Dnali1	Axonemal dynein light intermediate polypeptide 1	Cilium, axoneme, dynein complex, cytoplasm	Cilium movement, sperm flagellum assembly
Q3UW53	Niban1	Protein Niban 1	Cytoplasm, plasma membrane, cytosol	Response to endoplasmic reticulum stress, positive regulation of translation, negative regulation of protein phosphorylation
Q9CQF0	Mrpl11	Large ribosomal subunit protein uL11m	Mitochondrion,	Mitochondrial translation, translation
Q9JIX8	Acin1	Apoptotic chromatin condensation inducer in the nucleus	Nucleus, nuclear speck, nucleolus, cytosol	Apoptotic process, mRNA processing, RNA splicing, erythrocyte differentiation, positive regulation of apoptotic process
Q8BMD7	Morc4	MORC family CW-type zinc finger protein 4	Nucleoplasm, nucleus	–

Uniprot ID	Gene	Short name	Subcellular location	Gene ontology
Q9WVL6	Extl3	Exostosin-like 3	Nucleus, Golgi apparatus, endoplasmic reticulum membrane, plasma membrane	Heparan sulfate proteoglycan biosynthetic process, protein glycosylation, negative regulation of inflammatory response, positive regulation of cell growth, positive regulation of pi3k/akt signaling
Q9DC71	Mrps15	Small ribosomal subunit protein uS15m	Mitochondrion, nucleoplasm	Mitochondrial translation
A2RT60	Htra4	Serine protease HTRA4	Extracellular region	Proteolysis, positive regulation of apoptotic process, programmed cell death
Q8K4Z0	Lgi2	Leucine-rich repeat LGI family member 2	Extracellular space	Inhibitory synapse assembly
Q9QVN7	Tcea2	Transcription elongation factor A protein 2	Nucleus, nucleoplasm, centrosome	Transcription elongation by rna polymerase ii, dna-templated transcription, positive regulation of transcription by rna polymerase ii
Q99LG4	Ttc5	Tetratricopeptide repeat protein 5	Nucleus, mitochondrion, cytosol, cytoplasm	DNA repair, dna damage response, positive regulation of transcription by rna polymerase ii, cellular response to starvation, positive regulation of mrna catabolic process
D3YXK2	Safb	Scaffold attachment factor B1	Nucleus	Regulation of transcription by rna polymerase ii, positive regulation of transcription by rna polymerase ii, hormone metabolic process, regulation of growth, regulation of mrna processing
P97863	Nfib	Nuclear factor 1 B-type	Nucleus, nucleoplasm, fibrillar center	Forebrain development, neurogenesis, positive regulation of transcription by rna polymerase ii, stem cell proliferation, response to wounding
Q9D6K9	Cers5	Ceramide synthase 5	Endoplasmic reticulum	Ceramide biosynthetic process, sphingolipid biosynthetic process
Q9DBA8	Amdhd1	Probable imidazolonepropionase	Cytoplasm	Histidine catabolic process; histidine catabolic process to glutamate and formamide; histidine catabolic process to glutamate and formate
Q8BFR6	Zfand1	AN1-type zinc finger protein 1	Cytoplasm; cytoplasmic stress granule	Cellular response to arsenite ion; positive regulation of intracellular protein transport; stress granule disassembly
Q8BH66	Atl1 Spg3a	Atlastin-1	Axon; cytoplasm; endoplasmic reticulum; Golgi apparatus	Axonogenesis; endoplasmic reticulum organization; endoplasmic reticulum tubular network membrane organization; protein homooligomerization
Q640R3	Hepacam	Hepatic and glial cell adhesion molecule	Axon; cell-cell junction; cytoplasm; membrane	Cell adhesion; cell cycle; protein localization; regulation of cell cycle
Q9JM52	Mink1	Misshapen-like kinase 1	Axon; cytoplasm; dendrite	Brain development; chemical synaptic transmission; dendrite morphogenesis; MAPK cascade; positive regulation of JNK cascade
Q9JLN6	Adam28	Disintegrin and metalloproteinase domain-containing protein 28	Cell surface; mitochondrion; plasma membrane	Proteolysis

Uniprot ID	Gene	Short name	Subcellular location	Gene ontology
O08539	Bin1	Myc box-dependent-interacting protein 1	Axon; axon initial segment; axon terminus; cytosol; nucleus; plasma membrane	Endosome to lysosome transport; lipid tube assembly; muscle cell differentiation; negative regulation of amyloid-beta formation; …
Q8CE13	Ccdc17	Coiled-coil domain-containing protein 17	—	—
P17047	Lamp2	Lysosome-associated membrane glycoprotein 2	Lysosome; plasma membrane; endosome	Autophagosome maturation; autophagy; cellular response to starvation; chaperone-mediated autophagy; lysosomal lumen acidification; …
O89033	Cdc6	Cell division control protein 6 homolog	Cytoplasm; nucleus; spindle	Cell division; DNA replication initiation; mitotic DNA replication checkpoint signaling; positive regulation of fibroblast proliferation
Q9ET47	Espn	Espin	Actin cytoskeleton; cytoplasm; microvillus; stereocilium	Actin filament bundle assembly; actin filament network formation; locomotory behavior; sensory perception of sound; positive regulation of filopodium assembly; negative regulation of cytoskeleton organization
Q9ES00	Ube4b	Ubiquitin conjugation factor E4 B	Cytoplasm; nucleus; ubiquitin ligase complex	ERAD pathway; protein autoubiquitination; protein folding; protein monoubiquitination; protein ubiquitination; response to ER stress; ubiquitin-dependent protein catabolic process
P04202	Tgfb1	Transforming growth factor beta-1 proprotein	Axon; cell surface; collagen-containing extracellular matrix; blood microparticle	Adaptive immune response; animal organ morphogenesis; cell activation; regulation of cell cycle; positive regulation of apoptotic process vasculogenesis
Q8BI08	Mal2	Protein MAL2	Apical plasma membrane,	Myelination
O88962	Cyp8b1	7-alpha-hydroxycholest-4-en-3-one 12-alpha-hydroxylase	Endoplasmic reticulum membrane	Bile acid biosynthetic process, Positive regulation of intestinal cholesterol absorption
Q5U901	Cpa6	Carboxypeptidase A6	Extracellular space	Proteolysis
Q8BHJ9	Slu7	Pre-mRNA-splicing factor SLU7	Catalytic step 2 spliceosome, Cytoplasm, Cytosol, Nuclear speck	Alternative mRNA splicing via spliceosome, Cellular response to heat, Intracellular protein transport, MRNA 3'-splice site recognition, RNA splicing, RNA splicing via transesterification reactions
Q8VEC3	Adgrf1	Adhesion G-protein coupled receptor F1	Cytoplasmic vesicle, Plasma membrane	Adenylate cyclase-activating G protein-coupled receptor signaling pathway, Cell surface receptor signaling pathway
Q8BQ48	Cep295	Centrosomal protein of 295 kDa	Centriole, Centrosome, Cytosol, Mitotic spindle microtubule	Centriole replication, Positive regulation of centriole elongation, Positive regulation of centrosome duplication
Q8BIF0	Cd99l2	CD99 antigen-like protein 2	Adherens junction, Cell surface, Extracellular region, Plasma membrane	Diapedesis, Homotypic cell-cell adhesion, Positive regulation of neutrophil extravasation, Positive regulation of T cell extravasation, T cell extravasation
Q6P1D7	Slx4	Structure-specific endonuclease subunit SLX4	Cell junction, Chromatin, Chromosome telomeric region, Cytosol	DNA double-strand break processing involved in repair via single-strand annealing, DNA replication,

Uniprot ID	Gene	Short name	Subcellular location	Gene ontology
Q3TKR3	Nlrp4c	NACHT, LRR and PYD domains-containing protein 4C	Cytoplasm	Inflammatory response, Regulation of inflammatory response
Q8CHK4	Kat5	Histone acetyltransferase KAT5	Chromatin, Cytoplasm, Cytosol, Kinetochore	Apoptotic process, Cellular response to estradiol stimulus, Cellular response to glucose starvation, Cellular response to glucose stimulus, DNA damage response
Q9QYS9	Qki	KH domain-containing RNA-binding protein QKI Protein quaking MqkI	Cytoplasm; Cytoplasmic stress granule; Cytosol; Nucleus; Synapse	Axon ensheathment; Intracellular mRNA localization; Long-chain fatty acid biosynthetic process; Microglia differentiation; MRNA processing
Q99JB8	Pacsin3	Protein kinase C and casein kinase II substrate protein 3	Cytoplasm; Cytosol; Endosome; Plasma membrane	Cytoskeleton organization; Endocytosis; Negative regulation of calcium ion transport; Negative regulation of endocytosis; Plasma membrane tubulation
Q9WV30	Nfat5	Nuclear factor of activated T-cells 5 NF-AT5 Rel domain-containing transcription factor NFAT5 T-cell transcription factor NFAT5	Chromosome; Cytoplasm; Cytosol; Nucleoplasm; Nucleus	Calcineurin-nfat signaling cascade; Cellular response to cytokine stimulus; DNA damage response; DNA-templated transcription
Q8BVW3	Trim14	Tripartite motif-containing protein 14 PU.1-binding protein	Cytoplasm; Mitochondrial outer membrane; Phagocytic vesicle	Inflammatory response; Innate immune response; Negative regulation of viral transcription; Protein ubiquitination
Q8BYL4	Yars2	Tyrosine--tRNA ligase mitochondrial EC 6.1.1.1 Tyrosyl-tRNA synthetase TyrRS	Cytosol; Mitochondrial matrix; Mitochondrion; Nuclear body	Mitochondrial tyrosyl-tRNA aminoacylation; TRNA aminoacylation
Q9QXB9	Drg2	Developmentally-regulated GTP-binding protein 2 DRG-2 Translation factor GTPase DRG2 TRAFAC GTPase DRG2 EC 3.6.5.-	Cytoplasm; Cytosol; Mitochondrion; Nucleoplasm	Cytoplasmic translation
Q9JLI3	Mmel1	Membrane metallo-endopeptidase-like 1 EC 3.4.24.11	Endoplasmic reticulum; Golgi apparatus; Plasma membrane	Protein processing
Q60641	Nr1h4	Bile acid receptor Farnesoid X-activated receptor Farnesol receptor HRR-1 Nuclear receptor subfamily 1 group H member 4 Retinoid X receptor-interacting protein 14 RXR-interacting protein 14	Chromatin; Euchromatin; Nucleus; Receptor complex	Bile acid and bile salt transport; Bile acid metabolic process; Bile acid signaling pathway; Cell differentiation; Cell-cell junction assembly
Q9Z2A0	Pdpk1	3-phosphoinositide-dependent protein kinase 1 mPDK1 EC 2.7.11.1	Cell projection; Cytoplasm; Cytosol; Nucleus; Perikaryon; Plasma membrane	Calcium-mediated signaling; Cell migration; Cellular response to epidermal growth factor stimulus; Epidermal growth factor receptor signaling pathway; Extrinsic apoptotic signaling pathway
Q68FH0	Pkp4	Plakophilin-4 Armadillo-related protein	Adherens junction, Cytoplasm; Cytoskeleton; Nucleus	Cell-cell adhesion; Cell-cell junction assembly; Positive regulation of cytokinesis; Positive regulation of GTPase activity
Q9ERU3	Znf22	Zinc finger protein 22 Zinc finger protein 422 Zinc finger protein Krox-25 Zinc finger protein Krox-26	Nucleoplasm; Nucleus	Embryo development; Negative regulation of transcription by RNA polymerase II; Odontogenesis; Regulation of cytokine production; Regulation of DNA-templated transcription

Uniprot ID	Gene	Short name	Subcellular location	Gene ontology
Q61627	Grid1	Glutamate receptor ionotropic delta-1 GluR delta-1 subunit	Plasma membrane; Postsynaptic membrane	Modulation of chemical synaptic transmission; Regulation of postsynapse organization; Social behavior; Synaptic transmission glutamatergic
P0C5I1	Gpr25	Probable G-protein coupled receptor 25 Gm1300	External side of plasma membrane; Plasma membrane	Calcium-mediated signaling; Cell chemotaxis; G protein-coupled receptor signaling pathway; Immune response; Positive regulation of cytosolic calcium ion concentration
A2AJ88	Pnpla7	Patatin-like phospholipase domain-containing protein 7 EC 3.1.1.	Endoplasmic reticulum	Phosphatidylcholine catabolic process
O08739	Ampd3	Amp deaminase 3	Cytosol	Adp catabolic process, Adp metabolic process, Amp catabolic process, Amp metabolic process, Atp metabolic process
O88809	Dcx	Neuronal migration protein doublecortin	Axon, Cytoplasm, Cytosol, Dendrite, Growth cone	Axon extension, Axoneme assembly, Brain development, Central nervous system projection neuron axonogenesis, Dendrite morphogenesis
Q60787	Lcp2	Lymphocyte cytosolic protein 2	Cell-cell junction, Cytosol, Plasma membrane raft, Tcr signalosome	Intracellular signal transduction, Mast cell activation, T cell receptor signaling pathway, Transmembrane receptor protein tyrosine kinase signaling pathway, Cell-cell junction
P26450	Pik3r1	Phosphatidylinositol 3-kinase regulatory subunit alpha	Cell-cell junction, Cis-Golgi network, Cytoplasm, Cytosol, Membrane	B cell differentiation, Cellular response to insulin stimulus, Cellular response to uv, Extrinsic apoptotic signaling pathway via death domain receptors, Glucose metabolic process
Q9Z0G0	Gipc1	Pdz domain-containing protein Gipc1	Brush border, Cell cortex, Cytoplasm, Cytosol, Postsynapse	Cellular response to interleukin-7, Chemical synaptic transmission, Endothelial cell migration, G protein-coupled receptor signaling pathway, Glutamate secretion
Q8BP71	Rbfox2	Rna binding protein fox-1 homolog 2	Cytoplasm, Nucleus	Dendrite morphogenesis, Intracellular estrogen receptor signaling pathway, Mrna alternative polyadenylation, Negative regulation of apoptotic process, Negative regulation of dna-templated transcription
Q8VI33	Taf9	Transcription initiation factor Tfiid subunit 9	Nucleus	Mrna transcription by rna polymerase ii, Positive regulation of dna-templated transcription, Positive regulation of transcription by rna polymerase ii
Q3UKC1	Tax1bp1	Tax1-binding protein 1 homolog	Autophagosome, Cytoplasmic vesicle, Mitochondrion	Apoptotic process, Negative regulation of apoptotic process, Negative regulation of nf-kappaB transcription factor activity
Q99PU5	Acsbg1	Long-chain-fatty-acid--coa ligase Acsbg1	Cytoplasm, Endoplasmic reticulum, Plasma membrane	Long-chain fatty acid biosynthetic process, Long-chain fatty acid metabolic process, Response to glucocorticoid, Very long-chain fatty acid metabolic process
Q571I9	Aldh16a1	Aldehyde dehydrogenase family 16 member A1	—	—

Uniprot ID	Gene	Short name	Subcellular location	Gene ontology
O89101	Fgf18	Fibroblast growth factor 18	Cytoplasm, Extracellular space, Nucleolus, Nucleus	Angiogenesis, Animal organ morphogenesis, Cell differentiation, Cell population proliferation, Chondrocyte development
Q8C4S8	Dennd2a	Denn domain-containing protein 2A	Actin cytoskeleton, Cytosol	Protein transport, Retrograde transport endosome to Golgi
A2ALS5	Rap1gap	Rap1 GTPase-activating protein 1	Axon, Cytoplasm, Dendrite, Early endosome, Golgi membrane, Membrane, Neuronal cell body	Cell-cell adhesion, Cellular response to glial cell derived neurotrophic factor, Establishment of localization in cell, Negative regulation of neuron differentiation, Negative regulation of thyroid gland epithelial cell proliferation
Q3V1M1	Igsf10	Immunoglobulin superfamily member 10	Extracellular region	Ossification, Regulation of neuron migration, Tissue regeneration
Q6PGH1	Bud31	Protein BUD31 homolog	Chromatin, Spliceosomal complex, U2-type catalytic step 2 spliceosome	Mrna splicing via spliceosome, Positive regulation of androgen receptor activity
Q9JL04	Fmn2	Formin-2	Actin filament, Cytoplasm, Endoplasmic reticulum,	Actin filament bundle assembly, Actin nucleation, Cell migration, Cellular response to hypoxia, DNA damage response
Q0GNC1	Inf2	Inverted formin-2	Perinuclear region of cytoplasm	Actin nucleation, Gene expression, Regulation of cellular component size, Regulation of mitochondrial fission
A2APF3	Ctcfl	Transcriptional repressor CTCFL	Chromosome, Cytoplasm, Cytosol, Nuclear body, Nucleus	Establishment of protein localization to chromatin, Genomic imprinting, Regulation of transcription by RNA polymerase II, Transcription initiation-coupled chromatin remodeling
Q9CWU4	C1orf52	UPF0690 protein C1orf52 homolog	Nucleoplasm	—
Q8CB44	Gramd4	GRAM domain-containing protein 4 (Death-inducing protein)	Endoplasmic reticulum membrane, Mitochondrion	Apoptotic process, Negative regulation of toll-like receptor 9 signaling
Q9DC70	Ndufs7	NADH dehydrogenase [ubiquinone] iron-sulfur protein 7, mitochondrial	Mitochondrial inner membrane,	Aerobic respiration, Electron transport coupled proton transport, Mitochondrial electron transport NADH to ubiquinone, Mitochondrial respiratory chain complex I assembly, Proton motive force-driven mitochondrial ATP synthesis
Q9ESX5	Dkc1	H/ACA ribonucleoprotein complex subunit DKC1 (Dyskerin)	Nucleolus, Nucleoplasm, Telomerase holoenzyme complex	Box H/ACA RNA 3'-end processing, Box H/ACA RNA metabolic process, Enzyme-directed rRNA pseudouridine synthesis
Q8K1K4	Cenpi	Centromere protein I (CENP-I)	Inner kinetochore, Nuclear body, Nucleus	CENP-A containing chromatin assembly, Chromosome segregation, Mitotic sister chromatid segregation
Q8BPP1	Mab21l2	Protein mab-21-like 2	Cytoplasm, Nucleus	Camera-type eye development, Cell population proliferation, Embryonic body morphogenesis, Eye development, Positive regulation of cell population proliferation

Uniprot ID	Gene	Short name	Subcellular location	Gene ontology
O08603	Raet1b	Retinoic acid early-inducible protein 1-beta	External side of plasma membrane, Extracellular space, Plasma membrane	Antigen processing and presentation of endogenous peptide antigen via MHC class I (ER pathway, TAP-independent), Cellular response to exogenous dsRNA, Defense response to bacterium,
Q6PFQ7	Rasa4	Ras GTPase-activating protein 4	Cytosol, Plasma membrane	Cellular response to calcium ion, Intracellular signal transduction, Negative regulation of Ras protein signal transduction
Q3U9G9	Lbr	Delta(14)-sterol reductase LBR	Cytoplasm, Endoplasmic reticulum membrane, Membrane, Nucleus	Cholesterol biosynthetic process, Ergosterol biosynthetic process, Neutrophil differentiation
Q3TCJ8	Ccdc69	Coiled-coil domain-containing protein 69	Cytoplasm, Midbody, Nucleus, Spindle midzone	Spindle midzone assembly
Q9CQ66	Dynlt2b	Dynein light chain Tctex-type protein 2B	Axoneme, Centrosome, Ciliary base, Cytoplasm	Cilium assembly, Intraciliary retrograde transport, Microtubule-based movement, Regulation of cilium assembly, Regulation of intraciliary retrograde transport
Q3UVK0	Ermp1	Endoplasmic reticulum metallopeptidase 1	Endoplasmic reticulum membrane	Cellular response to oxidative stress, Endoplasmic reticulum unfolded protein response, Ovarian follicle development, Proteolysis
Q9QXE2	Poll	DNA polymerase lambda	Nucleoplasm, Nucleus, Site of double-strand break	Base-excision repair (gap-filling), DNA replication, Double-strand break repair via homologous recombination, Double-strand break repair via nonhomologous end joining, Nucleotide-excision repair
Q921C5	Bicd2	Protein bicaudal D homolog 2	Cytoplasmic vesicle, Cytosol, Golgi apparatus, Nuclear envelope	Centrosome localization, Microtubule anchoring at MTOC, Microtubule-based movement, Minus-end-directed organelle transport, mRNA transport
E9PVD3	Dchs1	Protocadherin-16	Apical part of cell, Catenin complex	Anatomical structure morphogenesis, Branching involved in ureteric bud morphogenesis, Cell migration, Cell-cell adhesion, Cochlea development
Q3ZN08	Fam220a	Protein FAM220A (Acrosomal protein ACPIN1)	Nucleus	Negative regulation of transcription by RNA polymerase II
Q9WUU8	Tnip1	TNFAIP3-interacting protein 1	Cytoplasm, Nucleus	Cellular response to lipopolysaccharide, Inflammatory response, Leukocyte cell-cell adhesion, Negative regulation of canonical NF-kappaB signaling, Positive regulation of inflammatory response
Q924L1	Letmd1 Mccr	Letm1 domain-containing protein 1	Mitochondrion, Nucleolus, Nucleoplasm	Mitochondrion organization, Positive regulation of cold-induced thermogenesis, Regulation of inflammatory response, Regulation of phagocytosis
Q8QZY1	Eif3l	Eukaryotic translation initiation factor 3 subunit L	Nucleolus	Formation of cytoplasmic translation initiation complex, Translational initiation, Viral translational termination-reinitiation

Uniprot ID	Gene	Short name	Subcellular location	Gene ontology
Q9JJG0	Tacc2	Transforming acidic coiled-coil-containing protein 2	Centrosome, Cytoplasm, Nucleus	Astral microtubule organization, Cell population proliferation, Cerebral cortex development, Interkinetic nuclear migration, Mitotic spindle organization
P50586	Tub	Tubby protein	Cilium, Cytoplasm, Cytosol, Extracellular region, Nucleus	Intraciliary transport, Phagocytosis recognition, Photoreceptor cell maintenance, Positive regulation of phagocytosis, Protein localization to cilium
Q8R1F6	Hid1	Protein Hid1	Cytoplasm, Cytoplasmic microtubule, Cytoplasmic side of Golgi membrane	-
Q9D7P9	Serpinb12	Serpin B12	Extracellular space	Hematopoietic progenitor cell differentiation
P0DMR6	Gm9125	Td and Poz domain-containing protein 1-like	Cytoplasm, Nucleus	Proteasome-mediated ubiquitin-dependent protein catabolic process, Regulation of proteolysis
Q9D1Z3	Atpsckmt Fam173b	ATP synthase subunit C lysine N-methyltransferase	Mitochondrion	Peptidyl-lysine methylation, Peptidyl-lysine trimethylation, Regulation of mitochondrial ATP synthesis coupled proton transport
Q3UJP5	Cfap418	Cilia- and flagella-associated protein 418	Cytoplasm, Cytosol, Photoreceptor inner segment	Photoreceptor cell morphogenesis
Q8R0P8	Abhd8	Protein Abhd8	-	-
Q91VJ4	Stk38 Ndr1	Serine/threonine-protein kinase 38	Cytoplasm, Cytosol, Nucleus	Intracellular signal transduction, Negative regulation of map kinase activity, Postsynapse organization
O35942	Nek2	Serine/threonine-protein kinase Nek2	Centrosome, Cytoplasm, Kinetochore, Microtubule	Blastocyst development, Cell division, Centrosome separation, Chromosome segregation, Meiotic cell cycle
Q91V14	Slc12a5 Kcc2 Kiaa1176	Solute carrier family 12 member 5	Membrane, Neuronal cell body, Plasma membrane	Ammonium transmembrane transport, Cell volume homeostasis, Chemical synaptic transmission, Chloride ion homeostasis, Dendritic spine development
Q7TSH3	Znf516 Kiaa0222 Zfp516	Zinc finger protein 516	Nucleus	Adipose tissue development, Brown fat cell differentiation, Positive regulation of cold-induced thermogenesis, Positive regulation of dna-templated transcription, Response to cold
E9Q4P1	Wdfy1 Kiaa1435	WD repeat and FYVE domain-containing protein 1	Cytosol, Early endosome, Golgi apparatus, Nucleolus	Positive regulation of toll-like receptor 3 signaling pathway, Positive regulation of toll-like receptor 4 signaling pathway
Q8BMT9	Hhat Skn	Protein-cysteine N-palmitoyltransferase Hhat	Endoplasmic reticulum, Golgi apparatus,	N-terminal peptidyl-l-cysteine N-palmitoylation, Smoothened signaling pathway
P97391	Pla2g5	Phospholipase A2 group V	Extracellular region, Golgi apparatus, Plasma membrane	Arachidonic acid secretion, Cardiolipin acyl-chain remodeling, Fatty acid metabolic process, Leukotriene biosynthetic process, Negative regulation of inflammatory response
Q9JLM8	Dclk1	Serine/threonine-protein kinase DCLK1	Axon, Growth cone, Postsynaptic density	Axon extension, Axonogenesis, Brain development, Central nervous system projection neuron axonogenesis, Dendrite morphogenesis

Uniprot ID	Gene	Short name	Subcellular location	Gene ontology
Q8C4V4	Fbxl3	F-box/LRR-repeat protein 3	Cytosol, Nuclear body, Nucleoplasm, Nucleus, SCF ubiquitin ligase complex	Entrainment of circadian clock by photoperiod, Proteasome-mediated ubiquitin-dependent protein catabolic process,
Q2YDW7	Kmt5a	N-lysine methyltransferase KMT5A	Nucleus, Polytene chromosome	Cell cycle, Cell division, Negative regulation of transcription by rna polymerase ii, Peptidyl-lysine monomethylation, Regulation of DNA damage response
Q0P5V2	Sobp	Sine oculis-binding protein homolog	Nucleus	Animal organ development, Cochlea development, Cognition, Inner ear morphogenesis, Sensory perception of sound
Q80VR3	Sult1c1	Sulfotransferase 1C1	Cytoplasm	3'-phosphoadenosine 5'-phosphosulfate metabolic process, Sulfation, Sulfur compound metabolic process, Xenobiotic metabolic process
Q80TF4	Klhl13	Kelch-like protein 13	Cul3-RING ubiquitin ligase complex	Cell cycle, Cell division, Protein ubiquitination, Regulation of cytokinesis, Ubiquitination
A6H6A9	Rabgap1l	Rab GTPase-activating protein 1-like	Early endosome, Golgi apparatus, Nucleus	Endocytosis, Protein transport, Megakaryocyte development, Regulation of protein localization, Organelle organization
Q8C2E7	Washc5	WASH complex subunit 5	Cytoplasm, Endosome, Endoplasmic reticulum	Endosome organization, Endosomal transport, Actin filament polymerization, Regulation of actin nucleation, Lysosome organization
Q8BJW6	Eif2a	Eukaryotic translation initiation factor 2A	Cytoplasm,	Translation regulation, Endoplasmic reticulum stress response, Negative regulation of translation, Ribosome assembly, Translation
Q8R5G7	Arap3	Arf-GAP with Rho-GAP domain protein 3	Cytoplasm, Cytoskeleton, Lamellipodium, Plasma membrane, Ruffle	Signal transduction, Cytoskeleton regulation, Membrane dynamics, Cell organization, Cell adhesion
Q8C650	Septin10	Septin-10	Cell division site, Microtubule cytoskeleton	Cytoskeleton-dependent cytokinesis, Cell division, Microtubule organization, Cytoskeleton organization, Cell morphogenesis
Q9CQV5	Mrps24	Small ribosomal subunit protein uS3m	Mitochondrial inner membrane, Mitochondrial small ribosomal subunit, Mitochondrion	Mitochondrial translation, Ribosome assembly, Translation, Mitochondrial protein synthesis, Protein biosynthesis
P60191	Rims4	Regulating synaptic membrane exocytosis protein 4	Presynaptic active zone, Presynaptic membrane, Synapse, Synaptic membrane	Synaptic transmission regulation, Synaptic plasticity regulation, Neurotransmitter exocytosis, Synapse organization regulation, Membrane potential regulation
Q62191	Trim21	E3 ubiquitin-protein ligase TRIM21	Cytoplasm, Nucleus, Nucleoplasm	Protein ubiquitination, Innate immune response, NF-kappaB regulation, Cell cycle regulation, Regulation of viral entry
P16092	Fgfr1	Fibroblast growth factor receptor 1	Cytosol, Nucleus	Angiogenesis, Brain development, Cell proliferation, Stem cell differentiation, Fibroblast growth factor receptor signaling
P28028	Braf	Serine/threonine-protein kinase B-raf	Cytosol, Mitochondrion, Neuronal projection, Nucleus	MAPK cascade, Ras signal transduction, Cell differentiation, Gene expression regulation, Cell population proliferation

Uniprot ID	Gene	Short name	Subcellular location	Gene ontology
Q9Z2Z6	Slc25a20	Mitochondrial carnitine/acylcarnitine carrier protein	Cytosol, Mitochondrion	Carnitine transmembrane transport, In utero embryonic development, Lipid transport, Mitochondrial transport
Q99PU7	Bap1	Ubiquitin carboxyl-terminal hydrolase BAP1	Cytoplasm, Cytosol, Nucleoplasm, Nucleus	Protein deubiquitination, Cell population proliferation, Regulation of inflammatory response, Mitotic cell cycle, Gene expression
Q6PB44	Ptpn23	Tyrosine-protein phosphatase non-receptor type 23	Cytoplasm, Early endosome, Endosome, Nuclear body, Nucleus	Cilium assembly, Endocytic recycling, Dephosphorylation, Regulation of epithelial cell migration, Protein transport
P07607	Tyms	Thymidylate synthase	Cytoplasm, Cytosol, Mitochondrion, Nucleus	dTMP biosynthetic process, Methylation, Liver regeneration, Regulation of translation, Response to folic acid
Q9EQG9	Cert1	Ceramide transfer protein	Cytosol, Endoplasmic reticulum, Golgi apparatus, Mitochondrion,	Ceramide metabolic process, ER to Golgi ceramide transport, Lipid homeostasis, Endoplasmic reticulum organization, Signal transduction
Q9CPN8	Igf2bp3	Insulin-like growth factor 2 mRNA-binding protein 3	Cytoplasm, Cytoplasmic stress granule, Cytosol	Regulation of gene expression, Regulation of translation, Nervous system development, mRNA transport, mRNA stabilization
Q921L8	Galnt11	Polypeptide N-acetylgalactosaminyltransferase 11	Golgi apparatus, Golgi membrane	Protein O-linked glycosylation, Notch signaling involved in heart development, Cilium assembly, Determination of left/right symmetry, Regulation of Notch signaling pathway
P61622	Itga11	Integrin alpha-11	External side of plasma membrane, Integrin complex	Cell-matrix adhesion, Cell adhesion mediated by integrin, Cell-cell adhesion, Integrin-mediated signaling pathway, Fibroblast migration
Q9ESN9	Mapk8ip3	C-Jun-amino-terminal kinase-interacting protein 3	Axolemma, Cytoplasm, Dendrite, Golgi membrane,	JNK cascade, Axon guidance, Protein localization, Neuron differentiation, Forebrain development
Q8C525	Mb21d2	Nucleotidyltransferase MB21D2	—	—
Q91WJ8	Fubp1	Far upstream element-binding protein 1	Cytoplasm, Nucleus, Synapse	Hematopoietic stem cell proliferation, Positive regulation of Schwann cell migration, Regulation of gene expression
P18406	Ccn1	CCN family member 1	Cytosol, Extracellular space, Membrane	Cell adhesion, Apoptotic process, Extracellular matrix organization, Positive regulation of transcription by RNA polymerase II, Signal transduction
Q9D3U0	Pus10	tRNA pseudouridine synthase Pus10	Cytoplasm, Mitochondrion, Nucleus	Primary miRNA processing, tRNA pseudouridine synthesis
Q9R0L7	Akap8l	A-kinase anchor protein 8-like	Cytoplasm, Nuclear matrix,	Regulation of mRNA export from nucleus
P97390	Vps45	Vacuolar protein sorting-associated protein 45	Endosome membrane, Golgi apparatus, Golgi membrane	Intracellular protein transport, Vesicle-mediated transport
Q6NZQ2	Ddx31	ATP-dependent DNA helicase DDX31	Golgi apparatus, Nucleolus, Nucleus	Ribosome biogenesis
Q6PD29	Znf513	Zinc finger protein 513	Nucleus	Regulation of DNA-templated transcription, Retina development in camera-type eye

Uniprot ID	Gene	Short name	Subcellular location	Gene ontology
O70281	Tpst1	Protein-tyrosine sulfotransferase 1	Golgi apparatus, Golgi membrane	Post-translational protein modification
Q924A2	Cic	Protein capicua homolog	Nucleus, Protein-containing complex	Brain development, Learning, Lung alveolus development, Memory, Negative regulation of transcription by RNA polymerase II
A6H8H5	Kcnb2	Potassium voltage-gated channel subfamily B member 2	Dendrite, Neuronal cell body,	Potassium ion transport, Protein homooligomerization, Protein localization to plasma membrane
Q01514	Gbp1	Guanylate-binding protein 1	Cytosol, Golgi membrane, Plasma membrane	Activation of innate immune response, Defense response to bacterium, Cellular response to interferon-beta, Positive regulation of pyroptosis, Defense response to virus
P41183	Bcl6	B-cell lymphoma 6 protein homolog	Golgi apparatus, Nucleolus, Nucleoplasm, Nucleus	Negative regulation of apoptotic process, Cell population proliferation, Regulation of immune system process
Q9D504	Ankrd7	Ankyrin repeat domain-containing protein 7	Nucleus	Blastocyst hatching
Q2TUM3	Kcnrg	Potassium channel regulatory protein	Endoplasmic reticulum	Negative regulation of delayed rectifier potassium channel activity; Protein homooligomerization
Q80VM7	Ankrd24	Ankyrin repeat domain-containing protein 24	Plasma membrane; Stereocilium	Auditory receptor cell stereocilium organization; Sensory perception of sound
A2AIV2	Virma	Protein virilizer homolog	Cytoplasm; Nuclear speck; Nucleoplasm; Nucleus	mRNA alternative polyadenylation; mRNA methylation; RNA splicing
Q8BQR7	Mageb18	Melanoma-associated antigen B18	Cytoplasm; Nucleus	Negative regulation of transcription by rna polymerase II
Q6VZZ7	Opn5	Opsin-5	Cytoplasm; Plasma membrane	Cellular response to light stimulus; Cellular response to uv-a; Entrainment of circadian clock by photoperiod; G protein-coupled receptor signaling pathway; Visual perception
Q3UZP0	Marveld2	Marvel domain-containing protein 2	Apical plasma membrane	Bicellular tight junction assembly; Cell-cell junction organization; Establishment of endothelial barrier; Sensory perception of sound
Q9QXA7	Trim44	Tripartite motif-containing protein 44	Cytoplasm	Protein stabilization; Protein ubiquitination; Regulation of gene expression
O70299	Mab21l1	Putative nucleotidyltransferase MAB21L1	Nucleus	Eye development; Camera-type eye development; Cell population proliferation; Positive regulation of cell population proliferation
Q3UFY8	Trmt10c	tRNA methyltransferase 10 homolog C	Mitochondrion; Nucleoplasm; Nucleus	Mitochondrial tRNA methylation; Mitochondrial RNA 5'-end processing; mRNA methylation; Positive regulation of mitochondrial translation; Mitochondrial tRNA processing
Q07133	H1-6	Histone H1t	Nucleus; Nucleosome; Condensed nuclear chromosome	Spermatogenesis; Chromosome condensation; Cell differentiation; Nucleosome assembly; Negative regulation of dna recombination

Uniprot ID	Gene	Short name	Subcellular location	Gene ontology
P33534	Oprk1	Kappa-type opioid receptor	Plasma membrane; Neuronal cell body; Dendrite	G protein-coupled opioid receptor signaling pathway; Sensory perception of pain; Positive regulation of potassium ion transmembrane transport
Q60774	Tmem45a	Transmembrane protein 45A	Plasma membrane	(No biological process annotated)
Q6EBV9	Atg9b	Autophagy-related protein 9B	Cytoplasm; Endoplasmic reticulum membrane; Golgi membrane;	Autophagosome assembly; Protein localization to phagophore assembly site; Programmed necrotic cell death; Reticulophagy; Bone morphogenesis
Q8BKA3	Srrm4	Serine/arginine repetitive matrix protein 4	Nucleus	RNA splicing; Regulation of alternative mRNA splicing via spliceosome; Nervous system development; Neuron maturation; Cell differentiation
P46471	Psmc2	26S proteasome regulatory subunit 7	Dendritic spine, Proteasome	Proteasome-mediated ubiquitin-dependent protein catabolic process
Q9CWX4	Rpusd4	Pseudouridylate synthase RPUSD4, mitochondrial	Mitochondrion; Nucleoplasm;	Enzyme-directed rRNA pseudouridine synthesis; Mitochondrial tRNA pseudouridine synthesis; mRNA processing; mRNA pseudouridine synthesis; Positive regulation of mitochondrial translation
Q80V23	Znf32	Zinc finger protein 32	Nucleoplasm; Nucleus	Regulation of transcription by RNA polymerase II
P51656	Hsd17b1	17-beta-hydroxysteroid dehydrogenase type 1	Cytosol	Estrogen biosynthetic process; Testosterone biosynthetic process; Bone development; Skeletal muscle tissue development; Lipid metabolic process
Q69ZA1	Cdk13	Cyclin-dependent kinase 13	Cytosol; Golgi apparatus; Nuclear speck; Nucleus	Alternative mRNA splicing via spliceosome; Positive regulation of transcription by RNA polymerase II; Phosphorylation; Regulation of signal transduction; Hemopoiesis
Q8BVM7	Flacc1	Flagellum-associated coiled-coil domain-containing protein 1	Cytoplasm	—
Q61187	Tsg101	Tumor susceptibility gene 101 protein	Centrosome; Cytoplasm; Cytosol; Endosome membrane; Extracellular exosome; Nucleus; Plasma membrane	Cell division; Protein transport; Regulation of cell growth; Ubiquitin-dependent protein catabolic process via the multivesicular body sorting pathway; Viral budding
Q8CFH6	Sik2	Serine/threonine-protein kinase SIK2	Cytoplasm; Nucleus	Insulin receptor signaling pathway; Negative regulation of glycolytic process; Protein phosphorylation; Regulation of insulin receptor signaling pathway; Intracellular signal transduction
P70191	Traf5	TNF receptor-associated factor 5	Cytoplasm; Cytosol	Positive regulation of NF-kappaB transcription factor activity; Apoptotic process; Signal transduction; Tumor necrosis factor-mediated signaling pathway; Regulation of canonical NF-kappaB signal transduction

Uniprot ID	Gene	Short name	Subcellular location	Gene ontology
Q8K3J5	Znf131	Zinc finger protein 131	Intermediate filament cytoskeleton; Nucleoplasm	Positive regulation of transcription by RNA polymerase II; Negative regulation of transcription by RNA polymerase II; Regulation of immune system process; Regulation of cytokine production
Q8CAI1	Ccdc142	Coiled-coil domain-containing protein 142	—	—
Q60865	Caprin1	Caprin-1	Cytoplasm; Cytosol;	Generation of neurons; Synapse assembly; Regulation of mRNA localization; Nervous system development; Positive regulation of dendritic spine morphogenesis
Q8VDD8	Washc1	WASH complex subunit 1	Autophagosome; Centrosome; Cytosol	Endosomal transport; Regulation of actin filament polymerization; Retrograde transport endosome to Golgi; Regulation of immune response; Protein localization to cell surface
Q8BLD6	Ankrd55	Ankyrin repeat domain-containing protein 55	—	—
Q7M710	Tas2r125	Taste receptor type 2 member 125	Membrane	Detection of chemical stimulus involved in sensory perception of bitter taste
Q9CQQ8	Lsm7	U6 snRNA-associated Sm-like protein LSm7	Nucleus	mRNA catabolic process; mRNA splicing via spliceosome; Nuclear-transcribed mRNA catabolic process
Q9D964	Gatm	Glycine amidinotransferase, mitochondrial	Cytosol; Mitochondrion	Creatine biosynthetic process; Kidney development; Learning or memory; Muscle atrophy; Positive regulation of cold-induced thermogenesis
Q99KF0	Card14	Caspase recruitment domain-containing protein 14	Aggresome; Cytoplasm; Cytosol	Apoptotic process; Negative regulation of apoptotic process; Positive regulation of NF-kappaB transcription factor activity
Q60520	Sin3a	Paired amphipathic helix protein Sin3a	Nucleolus; Nucleoplasm; Nucleus	Activation of innate immune response; Cellular response to dopamine; Cellular response to glucose stimulus; DNA replication; Negative regulation of apoptotic process
Q8C739	Fam110b	Protein FAM110B	Centrosome; Cytosol; Mitochondrion	-
Q9CZJ2	Hspa12b	Heat shock 70 kDa protein 12B	-	-
Q501J6	Ddx17	Probable ATP-dependent RNA helicase DDX17	Cytoplasm; Cytosol; Nucleolus; Nucleus; Ribonucleoprotein complex	Alternative mRNA splicing via spliceosome; Androgen receptor signaling pathway; Defense response to virus; Epithelial to mesenchymal transition; Gene expression
Q99KR6	Rnf34	E3 ubiquitin-protein ligase RNF34	Cytoplasm; Cytosol; Endomembrane system	Apoptotic process; Cellular response to cold; Negative regulation of cysteine-type endopeptidase activity involved in execution phase of apoptosis
Q64HY3	Sp9	Transcription factor Sp9	Nucleus	Embryonic limb morphogenesis; Regulation of transcription by RNA polymerase II

Uniprot ID	Gene	Short name	Subcellular location	Gene ontology
Q05AH6	Spindoc	Spindlin interactor and repressor of chromatin-binding protein	Nucleus; Site of DNA damage	DNA damage response; Negative regulation of DNA-templated transcription; Regulation of protein ADP-ribosylation
Q69Z28	Adamts16	A disintegrin and metalloproteinase with thrombospondin motifs 16	Extracellular matrix; Extracellular region	Branching involved in ureteric bud morphogenesis; Extracellular matrix organization; Male gamete generation; Proteolysis; Regulation of cilium assembly
Q62376	Snrnp70	U1 small nuclear ribonucleoprotein 70 kDa	Nuclear speck; Nucleus;	mRNA splicing via spliceosome; Positive regulation of mRNA splicing via spliceosome; Regulation of RNA splicing
Q149F1	Rpusd2	Pseudouridylate synthase RPUSD2	-	Enzyme-directed rRNA pseudouridine synthesis; mRNA processing; mRNA pseudouridine synthesis
Q8VHQ9	Acot11	Acyl-coenzyme A thioesterase 11	Cytoplasm; Cytosol; Extracellular exosome; Mitochondrial matrix	Acyl-CoA metabolic process; Fatty acid metabolic process; Intracellular signal transduction; Negative regulation of cold-induced thermogenesis; Response to cold
Q3B807	Tcerg1l	Transcription elongation regulator 1-like protein	Nucleus	-
P53810	Pitpna	Phosphatidylinositol transfer protein alpha isoform	Cytoplasm; Cytosol; Membrane; Myelin sheath; Nucleus	Axonogenesis; Phospholipid transport
Q9R0X4	Acot9	Acyl-coenzyme A thioesterase 9, mitochondrial	Mitochondrial matrix; Mitochondrion	Acyl-CoA metabolic process; Long-chain fatty acid metabolic process; Short-chain fatty acid metabolic process
Q8R0A0	Gtf2f2	General transcription factor IIF subunit 2	Microtubule cytoskeleton;	Positive regulation of transcription by RNA polymerase II; RNA polymerase II preinitiation complex assembly; Transcription by RNA polymerase II
E9Q9F6	Catsperd	Cation channel sperm-associated auxiliary subunit delta	CatSper complex; Sperm principal piece	Flagellated sperm motility; Sperm capacitation; Spermatogenesis
Q01815	Cacna1c	Voltage-dependent L-type calcium channel subunit alpha-1C	Axon; Plasma membrane	Adult walking behavior; Calcium ion import; Calcium ion transport; Cardiac conduction; Chemical synaptic transmission
Q99P88	Nup155	Nuclear pore complex protein Nup155	Nuclear envelope; Nuclear membrane	Atrial cardiac muscle cell action potential; MiRNA processing; MRNA export from nucleus; Nuclear envelope organization; Nucleocytoplasmic transport
Q9QXX4	Slc25a13	Electrogenic aspartate/glutamate antiporter SLC25A13, mitochondrial	Mitochondrial inner membrane; Mitochondrion	Aspartate transmembrane transport; ATP biosynthetic process; Gluconeogenesis; L-glutamate transmembrane transport; Malate-aspartate shuttle
P59823	Il1rapl1	Interleukin-1 receptor accessory protein-like 1	Axon; Cell surface; Cytoplasm; Dendrite; Plasma membrane	Heterophilic cell-cell adhesion; Negative regulation of exocytosis; Neuron differentiation; Positive regulation of dendrite morphogenesis; Positive regulation of synapse assembly
P13634	Ca1	Carbonic anhydrase 1	Cytoplasm	One-carbon metabolic process

Uniprot ID	Gene	Short name	Subcellular location	Gene ontology
J3QNX5	Shisa8	Protein shisa-8	Postsynaptic membrane	Regulation of short-term neuronal synaptic plasticity
Q3UPF5	Zc3hav1	Zinc finger CCCH-type antiviral protein 1	Cytoplasm; Nucleus	Cellular response to exogenous dsRNA; Cellular response to virus
Q9D956	Marveld3	MARVEL domain-containing protein 3	Membrane	Cell-cell junction organization; Negative regulation of epithelial cell migration; Negative regulation of epithelial cell proliferation
Q9CQB5	Cisd2	CDGSH iron-sulfur domain-containing protein 2	Endoplasmic reticulum	Autophagy of mitochondrion; Regulation of autophagy
B9EJA2	Cttnbp2	Cortactin-binding protein 2	Cell cortex; Postsynaptic actin cytoskeleton	Regulation of modification of postsynaptic actin cytoskeleton; Regulation of synapse organization
Q62418	Dbnl	Drebrin-like protein	Actin filament; Cortical actin cytoskeleton	Adaptive immune response; Endocytosis; Neuron projection morphogenesis; Positive regulation of axon extension; Positive regulation of dendritic spine morphogenesis
Q9WUK2	Eif4h	Eukaryotic translation initiation factor 4H	Perinuclear region of cytoplasm	Developmental growth; Sexual reproduction
Q9D417	Fbxo24	F-box only protein 24	-	-
Q3UYG1	Ccdc160	Coiled-coil domain-containing protein 160	-	-
Q9Z0E6	Gbp2	Guanylate-binding protein 2	Cytoplasmic vesicle; Golgi apparatus; Golgi membrane	Activation of innate immune response; Cellular response to interferon-beta; Cellular response to lipopolysaccharide; Defense response to bacterium; Defense response to virus
Q922Y0	Dyrk3	Dual specificity tyrosine-phosphorylation-regulated kinase 3	Cytoplasm; Cytoskeleton; Cytosol; Nucleus	Cell cycle; Cell division; Erythrocyte differentiation; Negative regulation of apoptotic process; Protein phosphorylation
Q68FM6	Elfn2	Protein phosphatase 1 regulatory subunit 29	Extracellular matrix	-
P53810	Pitpna	Phosphatidylinositol transfer protein alpha isoform	Cytoplasm; Cytosol; Membrane; Myelin sheath; Nucleus	Axonogenesis; Phospholipid transport
Q9R0X4	Acot9	Acyl-coenzyme A thioesterase 9, mitochondrial	Mitochondrial matrix; Mitochondrion	Acyl-CoA metabolic process; Long-chain fatty acid metabolic process; Short-chain fatty acid metabolic process
Q8R0A0	Gtf2f2	General transcription factor IIF subunit 2	Microtubule cytoskeleton;	Positive regulation of transcription by RNA polymerase II; RNA polymerase II preinitiation complex assembly; Transcription by RNA polymerase II
E9Q9F6	Catsperd	Cation channel sperm-associated auxiliary subunit delta	CatSper complex; Sperm principal piece	Flagellated sperm motility; Sperm capacitation; Spermatogenesis
Q01815	Cacna1c	Voltage-dependent L-type calcium channel subunit alpha-1C	Plasma membrane	Adult walking behavior; Calcium ion import; Calcium ion transport; Cardiac conduction; Chemical synaptic transmission

Uniprot ID	Gene	Short name	Subcellular location	Gene ontology
Q99P88	Nup155	Nuclear pore complex protein Nup155	Nuclear envelope; Nuclear membrane	Atrial cardiac muscle cell action potential; MiRNA processing; MRNA export from nucleus; Nuclear envelope organization; Nucleocytoplasmic transport
Q9QXX4	Slc25a13	Electrogenic aspartate/glutamate antiporter SLC25A13, mitochondrial	Mitochondrial inner membrane; Mitochondrion	Aspartate transmembrane transport; ATP biosynthetic process; Gluconeogenesis; L-glutamate transmembrane transport; Malate-aspartate shuttle
P59823	Il1rapl1	Interleukin-1 receptor accessory protein-like 1	Cytoplasm; Dendrite; Plasma membrane	Heterophilic cell-cell adhesion; Negative regulation of exocytosis; Neuron differentiation
P13634	Ca1	Carbonic anhydrase 1	Cytoplasm	One-carbon metabolic process
J3QNX5	Shisa8	Protein shisa-8	Postsynaptic density; Postsynaptic membrane	Regulation of short-term neuronal synaptic plasticity
Q3UPF5	Zc3hav1	Zinc finger CCCH-type antiviral protein 1	Cytoplasm; Nucleus	Cellular response to exogenous dsRNA; Cellular response to virus;
Q9D956	Marveld3	MARVEL domain-containing protein 3	Membrane	Cell-cell junction organization; Negative regulation of epithelial cell migration; Negative regulation of epithelial cell proliferation; Negative regulation of JNK cascade; Protein localization to cell junction
Q9CQB5	Cisd2	CDGSH iron-sulfur domain-containing protein 2	Endoplasmic reticulum; Mitochondrial outer membrane	Autophagy of mitochondrion; Regulation of autophagy
B9EJA2	Cttnbp2	Cortactin-binding protein 2	Postsynaptic actin cytoskeleton	Regulation of modification of postsynaptic actin cytoskeleton; Regulation of synapse organization
Q62418	Dbnl	Drebrin-like protein	Actin filament; Anchoring junction	Adaptive immune response; Endocytosis; Neuron projection morphogenesis; Positive regulation of axon extension; Positive regulation of dendritic spine morphogenesis
Q9WUK2	Eif4h	Eukaryotic translation initiation factor 4H	Perinuclear region of cytoplasm	Developmental growth; Sexual reproduction
Q9D417	Fbxo24	F-box only protein 24	-	-
Q3UYG1	Ccdc160	Coiled-coil domain-containing protein 160	-	-
Q9Z0E6	Gbp2	Guanylate-binding protein 2	Cytoplasmic vesicle ; Golgi apparatus; Golgi membrane	Activation of innate immune response; Cellular response to interferon-beta
Q922Y0	Dyrk3	Dual specificity tyrosine-phosphorylation-regulated kinase 3	Cytoplasm; Cytoskeleton; Cytosol;	Cell cycle; Cell division; Erythrocyte differentiation; Negative regulation of apoptotic process; Protein phosphorylation
Q68FM6	Elfn2	Protein phosphatase 1 regulatory subunit 29	Extracellular matrix;	-
Q810U4	Nrcam	Neuronal cell adhesion molecule	Axon; Axon initial segment; Extracellular region	Angiogenesis; Axon guidance; Brain development; Cell-cell adhesion; Central nervous system development
Q9EPL9	Acox3	Peroxisomal acyl-coenzyme A oxidase 3	Mitochondrion; Peroxisomal matrix; Peroxisome	Fatty acid beta-oxidation; Fatty acid beta-oxidation using acyl-CoA oxidase; Lipid homeostasis

Uniprot ID	Gene	Short name	Subcellular location	Gene ontology
Q8CJ53	Trip10	Cdc42-interacting protein 4	Cell cortex; Cell projection; Golgi apparatus; Lysosome; Microtubule	Endocytosis; Epithelial to mesenchymal transition; Negative regulation of cardiac muscle cell apoptotic process; Positive regulation of cell growth involved in cardiac muscle cell development; Signal transduction
Q922Q9	Chid1	Chitinase domain-containing protein 1	Endomembrane system; Extracellular region; Lysosome	Carbohydrate metabolic process; Innate immune response
Q8QZY9	Sf3b4	Splicing factor 3B subunit 4	Nucleolus; Nucleus	Mrna splicing via spliceosome; Positive regulation of mrna splicing via spliceosome
Q8VII3	Cst14	Cystatin-14	Extracellular region	—
Q91X46	Arhgef3	Rho guanine nucleotide exchange factor 3	Cytoplasm	Intracellular signal transduction; Positive regulation of rho protein signal transduction
Q9JL19	Ncoa6	Nuclear receptor coactivator 6	Nucleus;	Brain development; DNA damage response; Heart development; Myeloid cell differentiation; Positive regulation of transcription by RNA polymerase II
Q9EPU4	Cpsf1	Cleavage and polyadenylation specificity factor subunit 1	Nucleoplasm; Nucleus	Mrna polyadenylation
Q9Z2I0	Letm1	Mitochondrial proton/calcium exchanger protein	Mitochondrial inner membrane; Mitochondrion	Calcium export from mitochondrion; Cristae formation; Inner mitochondrial membrane organization; Mitochondrial calcium ion homeostasis; Mitochondrial calcium ion transmembrane transport
Q9JLZ3	Auh	Methylglutaconyl-CoA hydratase mitochondrial	Mitochondrion	Fatty acid beta-oxidation; Leucine catabolic process
Q8CDT7	Cabcoco1	Ciliary-associated calcium-binding coiled-coil protein 1	Centrosome; Cytoplasm; Sperm flagellum	—
Q9D9V7	Dennd6b	Protein DENND6B (DENN domain-containing protein 6B)	Recycling endosome	—
Q8CIT9	Sbsn	Suprabasin	Cytoplasm; Extracellular region	—
Q8R332	Nup58	Nucleoporin p58/p45	Nucleus; Nuclear envelope	MRNA transport; Nucleocytoplasmic transport; Protein transport; Regulation of protein import into nucleus; Nuclear pore assembly
Q8BS35	Agmo	Alkylglycerol monooxygenase	Endoplasmic reticulum	Ether lipid metabolic process; Membrane lipid metabolic process; Triglyceride biosynthetic process; Lipid oxidation; Membrane organization
Q5BKS4	Eri2	ERI1 exoribonuclease 2	Nucleus	Exonucleolytic trimming to generate mature 3'-end of 5.8S rRNA; RNA processing; RNA metabolism; RNA maturation; RNA binding
E9Q0C6	Kiaa1210	Acrosomal protein KIAA1210	Cytoplasm	Acrosomal vesicle organization; Spermatogenesis; Vesicle-mediated transport; Fertilization; Cell differentiation

Uniprot ID	Gene	Short name	Subcellular location	Gene ontology
O54972	Cbfa2t3	Protein CBFA2T3	Nucleus; Golgi apparatus	DNA-templated transcription; Granulocyte differentiation; Negative regulation of cell population proliferation; Regulation of transcription; Proteasomal ubiquitin-dependent protein catabolic process
Q9CPR5	Mrpl15	Large ribosomal subunit protein uL15m	Mitochondria	Mitochondrial translation; Mitochondrial genome maintenance; Translation; Cellular response to leukemia inhibitory factor; Ribosome assembly
Q5F2E7	Nufip2	FMR1-interacting protein NUFIP2	Nucleus; Cytoplasm	RNA binding; Ribosome biogenesis; Stress granule assembly; RNA processing; Protein binding
Q8CIM8	Ints4	Integrator complex subunit 4	Nucleus; Endosome	Intercellular transport; snRNA processing; RNA splicing; RNA maturation; RNA polymerase II activity
O88544	Cops4	COP9 signalosome complex subunit 4	Cytoplasm; Nucleus	Protein deneddylation; Regulation of ubiquitin-dependent protein catabolic process; Signal transduction; Protein complex assembly; Cellular response to stress
O88543	Cops3	COP9 signalosome complex subunit 3	Cytoplasm; Nucleus	Embryonic development; Protein deneddylation; Regulation of DNA damage response; Ubiquitin-dependent protein catabolic process; Transcription regulation
Q06890	Clu	Clusterin	Cytoplasm; Extracellular space	Cell morphogenesis; Protein folding; Apoptotic process regulation; Immune response; Protein stabilization
Q91YE3	Egln1	Egl nine homolog 1	Cytoplasm; Nucleus	Cellular response to hypoxia; Cardiac muscle tissue morphogenesis; Regulation of angiogenesis; Apoptotic process; Intracellular iron ion homeostasis
Q3U6K5	Spata6	Spermatogenesis-associated protein 6	Extracellular region; Cytoplasm	Cell differentiation; Motile cilium assembly; Spermatogenesis; Cytoskeleton organization; Reproductive process
Q497V6	Bahd1	Bromo adjacent homology domain-containing 1	Nucleus	Heterochromatin formation; Negative regulation of transcription; Chromatin silencing; DNA binding; Epigenetic regulation
Q8K1S3	Unc5b	Netrin receptor UNC5B	Plasma membrane; Cytoplasm	Angiogenesis; Axon guidance; Apoptotic process; Signal transduction; Negative regulation of neuron apoptotic process
Q64521	Gpd2	Glycerol-3-phosphate dehydrogenase, mitochondrial	Mitochondrion, Mitochondrial inner membrane	Gluconeogenesis; Glycerol catabolic process; NADH metabolic process; Camera-type eye development; Multicellular organism growth
E9PV24	Fga	Fibrinogen alpha chain	Extracellular space, Blood microparticle	Blood coagulation; Adaptive immune response; Fibrinolysis; Platelet aggregation; Innate immune response
Q80VR2	Ell3	RNA polymerase II elongation factor ELL3	Nucleus, Cytosol	Positive regulation of transcription by RNA polymerase II; Neural precursor cell proliferation; Stem cell proliferation; Transcription elongation; Signal transduction by p53

Uniprot ID	Gene	Short name	Subcellular location	Gene ontology
Q8BUH1	Txnl4b	Thioredoxin-like protein 4B	Nucleus, Cytosol	Cell cycle; Positive regulation of cell population proliferation; mRNA splicing via spliceosome
Q6R0H7	Gnas	Guanine nucleotide-binding protein G(s) subunit alpha isoforms XLas	Cytoplasm, Plasma membrane	G protein-coupled receptor signaling pathway; Positive regulation of insulin secretion; Skeletal system development; Cellular response to glucagon stimulus; Adenylate cyclase-activating dopamine receptor signaling pathway
P59383	Lrrn4	Leucine-rich repeat neuronal protein 4	Plasma membrane, Extracellular space	Long-term memory; Visual learning
Q9DAM9	Fank1	Fibronectin type 3 and ankyrin repeat domains 1 protein	Nucleus, Cytosol	Positive regulation of DNA-binding transcription factor activity; Positive regulation of DNA-templated transcription
Q80ZD9	Amigo2	Amphoterin-induced protein 2	Plasma membrane, Membrane, Nucleus	Cell adhesion; Brain development; Positive regulation of synapse assembly
Q9QYC7	Ggcx	Vitamin K-dependent gamma-carboxylase	Endoplasmic reticulum membrane	Vitamin K metabolic process
Q8K0Q5	Arhgap18	Rho GTPase-activating protein 18	Cytoplasm, Plasma membrane	Regulation of actin filament polymerization; Regulation of cell motility; Regulation of cell shape; Small GTPase-mediated signal transduction; Regulation of small GTPase mediated signal transduction
Q91WG7	Dgkg	Diacylglycerol kinase gamma	Cytosol, Plasma membrane	Intracellular signal transduction; Neuron development; Lipid phosphorylation; Regulation of dendrite development; Negative regulation of protein kinase C signaling
Q8C172	Cers6	Ceramide synthase 6	Endoplasmic reticulum	Ceramide biosynthetic process; Sphingolipid biosynthetic process; Inflammatory response; Positive regulation of oligodendrocyte apoptotic process
P0C6A2	Mamld1	Mastermind-like domain-containing protein 1	Golgi apparatus, Centrosome	Male gonad development; Regulation of transcription by RNA polymerase II
Q8K375	Lrrc56	Leucine-rich repeat-containing protein 56	Cilium	Cell projection organization
P97384	Anxa11	Annexin A11	Cytoplasm, Cytosol, Plasma membrane	Cytokinetic process; Phagocytosis; Response to calcium ion
Q8CHS7	Dhrs7c	Dehydrogenase/reductase SDR family member 7C	Sarcoplasmic reticulum	Glucose import; Intracellular calcium ion homeostasis; Regulation of release of sequestered calcium ion into cytosol
P60330	Espl1	Separin	Nucleus, Centrosome	Chromosome segregation; Meiotic chromosome separation; Mitotic cell cycle; Proteolysis; Mitotic sister chromatid segregation
O35652	Lhx8	LIM/homeobox protein Lhx8	Nucleus	Female gonad development; Forebrain neuron development; Learning or memory; Neuron differentiation; Regulation of transcription by RNA polymerase II
Q3UJB9	Edc4	Enhancer of mRNA-decapping protein 4	Nucleus	Deadenylation-independent decapping of nuclear-transcribed mRNA

Uniprot ID	Gene	Short name	Subcellular location	Gene ontology
P22561	Wt1	Wilms tumor protein homolog	Nucleus, Cytoplasm	Gonad development; Kidney development; Negative regulation of apoptotic process; Positive regulation of transcription by RNA polymerase II; Sertoli cell development
Q4KL35	Magix	PDZ domain-containing protein MAGIX	Cytoplasm	-
Q9CR62	Slc25a11	Mitochondrial 2-oxoglutarate/malate carrier protein	Mitochondria, Inner membrane	Gluconeogenesis, Lipid transport, Malate transmembrane transport, Oxaloacetate transport, Phosphate ion transmembrane transport
Q8CG70	P3h3	Prolyl 3-hydroxylase 3	Endoplasmic reticulum	Collagen biosynthetic process, Collagen metabolic process, Negative regulation of cell proliferation, Peptidyl-lysine hydroxylation
Q3UCV8	Otulin	Ubiquitin thioesterase otulin	Cytoplasm	Innate immune response, Negative regulation of inflammatory response,
Q8R5M0	Gipc3	PDZ domain-containing protein GIPC3	Cytoplasm	-
Q6T707	Scd4	Stearoyl-CoA desaturase 4	Endoplasmic reticulum, Membrane	Cellular response to nutrient, Fatty acid biosynthetic process, Monounsaturated fatty acid biosynthetic process, Response to fatty acid
P39061	Col18a1	Collagen alpha-1(XVIII) chain	Extracellular matrix, Basement membrane	Angiogenesis, Blood vessel endothelial cell migration, Cell adhesion, Collagen fibril organization, Endothelial cell apoptotic process
Q3UFY7	Nt5c3b	7-methylguanosine phosphate-specific 5'-nucleotidase	Cytoplasm	Nucleotide metabolic process
Q9ET78	Jph2	Junctophilin-2	Membrane, Nucleus	Calcium ion homeostasis, Positive regulation of cytosolic calcium concentration, Regulation of cardiac muscle development
Q9WUU9	Ganp	Germinal-center associated nuclear protein	Nucleus, Cytoplasm	mRNA export from nucleus, Nucleosome organization, Poly(A)+ mRNA export, Protein transport, Somatic hypermutation of immunoglobulin genes
Q99PM9	Uck2	Uridine-cytidine kinase 2	Cytoplasm	CTP salvage, Phosphorylation, UMP salvage
Q64311	Ntan1	Protein N-terminal asparagine amidohydrolase	Cytoplasm, Nucleus	Adult locomotory behavior, Memory, Ubiquitin-dependent protein catabolic process
Q8BLG0	Phf20	PHD finger protein 20	Nucleus, Cytosol	Chromatin organization, Positive regulation of DNA-templated transcription, Regulation of transcription by RNA polymerase II,
P35441	Thbs1	Thrombospondin-1	Extracellular matrix, Cell surface	Angiogenesis, Apoptotic process, Behavioral response to pain, Blood vessel morphogenesis, Inflammatory response
Q99KD5	Unc45a	Protein unc-45 homolog A	Cytoplasm, Nucleus	Cell differentiation, Chaperone-mediated protein folding, Muscle organ development

Uniprot ID	Gene	Short name	Subcellular location	Gene ontology
O88987	Akap3	A-kinase anchor protein 3	Cytoplasm, Motile cilium	Blastocyst hatching, Protein localization, Transmembrane receptor protein serine/threonine kinase signaling pathway
Q99M74	Krt82	Keratin, type II cuticular Hb2	Extracellular space	Intermediate filament organization, Keratinization
Q3U0L2	Ankrd33b	Ankyrin repeat domain-containing protein 33B	-	-
P97295	Npy2r	Neuropeptide Y receptor type 2	Plasma membrane, Cilium	Adenylate cyclase-inhibiting G protein-coupled receptor signaling pathway, Behavioral fear response, G protein-coupled receptor signaling pathway
Q8BG58	P4htm	Transmembrane prolyl 4-hydroxylase	Endoplasmic reticulum	Regulation of erythrocyte differentiation
Q7TMR0	Prcp	Lysosomal Pro-X carboxypeptidase	Lysosome, Basal part of cell	Angiogenesis involved in wound healing, Energy homeostasis, Glucose homeostasis, Negative regulation of systemic arterial blood pressure
P48615	Wnt11	Protein Wnt-11	Cytoplasm, Extracellular matrix	Adrenal gland development, Animal organ morphogenesis, Apoptotic process, Artery morphogenesis, Canonical Wnt signaling pathway
P61211	Arl1	ADP-ribosylation factor-like protein 1	Golgi apparatus, Cytoplasm	Golgi organization, Golgi vesicle transport, Intracellular protein transport, Protein localization to Golgi apparatus, Vesicle-mediated transport
P59997	Kdm2a	Lysine-specific demethylase 2A	Nucleoplasm, Chromatin	Circadian regulation of gene expression, Heart looping, Negative regulation of apoptotic process, Neuron differentiation, Regulation of transcription by RNA polymerase II
P70248	Myo1f	Unconventional myosin-If	Cytoplasm, Actin cytoskeleton	Actin filament organization, Defense response to Gram-positive bacterium, Endocytosis, Neutrophil degranulation, Neutrophil mediated immunity
Q3UY90	Gask1a	Golgi-associated kinase 1A	Golgi apparatus, Endoplasmic reticulum	-
Q9EPW9	Tlr6	Toll-like receptor 6	Golgi apparatus, Membrane raft	Inflammatory response, Innate immune response, Toll-like receptor signaling pathway, Positive regulation of cytokine production involved in inflammatory response
Q8K4K2	Trib3	Tribbles homolog 3	Nucleus, Nucleoplasm	Cellular response to insulin stimulus, Intrinsic apoptotic signaling pathway in response to ER stress,
Q8BYI8	Fam234b	Protein FAM234B	Golgi apparatus, Membrane	-
Q80VW7	Akna	Microtubule organization protein AKNA	Centrosome, Centriole	Delamination, Epithelial to mesenchymal transition, Neuroblast delamination
Q6P1G2	Kdm2b	Lysine-specific demethylase 2B	Nucleus, Chromosome, Nucleolus	Embryonic camera-type eye morphogenesis, Forebrain development, Negative regulation of neural precursor cell proliferation

Uniprot ID	Gene	Short name	Subcellular location	Gene ontology
Q6ZWU9	Rps27	Small ribosomal subunit protein eS27	Cytosol, Nucleolus	Ribosomal small subunit assembly, Ribosomal small subunit biogenesis, rRNA processing, Translation at presynapse, Translation at postsynapse
P62761	Vsnl1	Visinin-like protein 1	Cytosol, Membrane	Negative regulation of insulin secretion, Positive regulation of exocytosis, Positive regulation of insulin secretion involved in glucose stimulus
O08918	Ccng2	Cyclin-G2	Nucleus, Cytoplasm	Cell division, Mitotic cell cycle phase transition, Regulation of cell cycle
O70303	Cideb	Lipid transferase CIDEB	Endoplasmic reticulum, Cytosol, Golgi apparatus	Apoptotic process, Lipid droplet fusion, Regulation of apoptotic process, Regulation of triglyceride metabolic process, Very-low-density lipoprotein particle assembly
Q66X19	Nlrp4e	NACHT, LRR and PYD domains-containing protein 4E	Cytoplasm	Inflammatory response, Regulation of inflammatory response
Q9D695	Serpinb7	Serpin B7	Cytoplasm, Extracellular space	Positive regulation of collagen biosynthetic process, Positive regulation of glomerular mesangial cell proliferation, Positive regulation of platelet-derived growth factor production
Q9D773	Mrpl2	Large ribosomal subunit protein uL2m	Mitochondrion, Cytosolic large ribosomal subunit	Cytoplasmic translation, Mitochondrial translation, Translation
Q9DCC7	Isoc2	Isochorismatase domain-containing protein 2B	Cytoplasm, Mitochondrion, Nucleus	-
Q9D5I4	Dynlt5	Dynein light chain Tctex-type 5	Cytoplasm	Microtubule-based movement
P49282	Slc11a2	Natural resistance-associated macrophage protein 2	Plasma membrane, Golgi	Iron ion transport; Metal ion transport; Response to hypoxia; Ion homeostasis; Heme biosynthetic process
Q6PGA0	Rcor3	REST corepressor 3	Nucleus	Negative regulation of transcription; Regulation of transcription; DNA templated transcription; RNA polymerase II activity
Q6W3F0	P4ha3	Prolyl 4-hydroxylase subunit alpha-3	Endoplasmic reticulum	-
Q8BTI9	Pik3cb	Phosphatidylinositol 4,5-bisphosphate 3-kinase catalytic subunit beta isoform	Cytoplasm, Plasma membrane	Signal transduction; Cell migration; Phosphorylation; Angiogenesis; Autophagy
Q4G0F8	Ubn1	Ubinuclein-1	Nucleus	Nucleosome assembly; Keratinocyte differentiation
Q4VAA7	Snx33	Sorting nexin-33	Cytoplasm, Plasma membrane	Endocytosis; Protein transport; Macropinocytosis; Endosome organization; Negative regulation of endocytosis
Q6KAU4	Mvb12b	Multivesicular body subunit 12B	Cytosol, Early endosome	Protein transport; Regulation of signaling pathway; Viral budding; Virus maturation
Q9Z1T1	Ap3b1	AP-3 complex subunit beta-1	Mitochondrion, Trans-Golgi	Intracellular transport; Antigen processing; Vesicle-mediated transport; Blood coagulation; Cell morphogenesis
Q8CGE9	Rgs12	Regulator of G-protein signaling 12	Cytoplasm, Plasma membrane	Signal transduction; Regulation of G protein-coupled receptor signaling; Termination of G protein signaling

Uniprot ID	Gene	Short name	Subcellular location	Gene ontology
Q8BL48	Unk	RING finger protein unkempt homolog	Cytoplasm	Cell morphogenesis; Neuron migration; Negative regulation of translation; Embryonic development; --
Q01320	Top2a	DNA topoisomerase 2-alpha	Nucleus, Cytoplasm	Chromosome segregation; DNA damage response; Apoptotic process regulation; DNA ligation; Meiotic recombination
Q8BNU0	Armc6	Armadillo repeat-containing protein 6	Cytosol	Hematopoietic progenitor cell differentiation
P29754	Agtr1a	Type-1 angiotensin II receptor A	Plasma membrane, Golgi	Blood vessel development; Regulation of blood pressure; G protein-coupled receptor signaling; Positive regulation of angiogenesis; Inflammatory response
Q80UP5	Ankrd13a	Ankyrin repeat domain-containing protein 13A	Cytoplasm, Plasma membrane	Negative regulation of receptor internalization; Negative regulation of protein localization
Q3UH06	Rreb1	Ras-responsive element-binding protein 1	Nucleus	Negative regulation of transcription; Positive regulation of epithelial cell migration; Regulation of transcription by RNA polymerase II
P21183	Il5ra	Interleukin-5 receptor subunit alpha	Plasma membrane	Cytokine-mediated signaling pathway; Cellular response to organic substance; Inflammatory response; Regulation of interleukin-5 production
P50427	Sts	Steryl-sulfatase	Endoplasmic reticulum membrane, Nuclear envelope	Steroid metabolic process; Response to estrogen; Positive regulation of cell proliferation; Learning or memory; Response to peptide hormone
Q8VCE9	Plekhh3	Pleckstrin homology domain-containing family H member 3	Cytoskeleton	Signal transduction
Q8BU88	Mrpl22	Large ribosomal subunit protein uL22m	Mitochondrion	Mitochondrial translation; Translation
Q8K009	Aldh1l2	Mitochondrial 10-formyltetrahydrofolate dehydrogenase	Mitochondrion, Nucleoplasm	Fatty acid beta-oxidation; Folic acid metabolic process; Biosynthetic process; NADPH regeneration; One-carbon metabolic process
Q3V1D5	Pnpla1	Omega-hydroxyceramide transacylase	Cytoplasm, Lipid droplet, Membrane	Ceramide biosynthetic process, Establishment of skin barrier, Keratinocyte differentiation, Lipid homeostasis, Triglyceride catabolic process
Q8R1F9	Rpp40	Ribonuclease P protein subunit p40	Nucleolus, Ribonuclease P complex	Endonucleolytic cleavage in ITS1, TRNA 5'-leader removal, RNA processing, RNA metabolic process, RNA splicing
P62315	Snrpd1	Small nuclear ribonucleoprotein Sm D1	Nucleus, Cytoplasm	MRNA splicing via spliceosome, Spliceosomal snRNP assembly, RNA processing, RNA splicing, RNA metabolic process
Q68FD9	Kiaa1549	UPF0606 protein KIAA1549	Membrane, Photoreceptor connecting cilium	-
P22682	Cbl	E3 ubiquitin-protein ligase CBL	Cytosol, Plasma membrane, Golgi apparatus	Protein ubiquitination, Signal transduction, Negative regulation of apoptotic process, Response to hypoxia, Protein phosphorylation

Uniprot ID	Gene	Short name	Subcellular location	Gene ontology
Q80YY7	Znf618	Zinc finger protein 618	Nucleus, Heterochromatin	-
Q8K450	Spag16	Sperm-associated antigen 16 protein	Axoneme, Extracellular region, Sperm flagellum	Axoneme assembly, Cilium assembly, Sperm axoneme assembly, Mucociliary clearance, Cerebrospinal fluid circulation
Q9D3R3	Cep72	Centrosomal protein of 72 kDa	Centrosome, Cytoplasm	Centriole replication, Spindle organization, Gamma-tubulin complex localization, Protein localization to centrosome, Cell cycle process
B1ARD8	Slfn8	Schlafen family member 8	Cytoplasm	Defense response to virus, Immune system process, RRNA catabolic process, TRNA catabolic process, RNA metabolic process
Q9Z2H7	Gipc2	PDZ domain-containing protein GIPC2	Cytoplasm	-
Q924W6	Trim66	Tripartite motif-containing protein 66	Nucleus, Chromocenter	Negative regulation of DNA-templated transcription, Chromatin remodeling, Regulation of transcription, Gene expression, Cellular response to DNA damage
Q69Z66	Mysm1	Deubiquitinase MYSM1	Nucleus, Cytoplasm, Nucleolus	Chromatin remodeling, Proteolysis, Positive regulation of transcription by RNA polymerase II, Immune system process, Regulation of cell migration
P51860	Nap1l2	Nucleosome assembly protein 1-like 2	Nucleus, Chromatin	Neuron differentiation, Nucleosome assembly, Positive regulation of neuron differentiation, Regulation of stem cell division, Chromatin organization
Q6GQX2	Nckap5l	Nck-associated protein 5-like	Centrosome, Cytoplasm	Microtubule bundle formation, Microtubule depolymerization
Q62318	Trim28	Transcription intermediary factor 1-beta	Chromatin, Nucleus	Chromatin organization, DNA repair, Innate immune response, Protein sumoylation, Regulation of transcription by RNA polymerase II
Q91XS1	Mtmr4	Myotubularin-related protein 4	Cytoplasm, Membrane	Phosphatidylinositol dephosphorylation, Regulation of autophagy, Response to denervation involved in regulation of muscle adaptation
P27656	Lipc	Hepatic triacylglycerol lipase	Cell surface, Extracellular	Cholesterol homeostasis, Cholesterol metabolic process, Triglyceride metabolic process, Fatty acid metabolic process, Regulation of plasma lipoprotein particle levels
P97500	Myt1l	Myelin transcription factor 1-like protein	Nucleus, Chromosome	Nervous system development, Neuron differentiation, Regulation of transcription by RNA polymerase II
Q02248	Ctnnb1	Catenin beta-1	Cytoplasm, Cell junction	Cell adhesion, Canonical Wnt signaling pathway, Cell differentiation, Regulation of gene expression, Nervous system development

Uniprot ID	Gene	Short name	Subcellular location	Gene ontology
O35250	Exoc7	Exocyst complex component 7	Cytosol, Plasma membrane	Exocytosis, Membrane fission, Mitotic cytokinesis, Protein transmembrane transport, Vesicle docking involved in exocytosis
Q3V0J4	Ankrd53	Ankyrin repeat domain-containing protein 53	Cytoplasm, Spindle	Cell division, Mitotic metaphase chromosome alignment, Positive regulation of microtubule polymerization
Q80U49	Cep170b	Centrosomal protein of 170 kDa protein B	Cytoplasm, Microtubule	-
Q8BH01	Tmco3	Transmembrane and coiled-coil domain-containing protein 3	Membrane	-
Q9DCJ7	Mrps38	Small ribosomal subunit protein mS38	Mitochondrion, Nucleus	Mitochondrial translation, Positive regulation of proteolysis, Protein ubiquitination, Anaphase-promoting complex, Mitochondrion organization
Q8BG34	Ubxn10	UBX domain-containing protein 10	Cilium, Endoplasmic reticulum	Cilium assembly, ERAD pathway
Q80XK6	Atg2b	Autophagy-related protein 2 homolog B	Endoplasmic reticulum, Lipid droplet	Autophagosome assembly, Glycophagy, Reticulophagy, Piecemeal microautophagy of nucleus
Q7M753	Pank2	Pantothenate kinase 2, mitochondrial	Cytosol, Mitochondrion	Aerobic respiration, Coenzyme A biosynthesis, Mitochondrion organization, Regulation of mitochondrial membrane potential, Phosphorylation
Q9D5Y0	Spmip4	Sperm-associated microtubule inner protein 4	Cytoplasm, Centrosome	-
Q9D6A1	Myo1h	Unconventional myosin-Ih	Cytoplasm, Plasma membrane	Actin filament organization, Actin filament-based movement, Endocytosis, Vesicle transport along actin filament
Q8BWL5	Rbms3	RNA-binding motif, single-stranded-interacting protein 3	Cytosol, Nucleus	Positive regulation of translation
P30677	Gna14	Guanine nucleotide-binding protein subunit alpha-14	Cytoplasm	Action potential, G protein-coupled receptor signaling pathway, Adenylate cyclase-modulating G protein-coupled receptor signaling pathway,
P51670	Ccl9	C-C motif chemokine 9	Extracellular space	Inflammatory response, Chemokine-mediated signaling pathway, Neutrophil chemotaxis,
P28649	Cyp19a1	Aromatase	Endoplasmic reticulum, Neuronal cell body	Estrogen biosynthetic process, Androgen metabolic process, Testosterone biosynthetic process
P24383	Wnt7a	Protein Wnt-7a	Cell surface, Extracellular matrix	Canonical Wnt signaling pathway, Cell proliferation, Cell fate commitment, Embryonic limb morphogenesis, Synapse assembly
Q80ZI6	Lrsam1	E3 ubiquitin-protein ligase LRSAM1	Cytoplasm, Membrane	Autophagy, Protein polyubiquitination, Protein catabolic process, Positive regulation of autophagosome assembly,
P70186	Epyc	Epiphycan	Extracellular matrix	Articular cartilage development, Bone development, Sensory perception of sound
O35459	Ech1	Delta(3,5)-Delta(2,4)-dienoyl-CoA isomerase, mitochondrial	Mitochondrion, Peroxisome	Fatty acid beta-oxidation

Uniprot ID	Gene	Short name	Subcellular location	Gene ontology
Q9EQ61	Pes1	Pescadillo homolog	Nucleus, Nucleolus	Ribosome biogenesis, Rrna processing, Regulation of cell cycle, Nucleolus organization, Fibroblast proliferation
Q921M7	Cyrib	CYFIP-related Rac1 interactor B	Cilium, Membrane, Mitochondrion	Cellular response to molecule of bacterial origin, Negative regulation of actin filament polymerization
P97321	Fap	Prolyl endopeptidase FAP	Plasma membrane, Extracellular space, Apical part of cell	Angiogenesis, Cell adhesion, Endothelial cell migration, Negative regulation of cell proliferation involved in contact inhibition, Proteolysis
Q3T9Z9	Zup1	Zinc finger-containing ubiquitin peptidase 1	Cytosol, Nucleus	-
Q5SXA9	Wwc1	Protein KIBRA	Cytoplasm, Cytosol, Nucleus	Cell migration, Negative regulation of cell population proliferation, Negative regulation of Hippo signaling
Q810P3	Cdkn3	Cyclin-dependent kinase inhibitor 3	Cytoplasm, Cytosol, Nucleus	Cell cycle, Dephosphorylation, DNA damage response, Regulation of cell cycle, (No fifth GO term)
Q3V079	Bbof1	Basal body-orientation factor 1	Axoneme, Ciliary basal body, Sperm flagellum	Flagellated sperm motility, Motile cilium assembly, Sperm axoneme assembly
P23492	Pnp	Purine nucleoside phosphorylase	Cytoplasm, Cytosol, Extracellular region	Adenosine catabolic process, AMP catabolic process, B cell differentiation, DNA repair
Q6P8X6	Usp50	Ubiquitin carboxyl-terminal hydrolase 50	Cytoplasm, Cytosol, Nucleus	Cell cycle, Endosome organization, Positive regulation of interleukin-1 beta production, Proteolysis, Regulation of cell cycle G2/M phase transition
Q99J72	Apobec3	DNA dC->dU-editing enzyme APOBEC-3	Cytoplasm, Nucleus	Defense response to virus, DNA cytosine deamination, Innate immune response, Regulation of viral life cycle, Retrotransposon silencing
P83510	Tnik	Traf2 and NCK-interacting protein kinase	Cytoplasm, Cytoskeleton, Nucleus	Actin cytoskeleton organization, MAPK cascade, Protein phosphorylation, Regulation of dendrite morphogenesis, Wnt signaling pathway
Q61751	Znf354a	Zinc finger protein 354A	Nucleus	Negative regulation of transcription by RNA polymerase II, Nucleolus organization, Regulation of transcription by RNA polymerase II
Q9Z2Q2	Knop1	Lysine-rich nucleolar protein 1	Nucleolus	(No processes listed)
O88559	Men1	Menin	Chromatin, Cytoplasm, Nucleus	Cell division, Chromatin remodeling, Fibroblast proliferation, Negative regulation of cell cycle, Positive regulation of gene expression
Q7TS75	Amer1	APC membrane recruitment protein 1	Cytoplasm, Nucleus, Plasma membrane	Bone development, Negative regulation of canonical Wnt signaling pathway, Positive regulation of canonical Wnt signaling pathway
Q8VBY2	Camkk1	Calcium/calmodulin-dependent protein kinase kinase 1	Cytoplasm, Cytosol, Nucleus	Intracellular signal transduction, Phosphorylation, (No other GO terms listed)
Q8C827	Zfp62	Zinc finger protein 62 (Zfp-62) (ZT3)	Nucleus	Regulation of transcription by RNA polymerase II, Gene expression, DNA binding

Uniprot ID	Gene	Short name	Subcellular location	Gene ontology
Q9WV02	Rbmx	RNA-binding motif protein, X chromosome (Heterogeneous nuclear ribonucleoprotein G)	Nucleus, Postsynapse	MRNA splice site recognition, Positive regulation of transcription, Positive regulation of mRNA splicing, Regulation of postsynapse organization, RNA splicing
E9Q7R9	Cfap43	Cilia- and flagella-associated protein 43	Cilium, Extracellular region	Cilium assembly, Brain development, Cerebrospinal fluid circulation, Motile cilium assembly, Flagellated sperm motility
Q9QYY8	Spastin	Spastin	Cytoplasm, Nucleus, Axon	Microtubule severing, Axonal transport, Cytokinesis, Membrane fission, Microtubule bundle formation
Q8VCQ6	Sgms1	Phosphatidylcholine:ceramide cholinephosphotransferase 1	Endoplasmic reticulum, Golgi	Ceramide biosynthetic process, Sphingomyelin biosynthetic process, Positive regulation of gene expression, Apoptotic process, Phosphorylation
P50431	Shmt1	Serine hydroxymethyltransferase, cytosolic	Cytoplasm, Mitochondrion, Nucleus	One-carbon metabolic process, Glycine biosynthetic process, Folic acid metabolic process, DTMP biosynthetic process
Q99KK7	Dpp3	Dipeptidyl peptidase 3	Cytoplasm	Protein catabolic process, Proteolysis, Peptidase activity, Protein processing, Cellular protein metabolic process
Q9DBD2	Marchf8	E3 ubiquitin-protein ligase MARCHF8	Cytoplasm, Endosome, Lysosome	Protein ubiquitination, Immune response, Antigen processing, Negative regulation of MHC class II biosynthetic process, Adaptive immune response
B7ZCC9	Adgrg4	Adhesion G-protein coupled receptor G4	Membrane	Cell surface receptor signaling, Neurogenesis, Adenylate cyclase-activating G protein-coupled receptor signaling pathway, Signal transduction, Cell communication
P28665	Mug1	Murinoglobulin-1	Extracellular space	Embryo implantation, Immune response, Protein binding, Developmental process, Cell communication
P49698	Hnf4a	Hepatocyte nuclear factor 4-alpha	Nucleus, Chromatin	Regulation of transcription, Metabolic process, Glucose homeostasis, Lipid metabolic process, Positive regulation of gene expression
Q3UWY1	Oog3	Oogenesin-3	Cytoplasm	Negative regulation of apoptotic process, Positive regulation of cell proliferation, Negative regulation of cell differentiation, Negative regulation of transcription, Cell growth
Q4VC33	Maea	E3 ubiquitin-protein transferase MAEA	Cytoplasm, Nucleus, Plasma membrane	Cell adhesion, Cell cycle, Proteasome-mediated ubiquitin-dependent protein catabolic process, Erythrocyte development, Cytoskeleton organization
Q9Z1S8	Gab2	GRB2-associated-binding protein 2	Cytoplasm, Plasma membrane	Signal transduction, Cell migration, Positive regulation of cell proliferation, Integrin-mediated signaling pathway, Osteoclast differentiation

Uniprot ID	Gene	Short name	Subcellular location	Gene ontology
Q8VHY0	Cspg4	Chondroitin sulfate proteoglycan 4	Plasma membrane, Nucleoplasm	Angiogenesis, Glial cell migration, Negative regulation of neuron projection development, Positive regulation of MAPK cascade, Tissue remodeling
Q52KE7	Cyclin-L1	Cyclin-L1	Nucleus, Cytoplasm	Regulation of apoptotic process, Regulation of cell cycle, Regulation of centrosome cycle
Q8R1F5	Hyi	Putative hydroxypyruvate isomerase	Cytoplasm	Glyoxylate metabolic process
Q3UQV5	Kbtbd8	Kelch repeat and BTB domain-containing protein 8	Golgi apparatus, Spindle	Neural crest cell development, Neural crest formation, Protein monoubiquitination, Regulation of translation
Q3USL1	Klhdc9	Kelch domain-containing protein 9	-	-
Q80TC6	Zswim5	Zinc finger SWIM domain-containing protein 5	-	-
Q3U186	Rars2	Probable arginine--tRNA ligase, mitochondrial	Mitochondrion	Arginyl-tRNA aminoacylation, Mitochondrial translation
O08675	F2rl2	Proteinase-activated receptor 3	Plasma membrane	Blood coagulation, G protein-coupled receptor signaling pathway, Ligand-gated ion channel signaling pathway, Positive regulation of insulin secretion
Q8VE22	Mrps23	Small ribosomal subunit protein mS23, mitochondrial	Mitochondrion	Mitochondrial translation
P97820	Map4k4	Mitogen-activated protein kinase kinase kinase kinase 4	Cytoplasm, Focal adhesion	MAPK cascade, Negative regulation of insulin secretion, Negative regulation of neuron projection development, Positive regulation of GTPase activity
Q9DBD0	Inhca	Inhibitor of carbonic anhydrase	Early endosome, Plasma membrane, Extracellular space	Antibacterial humoral response, Iron ion transport
Q7TNL3	Stk40	Serine/threonine-protein kinase 40	Cytoplasm, Nucleus	Glycogen metabolic process, Lung alveolus development, Negative regulation of apoptotic process, Phosphorylation, Regulation of MAPK cascade
Q0VEE6	Znf800	Zinc finger protein 800	Nucleus	Acinar cell differentiation, Endocrine pancreas development
A6H5Y3	Mtr	Methionine synthase	Cytoplasm	Axon regeneration, Cellular response to nitric oxide, Cobalamin metabolic process, Homocysteine metabolic process, Methionine biosynthetic process
Q9Z1D9	Znf394	Zinc finger protein 394	Nucleus	Regulation of transcription by RNA polymerase II
O35551	Rabep1	Rab GTPase-binding effector protein 1	Early endosome	Apoptotic process, Endocytosis, Golgi to plasma membrane transport, Protein transport, Vesicle-mediated transport
Q8C0I1	Agps	Alkyldihydroxyacetonephosphate synthase, peroxisomal	Mitochondrion, Peroxisome	Ether lipid biosynthetic process, Lipid biosynthetic process
O08755	Onecut1	Hepatocyte nuclear factor 6	Nucleus	Anatomical structure morphogenesis, B cell differentiation, Cell migration, Cilium assembly, Pancreas development

Uniprot ID	Gene	Short name	Subcellular location	Gene ontology
A2RSJ4	Bltp3b	Bridge-like lipid transfer protein family member 3B	Cytoplasm, Early endosome	Early endosome to Golgi transport, Intermembrane lipid transfer
Q61088	Fzd4	Frizzled-4	Plasma membrane, Cell surface	Angiogenesis, Blood vessel development, Canonical Wnt signaling pathway, Cell population proliferation, Endothelial cell differentiation
Q9QXL8	Nme7	Nucleoside diphosphate kinase homolog 7	Nucleus, Centrosome	Brain development, Cellular response to leukemia inhibitory factor, CTP biosynthetic process
Q61809	Lrrn1	Leucine-rich repeat neuronal protein 1	Plasma membrane, extracellular space	Positive regulation of synapse assembly
Q9EPN1	Nbea	Neurobeachin	Cytosol, postsynaptic membrane, nucleus	Synapse organization, Protein localization, Regulation of neurotransmitter receptor localization, Protein targeting, Post-Golgi vesicle-mediated transport
O08650	Has3	Hyaluronan synthase 3	Cytoplasm, Golgi apparatus, plasma membrane	Hyaluronan biosynthetic process, Extracellular matrix assembly, Positive regulation of transcription, Extracellular polysaccharide biosynthetic process, Positive regulation of hyaluronan cable assembly
D0VYS2	Hsf3	Heat shock factor protein 3	Nucleus, cytoplasm	Cellular response to heat, Positive regulation of transcription by RNA polymerase II, Regulation of transcription by RNA polymerase II
Q9CUN6	Smurf1	E3 ubiquitin-protein ligase SMURF1	Cytoplasm, axon, plasma membrane	Protein ubiquitination, Negative regulation of BMP signaling pathway, Proteasome-mediated protein catabolic process, Positive regulation of axon extension, Parkin-mediated stimulation of mitophagy
P70424	Erbb2	Receptor tyrosine-protein kinase erbB-2	Plasma membrane, cytoplasm, nucleus	Neurogenesis, Nervous system development, Positive regulation of cell growth, Myelination, ERBB2-EGFR signaling pathway
Q9D3N2	Clxn	Calaxin	Cilium, axoneme	Cilium movement, Regulation of cilium movement, Outer dynein arm assembly, Cell chemotaxis, Regulation of flagellated sperm motility
Q9JLI6	Scly	Selenocysteine lyase	Cytosol	Selenium compound metabolic process, Lipid metabolic process, Response to insulin, Selenocysteine catabolic process, Negative regulation of cellular response to oxidative stress
Q91YN9	Bag2	BAG family molecular chaperone regulator 2	Cytoplasm, axon, dendrite	Protein stabilization, Positive regulation of protein processing, Protein metabolic process
Q810M5	Zdhhc19	Palmitoyltransferase ZDHHC19	Endoplasmic reticulum, Golgi apparatus	Protein targeting to membrane, Peptidyl-L-cysteine S-palmitoylation, Positive regulation of aggrephagy

Uniprot ID	Gene	Short name	Subcellular location	Gene ontology
O70318	Epb41l2	Band 4.1-like protein 2	Cytoskeleton, plasma membrane	Actin cytoskeleton organization, Cell division, Regulation of cell shape, Cell cycle, Cortical actin cytoskeleton organization
A2AWP8	Arhgef10l	Rho guanine nucleotide exchange factor 10-like protein	Cytosol	Actin cytoskeleton organization, Positive regulation of stress fiber assembly, SREBP signaling pathway
Q8BWY3	Etf1	Eukaryotic peptide chain release factor subunit 1	Cytoplasm, ribosome	Translational termination, Regulation of translational termination, Nonsense-mediated decay, Cytoplasmic translational termination
O89026	Robo1	Roundabout homolog 1	Axon, plasma membrane, dendrite	Axon guidance, Heart development, Synapse organization, Negative regulation of gene expression, Regulation of dendrite morphogenesis
Q08761	Pros1	Vitamin K-dependent protein S	Extracellular space	Blood coagulation, Negative regulation of coagulation, Fibrinolysis, Positive regulation of phagocytosis, Generation of neurons
Q9D665	Sdr42e1	Short-chain dehydrogenase/reductase family 42E member 1	Membrane	Steroid biosynthetic process
Q80YR4	Znf598	E3 ubiquitin-protein ligase ZNF598	Cytosol	Protein ubiquitination, Ribosome-associated ubiquitin-dependent protein catabolic process, Rescue of stalled ribosome, Protein monoubiquitination, Regulation of translation
Q61666	Hira	Protein HIRA	Nucleus, chromatin	Chromatin remodeling, Nucleosome assembly, DNA-templated transcription, Regulation of DNA-templated transcription, Muscle cell differentiation
P61079	Ube2d3	Ubiquitin-conjugating enzyme E2 D3	Nucleus, plasma membrane, endosome	Protein polyubiquitination, DNA repair, Protein K48-linked ubiquitination, Apoptotic process, Ubiquitin-dependent protein catabolic process
Q5SSE9	Abca13	ATP-binding cassette sub-family A member 13	Cytoplasmic vesicle, intracellular organelle	Lipid transport, Ceramide transport, Positive regulation of cholesterol transport, Positive regulation of synaptic vesicle endocytosis
Q8K2G4	Bbs7	Bardet-Biedl syndrome 7 protein homolog	Cilia, nucleus, membrane	Cilium assembly, Protein transport, Brain development, Eye development, Limb development
P04945	Nq2-6.1	Ig kappa chain V-VI region NQ2-6.1	Extracellular space	Adaptive immune response, Immune response
B2RY83	Hpse2	Inactive heparanase-2	Membrane, extracellular space	Extracellular matrix organization, Cell population proliferation, Positive regulation of cell population proliferation
Q810B7	Slitrk5	SLIT and NTRK-like protein 5	Synapse, membrane	Axonogenesis, Synapse assembly, Dendrite morphogenesis, Chemical synaptic transmission, Adult behavior

Uniprot ID	Gene	Short name	Subcellular location	Gene ontology
Q9D8C4	Ifi35	Interferon-induced 35 kDa protein homolog	Cytoplasm, nucleus, membrane	Innate immune response, Macrophage activation, Inflammatory response, Non-canonical NF-kappaB signaling, Toll-like receptor 4 signaling
Q03517	Scg2	Secretogranin-2	Secretory granule, extracellular space	Angiogenesis, MAPK cascade, Positive chemotaxis, Endothelial cell proliferation, Signal transduction
Q80UZ0	Fgd5	FYVE, RhoGEF and PH domain-containing protein 5	Cytoplasm, Golgi apparatus	—
Q8BJR6	Prss27	Serine protease 27	Plasma membrane, extracellular region	Proteolysis
Q9R118	Htra1	Serine protease HTRA1	Plasma membrane, extracellular region, cytosol	Proteolysis, Apoptotic process, Placenta development, BMP signaling regulation, TGF-beta signaling regulation
Q8VC70	Rbms2	RNA-binding motif, single-stranded-interacting protein 2	Nucleus, cytosol	—
Q9JJW6	Alyref2	Aly/REF export factor 2	Nucleus, Cytoplasm	mRNA export from nucleus, mRNA processing, mRNA transport, RNA splicing, RNA binding
Q80TS7	Ddn	Dendrin	Plasma membrane, Nucleus	Positive regulation of transcription by RNA polymerase II, Synapse assembly, Dendrite morphogenesis
Q8C0C0	Zhx2	Zinc fingers and homeoboxes protein 2	Nucleus, Cytosol	Negative regulation of DNA-templated transcription, Neuron differentiation, Regulation of transcription by RNA polymerase II, mRNA catabolic process, Somatic stem cell population maintenance
Q8BTV2	Cpsf7	Cleavage and polyadenylation specificity factor subunit 7	Nucleus, Cytoplasm	mRNA alternative polyadenylation, Messenger ribonucleoprotein complex assembly, mRNA processing, Protein heterotetramerization, RNA binding
Q8R3L2	Tcf25	Ribosome quality control complex subunit TCF25	Nucleus, Cytosol	Negative regulation of transcription by RNA polymerase II, Protein ubiquitination, Ribosome rescue
Q61362	Chi3l1	Chitinase-3-like protein 1	Cytoplasm, Extracellular space	Inflammatory response, Apoptotic process, Activation of NF-kappaB-inducing kinase activity, Positive regulation of angiogenesis, Cellular response to tumor necrosis factor
Q5NBU8	Xaf1	XIAP-associated factor 1	Nucleus, Mitochondrion	Apoptotic process, Negative regulation of protein-containing complex assembly, Regulation of apoptosis
Q505K2	Fhip1a	FHF complex subunit HOOK-interacting protein 1A	Cytoplasm	Protein localization to perinuclear region of cytoplasm, Cytoskeleton organization, Vesicle-mediated transport, Endosomal trafficking, Organelle organization
O08795	Prkcsh	Glucosidase 2 subunit beta	Endoplasmic reticulum	Embryonic development, Liver development, N-glycan processing, Protein folding,
Q7TT15	Galnt17	Polypeptide N-acetylgalactosaminyltransferase 17	Golgi apparatus	Protein O-linked glycosylation, Glycoprotein biosynthetic process, Polysaccharide biosynthetic process

Uniprot ID	Gene	Short name	Subcellular location	Gene ontology
Q07417	Acads	Short-chain specific acyl-CoA dehydrogenase, mitochondrial	Mitochondrion, Centrosome	Fatty acid beta-oxidation, Butyrate catabolic process, Cellular respiration, Lipid metabolic process, Energy derivation by oxidation of organic compounds
Q8BL97	Srsf7	Serine/arginine-rich splicing factor 7	Nucleus, Cytoplasm	mRNA splicing via spliceosome, Negative regulation of mRNA splicing, mRNA transport, Regulation of mRNA processing, RNA binding
Q8VCS3	Fam20b	Glycosaminoglycan xylosylkinase	Golgi apparatus, Extracellular matrix	Phosphorylation, Proteoglycan biosynthetic process, Glycosaminoglycan biosynthetic process, Protein phosphorylation, Cellular protein modification process
Q8CID0	Kat14	Cysteine-rich protein 2-binding protein	Nucleus, Cytoplasm	Regulation of cell cycle, Regulation of transcription by RNA polymerase II, Regulation of cell division, G2/M transition of mitotic cell cycle
Q62230	Siglec1	Sialoadhesin	Plasma membrane, Endosome	Cell adhesion, Clathrin-dependent endocytosis, Positive regulation of extrinsic apoptotic signaling pathway,
Q99LU0	Chmp1b1	Charged multivesicular body protein 1B1	Cytosol, Nucleus, Plasma membrane	Autophagosome maturation, Autophagy, Endosome transport, Membrane fission, Protein transport
Q3V3Q7	Pacs2	Phosphofurin acidic cluster sorting protein 2	Endoplasmic reticulum, Mitochondrion	Apoptotic process, Autophagosome assembly, Protein localization to phagophore assembly site, Protein localization to plasma membrane
O35681	Syt3	Synaptotagmin-3	Plasma membrane, Vesicles	Calcium-ion regulated exocytosis, Cell differentiation, Regulation of vesicle fusion, Regulation of calcium-dependent exocytosis, Response to calcium ion
P15919	Rag1	V(D)J recombination-activating protein 1	Nucleus	Adaptive immune response, B cell differentiation, DNA recombination, T cell differentiation, Chromatin organization
P43883	Plin2	Perilipin-2	Cytoplasm, Lipid droplet	Lipid storage, Cellular response to glucose starvation, Lipid droplet disassembly, Fatty acid transport, Response to organic cyclic compound
Q3U8K7	Kmt5b	Histone-lysine N-methyltransferase KMT5B	Nucleus, Chromosome	DNA repair, Methylation, Muscle organ development, Regulation of double-strand break repair, Positive regulation of isotype switching
P58064	Mrps6	Small ribosomal subunit protein bS6m	Mitochondrion	Mitochondrial translation
P52963	Epb41l4a	Band 4.1-like protein 4A	Cytoplasm, Cytoskeleton	Actomyosin structure organization
P70290	Mpp1	55 kDa erythrocyte membrane protein (p55)	Plasma membrane, Cytoskeleton	Regulation of neutrophil chemotaxis
O88572	Lrp6	Low-density lipoprotein receptor-related protein 6	Plasma membrane, Endoplasmic reticulum	Canonical Wnt signaling pathway, Bone remodeling, Embryonic development, Cell migration, Neural tube development

Uniprot ID	Gene	Short name	Subcellular location	Gene ontology
P06683	C9	Complement component C9	Extracellular space, Plasma membrane	Complement activation, Cell killing, Immune response
Q8CCH7	Zfpm2	Zinc finger protein ZFPM2	Nucleus, Cytoplasm	Cardiac muscle development, Cell differentiation, Regulation of transcription, Embryonic development, Vasculogenesis
Q8BGV8	Mief1	Mitochondrial dynamics protein MID51	Mitochondrion	Mitochondrial fission, Response to hypoxia, Protein targeting to membrane
A6X8Z5	Arhgap31	Rho GTPase-activating protein 31	Focal adhesion, Cytoskeleton	Small GTPase-mediated signal transduction
Q9D554	Sf3a3	Splicing factor 3A subunit 3	Nucleus, Nuclear speckles	mRNA splicing, Spliceosome assembly
Q6GQX6	Anks6	Ankyrin repeat and SAM domain-containing protein 6 (SamCystin)	Cytoplasm, Nucleus	Determination of left/right symmetry, Heart development, In utero embryonic development, Innate immune response, Kidney development
Q3UX10	Tubal3	Tubulin alpha chain-like 3	Cytoplasm, Microtubule	Microtubule cytoskeleton organization, Mitotic cell cycle
Q14BU0	Ucma	Unique cartilage matrix-associated protein	Cytoplasm, Extracellular matrix, Golgi apparatus	Embryonic skeletal system development, Negative regulation of osteoblast differentiation, Negative regulation of SMAD protein signal transduction, Osteoblast differentiation
O88855	Ltb4r1	Leukotriene B4 receptor 1	Plasma membrane	Inflammatory response, Neuropeptide signaling pathway, Phospholipase C-activating G protein-coupled receptor signaling pathway
Q8BHR2	Enox1	Ecto-NOX disulfide-thiol exchanger 1	Plasma membrane, Extracellular region	Ultradian rhythm
Q8BX10	Pgam5	Serine/threonine-protein phosphatase PGAM5, mitochondrial	Mitochondrion	Dephosphorylation, Necroptotic process, Negative regulation of cold-induced thermogenesis, Positive regulation of mitochondrial fission
Q9CRC0	Vkorc1	Vitamin K epoxide reductase complex subunit 1	Endoplasmic reticulum	Blood coagulation, Bone development, Peptidyl-glutamic acid carboxylation, Positive regulation of coagulation, Vitamin K metabolic process
Q4VBE4	Egflam	Pikachurin (EGF-like, fibronectin type-III and laminin G-like domain-containing protein)	Basement membrane, Extracellular matrix	Extracellular matrix organization, Positive regulation of cell-substrate adhesion
Q8CHX7	Rftn2	Raftlin-2	Plasma membrane	DsRNA transport, Response to exogenous dsRNA
Q059U7	Intu	Protein inturned (Inturned planar cell polarity effector homolog)	Cell surface, Centriole, Cilium	Cell division, Cilium assembly, Embryonic digit morphogenesis, Establishment of planar polarity, Hair follicle morphogenesis
Q80XH2	Impg2	Interphotoreceptor matrix proteoglycan 2	Extracellular matrix, Cell projection	Extracellular matrix organization, Protein localization, Retina morphogenesis, Visual perception
Q8VDI1	Epsti1	Epithelial-stromal interaction protein 1	-	-

Uniprot ID	Gene	Short name	Subcellular location	Gene ontology
Q69ZT1	Fan1	Fanconi-associated nuclease 1	Cytosol, Nucleus	DNA repair, Double-strand break repair via homologous recombination, Interstrand cross-link repair, Nucleotide-excision repair
P35922	Fmr1	Fragile X messenger ribonucleoprotein 1	Axon, Cytoplasm	Animal organ development, Central nervous system development, DNA repair, mRNA processing, Synaptic transmission
Q61473	Sox17	Transcription factor SOX-17	Nucleus, Nucleoplasm	Angiogenesis, Heart development, Endoderm development, Regulation of transcription by RNA polymerase II, Cell differentiation
Q3TPX4	Exoc5	Exocyst complex component 5	Cytosol, Golgi apparatus	Exocytosis, Protein transport, Vesicle docking, Membrane fission, Cytokinesis
Q64016	Defa17	Alpha-defensin 17	Extracellular space	Antibacterial humoral response, Innate immune response, Defense response to Gram-negative bacterium, Antimicrobial peptide response, Defense response to Gram-positive bacterium
Q5GH66	Xkr5	XK-related protein 5	Plasma membrane	Apoptotic process, Engulfment of apoptotic cell, Phosphatidylserine exposure on apoptotic cell surface, - , -
Q8BQJ6	Trmt2b	tRNA (uracil-5-)-methyltransferase homolog B	Mitochondrion	tRNA processing
P97784	Cry1	Cryptochrome-1	Nucleus, Cytoplasm, Mitochondrion	Circadian rhythm, Regulation of gene expression, Glucose homeostasis, Signal transduction, Response to light stimulus
Q6X7S9	Eid2	EP300-interacting inhibitor of differentiation 2	Nucleus, Nucleoplasm	Cell differentiation, Negative regulation of transcription, Muscle organ development, Regulation of cell proliferation, Negative regulation of TGF-beta receptor signaling
Q61097	Ksr1	Kinase suppressor of Ras 1	Cytoplasm, Endoplasmic reticulum	MAPK cascade regulation, Phosphorylation, Ras protein signal transduction, Regulation of cell proliferation, Regulation of MAP kinase activity
A9Z1V5	Vwa5b1	von Willebrand factor A domain-containing protein 5B1	Extracellular region	-
Q3URK3	Tet1	Methylcytosine dioxygenase TET1	Nucleus, Chromosome	DNA demethylation, Chromatin remodeling, Stem cell population maintenance, Gene expression regulation, Cell differentiation
Q9QWG7	Sult1b1	Sulfotransferase 1B1	Cytoplasm, Cytosol	Xenobiotic metabolic process, Sulfation, Ethanol catabolic process, Epithelial cell differentiation, Thyroid hormone metabolic process
Q3TTA7	Cblb	E3 ubiquitin-protein ligase CBL-B	Cytosol, Plasma membrane	Immune response, Protein ubiquitination, Signal transduction, Regulation of T cell receptor signaling, T cell activation
Q91WN1	Dnajc9	DnaJ homolog subfamily C member 9	Cytoplasm, Nucleus, Plasma membrane	Nucleosome assembly, Protein folding

Uniprot ID	Gene	Short name	Subcellular location	Gene ontology
P27577	Ets1	Protein C-ets-1	Cytoplasm, Nucleus	Cell differentiation, Immune system process, Regulation of transcription, Angiogenesis, Positive regulation of cell migration
Q9EPX2	Papln	Papilin	Extracellular region	Extracellular matrix organization; Proteolysis
Q6P6I6	Polr2m	DNA-directed RNA polymerase II subunit GRINL1A	Nucleus; Neuronal cell body	Transcription elongation by RNA polymerase II; Intracellular signal transduction; Maintenance of ER location
Q99J87	Dhx58	ATP-dependent RNA helicase DHX58	Cytoplasm	Antiviral innate immune response; Cytoplasmic pattern recognition receptor signaling
Q8BH00	Aldh8a1	2-aminomuconic semialdehyde dehydrogenase	Cytoplasm	9-cis-retinoic acid biosynthetic process; Retinal metabolic process; L-kynurenine catabolic process; Polyamine catabolic process
Q99LB2	Dhrs4	Dehydrogenase/reductase SDR family member 4	Mitochondrion; Peroxisome	Cellular ketone metabolic process; Positive regulation of reactive oxygen species metabolic process; Retinal metabolic process; Steroid metabolic process
P37242	Thrb	Thyroid hormone receptor beta	Nucleus	Regulation of transcription by RNA polymerase II; Positive regulation of cell population proliferation; Cell differentiation
O88451	Rdh7	Retinol dehydrogenase 7	Endoplasmic reticulum	Retinol metabolic process; Steroid metabolic process
Q5SUA5	Myo1g	Unconventional myosin-Ig	Cytoplasm; Plasma membrane	Actin filament organization; Phagocytosis; Cell-substrate adhesion; Exocytosis; T cell mediated immunity
P49222	Epb42	Protein 4.2	Plasma membrane; Cortical cytoskeleton	Cell morphogenesis; Erythrocyte maturation; Hemoglobin metabolic process; Monoatomic ion homeostasis; Regulation of cell shape
Q9CRB9	Mic19	MICOS complex subunit Mic19	Mitochondrion; Nucleus	Cristae formation; Inner mitochondrial membrane organization; Mitochondrial fusion
Q5Y5T5	Zdhhc8	Palmitoyltransferase ZDHHC8	Golgi apparatus; Mitochondrion	Peptidyl-L-cysteine S-palmitoylation; Positive regulation of viral process; Locomotory behavior
Q5SSI6	Utp18	U3 small nucleolar RNA-associated protein 18 homolog	Nucleus	Ribosomal small subunit biogenesis; rRNA processing
Q8BPI1	Dnaaf2	Protein kintoun	Cytoplasm; Ciliary basal body	Axonemal dynein complex assembly; Cilium-dependent cell motility; Establishment of left/right asymmetry; Inner dynein arm assembly; Outer dynein arm assembly
Q9D9S1	—	UPF0193 protein EVG1 homolog	—	—
Q08288	Lyar	Cell growth-regulating nucleolar protein	Nucleus; Cytoplasm	Erythrocyte development; Innate immune response; Negative regulation of innate immune response; rRNA processing; Positive regulation of phagocytosis
Q7TN79	Akap7	A-kinase anchor protein 7 isoform gamma	Cytoplasm; Apical plasma membrane	Modulation of chemical synaptic transmission; Protein localization; Regulation of protein kinase A signaling

Uniprot ID	Gene	Short name	Subcellular location	Gene ontology
Q3THK3	Gtf2f1	General transcription factor IIF subunit 1	Nucleus; Cell junction	Positive regulation of transcription by RNA polymerase II; Transcription elongation by RNA polymerase II
P08553	Nefm	Neurofilament medium polypeptide	Axon; Cytoplasm	Regulation of axon diameter; Intermediate filament cytoskeleton organization; Axo-dendritic transport; Microtubule cytoskeleton organization; Neurofilament cytoskeleton organization
Q5SS90	—	Uncharacterized protein C7orf57 homolog	—	—
Q6PGF3	Med16	Mediator of RNA polymerase II transcription subunit 16	Nucleus; Nucleoplasm	Positive regulation of transcription initiation by RNA polymerase II; Positive regulation of transcription elongation by RNA polymerase II
Q9Z188	Dyrk1b	Dual specificity tyrosine-phosphorylation-regulated kinase 1B	Nucleus; Nucleolus	DNA repair; Phosphorylation; Positive regulation of DNA-templated transcription; Adipose tissue development; Myoblast fusion
Q9JIP7	Slc15a1	Solute carrier family 15 member 1	Apical plasma membrane; Plasma membrane	Oligopeptide transport; Dipeptide import across plasma membrane; Protein transport; Negative regulation of amino acid transport
Q8K0Z7	Taco1	Translational activator of cytochrome c oxidase 1	Mitochondrion; Cytosol	Mitochondrial cytochrome c oxidase assembly; Regulation of mitochondrial translation; Motor learning
Q9ESJ4	Nckipsd	NCK-interacting protein with SH3 domain	Cytoplasm	Endocytosis; Actin cortical patch assembly; Positive regulation of neuron projection development; Actin cortical patch localization
Q8CHY7	Ttc23	Tetratricopeptide repeat protein 23	Cilium	Positive regulation of smoothened signaling pathway
Q99JF8	Psip1	PC4 and SFRS1-interacting protein	Nucleus; Euchromatin	Positive regulation of transcription by RNA polymerase II; Chromatin remodeling; Response to heat; Response to oxidative stress; mRNA 5'-splice site recognition
Q76LL6	Fhod3	FH1/FH2 domain-containing protein 3	Cytoplasm; Cytoskeleton	Cardiac myofibril assembly; Cortical actin cytoskeleton organization; Sarcomere organization; Negative regulation of actin filament polymerization
Q9JLJ2	Aldh9a1	4-trimethylaminobutyraldehyde dehydrogenase	Cytoplasm; Cytosol; Mitochondrion	Carnitine biosynthetic process; Carnitine metabolic process; Cellular aldehyde metabolic process
Q00623	Apoa1	Apolipoprotein A-I	Extracellular region; Cell surface	Cholesterol biosynthetic process; Cholesterol homeostasis; Reverse cholesterol transport; Positive regulation of cholesterol efflux; Cholesterol transport
Q7TMF3	Ndufa12	NADH dehydrogenase [ubiquinone] 1 alpha subcomplex subunit 12	Mitochondrial inner membrane; Mitochondrion	Aerobic respiration; Mitochondrial respiratory chain complex I assembly
Q03141	Mark3	MAP/microtubule affinity-regulating kinase 3	Cytoplasm, Plasma membrane, Dendrite	Intracellular signal transduction, Microtubule cytoskeleton organization, Negative regulation of Hippo signaling

Uniprot ID	Gene	Short name	Subcellular location	Gene ontology
Q9JII1	Inpp5e	Phosphatidylinositol polyphosphate 5-phosphatase type IV	Plasma membrane, Golgi apparatus, Nucleus	Negative regulation of PI3K/AKT signal transduction, Phosphatidylinositol dephosphorylation, Negative regulation of translation
O08999	Ltbp2	Latent-transforming growth factor beta-binding protein 2	Golgi apparatus, Extracellular matrix, Endosome	Golgi to vacuole transport, Protein targeting to vacuole, Supramolecular fiber organization
B1AUR6	Mms22l	Protein MMS22-like	Cytosol, Nucleus	Chromatin organization, Double-strand break repair via homologous recombination, Protein localization to chromatin, Replication fork processing
Q924S7	Spred2	Sprouty-related, EVH1 domain-containing protein 2	Cytoplasm, Plasma membrane	Negative regulation of MAPK cascade, Negative regulation of epithelial to mesenchymal transition, Negative regulation of ERK1 and ERK2 cascade
Q8CHB8	Ttll5	Tubulin polyglutamylase TTLL5	Cytoplasm, Cilium, Microtubule	Fertilization, Microtubule cytoskeleton organization, Flagellated sperm motility, Sperm axoneme assembly, Spermatogenesis
Q01279	Egfr	Epidermal growth factor receptor	Plasma membrane, Nucleus, Golgi apparatus	Cell surface receptor signaling pathway, Cell population proliferation, Positive regulation of MAPK cascade, Intracellular signal transduction
O35219	Kcnh2	Potassium voltage-gated channel subfamily H member 2	Plasma membrane	Potassium ion transmembrane transport, Regulation of heart rate, Regulation of membrane potential, Membrane repolarization during cardiac muscle cell action potential, Regulation of ventricular cardiac muscle cell membrane repolarization
E9Q6Z5	Aunip	Aurora kinase A- and ninein-interacting protein	Nucleus, Cytoplasm, Centrosome	Double-strand break repair via homologous recombination, Negative regulation of double-strand break repair via nonhomologous end joining, Spindle organization
C0HKC8	Smok3a	Sperm motility kinase 3A	Cytoplasm	Intracellular signal transduction, Microtubule cytoskeleton organization, Phosphorylation,
O70311	Nmt2	Glycylpeptide N-tetradecanoyltransferase 2	Cytoplasm, Golgi apparatus, Plasma membrane	N-terminal peptidyl-glycine N-myristoylation
E9QAT4	Sec16a	Protein transport protein Sec16A	Cytosol, Endoplasmic reticulum membrane, Golgi membrane	Autophagy, COPII vesicle coating, Endoplasmic reticulum organization, Golgi organization, Protein localization to plasma membrane
Q99NE5	Rims1	Regulating synaptic membrane exocytosis protein 1	Plasma membrane, Synapse, Cell projection	Calcium ion-regulated exocytosis of neurotransmitter, Neurotransmitter secretion, Regulation of synaptic plasticity
Q9CQY5	Magt1	Magnesium transporter protein 1	Plasma membrane, Endoplasmic reticulum membrane	Protein N-linked glycosylation, Protein N-linked glycosylation via asparagine

Uniprot ID	Gene	Short name	Subcellular location	Gene ontology
Q61941	Nnt	NAD(P) transhydrogenase, mitochondrial	Mitochondrion, Mitochondrial inner membrane	Cell redox homeostasis, NADPH regeneration, Cellular oxidant detoxification, Positive regulation of mitochondrial membrane potential
Q6P1C1	Ippk	Inositol-pentakisphosphate 2-kinase	Cytoplasm, Nucleus	Inositol phosphate biosynthetic process, Phosphorylation
Q8R5A3	Apbb1ip	Amyloid beta A4 precursor protein-binding family B member 1-interacting protein	Cytosol, Plasma membrane	Positive regulation of cell adhesion, Signal transduction, T cell activation
Q9ESX4	Zcchc17	Zinc finger CCHC domain-containing protein 17	Nucleolus	RNA stabilization
P70270	Rad54l	DNA repair and recombination protein RAD54-like	Nucleus, Nucleoplasm	Chromosome organization, DNA damage response, Double-strand break repair, Lifespan determination, Transcription-coupled nucleotide-excision repair
P18111	Cdx1	Homeobox protein CDX-1	Nucleus	Animal organ morphogenesis, Embryo development, Cell differentiation, Pattern specification
Q704Y3	Trpv1	Transient receptor potential cation channel subfamily V member 1	Plasma membrane, Dendrite	Behavioral response to pain, Calcium ion transport, Cellular response to heat, Positive regulation of apoptotic process
Q64008	Rab34	Ras-related protein Rab-34	Golgi apparatus, Cytoplasm	Cilium assembly, Endocytosis, Lysosomal transport, Phagosome maturation, Positive regulation of protein secretion
P26361	Cftr	Cystic fibrosis transmembrane conductance regulator	Plasma membrane, Cytosol	Chloride transport, Cellular response to cAMP, Lung development, Positive regulation of exocytosis, Water transport
Q9CPT5	Nop16	Nucleolar protein 16	Nucleolus	Ribosomal large subunit biogenesis
Q9CWJ3	Katnbl1	KATNB1-like protein 1	Cytoplasm, Nucleus	Cytoplasmic microtubule organization, Positive regulation of cytoskeleton organization
Q91WM1	Strbp	Spermatid perinuclear RNA-binding protein	Cytoplasm	Cell motility, Spermatid development, Mechanosensory behavior
A2RTF1	Catsperb	Cation channel sperm-associated auxiliary subunit beta	Cilium	Sperm capacitation
Q64127	Trim24	Transcription intermediary factor 1-alpha	Cytoplasm, Nucleus	Calcium ion homeostasis, Cellular response to estrogen stimulus, Epithelial cell proliferation, Regulation of apoptotic process, Protein ubiquitination
Q9CQS6	Gkn2	Gastrokine-2	Extracellular space, Golgi	Regulation of cell population proliferation, Response to bacterium,
O09113	Otp	Homeobox protein orthopedia	Nucleus	Dopaminergic neuron differentiation, Neuroblast proliferation, Forebrain neuron differentiation, Neuroendocrine cell differentiation
Q9CPZ1	Ccdc198	Coiled-coil domain-containing protein 198	Cytoplasm vesicle, Plasma membrane	Energy homeostasis, Metabolic process, Cellular homeostasis, Signal transduction, Protein transport

Uniprot ID	Gene	Short name	Subcellular location	Gene ontology
E9Q7X6	Heg1	Protein HEG homolog 1	Cell surface, Cell junction	Atrial morphogenesis, Cardiac development, Vascular development, Cell growth regulation, Cell-cell adhesion
Q8BZ60	Ston2	Stonin-2	Cytoplasm vesicle, Presynapse	Chemical synaptic transmission, Clathrin-mediated endocytosis, Hematopoietic progenitor differentiation, Protein regulation, Endocytosis
P62915	Gtf2b	General transcription factor IIB	Nucleus, Chromosome	Chromosome organization, Gene expression, Cohesin loading, RNA polymerase II assembly, Transcription initiation
Q91WE2	Nip30	Nuclear interacting protein 30	Nucleus	Negative regulation of proteasomal degradation, Protein binding regulation, Nuclear protein transport, Protein complex assembly, Ubiquitin-dependent protein catabolic process
Q769J6	Adamts13	ADAM metallopeptidase with thrombospondin type 1 motif 13	Extracellular matrix	Blood coagulation, Cellular response to IL-4, Response to lipopolysaccharide, Extracellular matrix organization, Proteolysis
P58750	Pim3	Serine/threonine-protein kinase pim-3	Cytoplasm	Apoptosis regulation, Cell cycle regulation, Insulin secretion regulation, Protein phosphorylation, Signal transduction
P0DI97	Nrxn1	Neurexin-1-beta	Cell surface, Endoplasmic reticulum	Angiogenesis, Calcium-dependent cell adhesion, Cerebellar cell differentiation, Synaptic transmission, Synapse assembly
P70372	Elavl1	ELAV-like protein 1	Cytoplasm, Nucleus	mRNA stabilization, Post-transcriptional regulation, Superoxide generation regulation, Nuclear import, Stress granule formation
Q8CDK3	Iqub	IQ motif and ubiquitin domain-containing protein	Cilium, Extracellular region	Cilium assembly, Epithelial cilium movement, Sperm motility, Smoothened signaling pathway, Cell motility
Q0VBL1	Tigd2	Tigger transposable element-derived protein 2	Nucleus	—
Q62053	Ptger2	Prostaglandin E2 receptor EP2	Plasma membrane	G protein-coupled receptor signaling, Prostaglandin response, Inflammatory response, Cell proliferation regulation, Signal transduction
O88428	Papss2	Bifunctional 3'-phosphoadenosine 5'-phosphosulfate synthase 2	Cytoplasm	3'-Phosphoadenosine 5'-phosphosulfate biosynthesis, Blood coagulation, Bone development, Hormone metabolic process, Sulfate assimilation
Q9QUK6	Tlr4	Toll-like receptor 4	Cell surface, Endosome	Innate immune response activation, NF-kappaB signaling, Astrocyte development, B cell proliferation, Lipopolysaccharide response
Q62073	Map3k7	Mitogen-activated protein kinase kinase kinase 7	Cytoplasm, Nucleus	Angiogenesis, Apoptosis, Immune response, JNK cascade, Neural tube development
Q9EQC0	Chst1	Carbohydrate sulfotransferase 1	Golgi apparatus, Trans-Golgi network	Galactose metabolic process, Inflammatory response, Keratan sulfate biosynthesis, Keratan sulfate metabolism, N-acetylglucosamine metabolism

Uniprot ID	Gene	Short name	Subcellular location	Gene ontology
Q8K2C8	Gpat4	Glycerol-3-phosphate acyltransferase 4	Endoplasmic reticulum	Acyl-CoA metabolic process, CDP-diacylglycerol biosynthesis, Fatty acid metabolism, Lipid biosynthesis, Triglyceride biosynthesis
Q8VCD5	Med17	Mediator of RNA polymerase II transcription subunit 17	Nucleus	Positive regulation of transcription, Transcription elongation, Transcription initiation, Protein ubiquitination, Stem cell maintenance
P20612	Gnat1	Guanine nucleotide-binding protein G(t) subunit alpha-1	Plasma membrane, Cytoplasm	G protein-coupled receptor signaling, Phototransduction, Neural tissue regeneration, Dopamine secretion, Sensory perception
P27641	Xrcc5	X-ray repair cross-complementing protein 5	Nucleus, Cytoplasm	DNA damage response, Double-strand break repair, Innate immune response, Neurogenesis, Telomere maintenance
Q9D1B9	Mrpl28	Large ribosomal subunit protein bL28m	Mitochondrion	Mitochondrial translation, Mitochondrial ribosome, Mitochondrial inner membrane, Mitochondrial large ribosomal subunit, Cytosolic large ribosomal subunit
Q8CI71	Vps50	Syndetin	Recycling endosome, Perinuclear cytoplasm	Endocytic recycling, Protein transport, Membrane organization
P04370	Mbp	Myelin basic protein	Myelin sheath, Cell surface	Myelination, Blood-brain barrier maintenance, MAPK cascade, Axonogenesis regulation, Chemokine production regulation
Q8VDS8	Stx18	Syntaxin-18	Endoplasmic reticulum, Golgi	ER membrane organization, Protein transport, Vesicle-mediated transport, Golgi organization, Organelle assembly
Q8VD63	Tspyl4	Testis-specific Y-encoded-like protein 4	Nucleus	Nucleosome assembly, Chromatin organization, DNA packaging
Q8VDT9	Mrpl50	Large ribosomal subunit protein mL50	Mitochondrion	Mitochondrial translation, Mitochondrial ribosome, Mitochondrial inner membrane
P52431	Pold1	DNA polymerase delta catalytic subunit	Nucleoplasm, Cytosol	DNA replication, DNA repair, Base-excision repair, DNA synthesis, Nucleotide excision repair
Q9D1A0	Prxl2c	Peroxiredoxin-like 2C	Cytoplasm	Positive regulation of ERK cascade, Positive regulation of glycolytic process
P48540	Gdnf	Glial cell line-derived neurotrophic factor	Extracellular region, Golgi apparatus	Nervous system development, Neuron differentiation, Axon guidance, Morphogenesis regulation, Signal transduction
Q9D1H7	Get4	Golgi to ER traffic protein 4 homolog	Cytosol, Nucleus	ER-associated degradation, Protein targeting to ER, Protein stability regulation, Ubiquitin-dependent catabolism, Membrane protein insertion
Q3UHX9	Spout1	Putative methyltransferase C9orf114 homolog	Cytoplasm, Kinetochore, Spindle pole	Cell cycle, Cell division, Maintenance of centrosome location, Methylation, MiRNA processing
Q5SZT7	Nkapl	NKAP-like protein	Nucleus	Cell differentiation, Regulation of gene expression, Spermatogenesis
P36916	Gnl1	Guanine nucleotide-binding protein-like 1	-	DNA damage response

Uniprot ID	Gene	Short name	Subcellular location	Gene ontology
Q5XJY6	Fancb	Fanconi anemia group B protein homolog	Nucleus	Cellular response to camptothecin, Cellular response to xenobiotic stimulus, Interstrand cross-link repair
Q8BQM7	Smco3	Single-pass membrane and coiled-coil domain-containing protein 3	Membrane	-
Q68FF0	Sanbr	SANT and BTB domain regulator of class switch recombination	-	Isotype switching
Q9QZ09	Phtf1	Protein PHTF1	Golgi apparatus, Endoplasmic reticulum	-
Q8BUB6	Ust	Uronyl 2-sulfotransferase	Golgi membrane	Establishment of cell polarity, Regulation of axonogenesis
Q8BZ32	Asxl2	Putative Polycomb group protein ASXL2	Nucleus	Adult heart development, Animal organ morphogenesis, Bone mineralization involved in bone maturation, Embryonic skeletal system morphogenesis, Heart development
Q921N6	Ddx27	Probable ATP-dependent RNA helicase DDX27	Chromosome, Nucleolus	RRNA processing
Q80UJ7	Rab3gap1	Rab3 GTPase-activating protein catalytic subunit	Cytoplasm, Endoplasmic reticulum	Establishment of protein localization to ER membrane, Positive regulation of autophagosome assembly, Positive regulation of gene expression
P50580	Pa2g4	Proliferation-associated protein 2G4	Cytoplasm, Nucleolus, Nucleus	Negative regulation of apoptotic process, Negative regulation of DNA-templated transcription
Q8C4V1	Arhgap24	Rho GTPase-activating protein 24	Cytoplasm, Cytoskeleton	Angiogenesis, Cell differentiation, Negative regulation of Rac protein signal transduction
Q9EQW8	Ndst4	Bifunctional heparan sulfate N-deacetylase/N-sulfotransferase 4	Golgi apparatus, Golgi membrane	Heparan sulfate proteoglycan biosynthetic process, Polysaccharide chain biosynthetic process, Heparin biosynthetic process
Q9ERE3	Sgk3	Serine/threonine-protein kinase Sgk3	Cytoplasmic vesicle, Early endosome, Nucleus	Intracellular signal transduction, Localization, Negative regulation of extrinsic apoptotic signaling pathway, Phosphorylation
P35436	Grin2a	Glutamate receptor ionotropic, NMDA 2A	Cell Surface, Cytoplasm, Dendrite	Synaptic Transmission, Learning, Memory, Neurogenesis, Calcium Ion Transport
Q99L60	Atp6v1c2	V-type proton ATPase subunit C 2	Lysosomal Membrane, Vacuole	Positive Regulation of Wnt Signaling
Q9WU78	Pdcd6ip	Programmed cell death 6-interacting protein	Cytosol, Endosome, Exosome	Apoptotic Process, Protein Transport, Macroautophagy, Cytokinesis, Cell Polarity
Q8BM55	Tmem214	Transmembrane protein 214	Cytoplasm, Endoplasmic Reticulum, Golgi Apparatus	Apoptotic Process
Q68ED3	Tent4b	Terminal nucleotidyltransferase 4B	Cytoplasm, Nucleolus	RNA 3'-end Processing, Cell Cycle, mRNA Catabolic Process, rRNA Processing, miRNA Catabolic Process
Q9CZT6	Cmss1	Protein CMSS1	Nucleus	Ribosome Assembly

Uniprot ID	Gene	Short name	Subcellular location	Gene ontology
Q14BA6	Medag	Mesenteric estrogen-dependent adipogenesis protein	Cytoplasm	Positive Regulation of Fat Cell Differentiation
Q9ERH8	Slc28a3	Solute carrier family 28 member 3	Plasma Membrane, Brush Border Membrane	Purine Nucleoside Transport, Pyrimidine Nucleoside Transport, Retina Homeostasis
Q3UQS8	Rbm20	RNA-binding protein 20	Nucleus, Cytoplasmic Ribonucleoprotein Granule	mRNA Processing, Regulation of RNA Splicing, Alternative mRNA Splicing
Q9D8S9	Bola1	BolA-like protein 1	Mitochondrion	Iron-Sulfur Cluster Assembly
A2AE20	Spaca5	Sperm acrosome-associated protein 5	Acrosomal Vesicle, Extracellular Region	Fusion of Sperm to Egg Plasma Membrane, Metabolic Process
O08796	Eef2k	Eukaryotic elongation factor 2 kinase	Dendritic Spine, Cytoplasm	Negative Regulation of Apoptosis, Phosphorylation, Translational Elongation, Positive Regulation of Synapse Assembly, Response to Ischemia
Q5U4C9	Dyrk2	Dual specificity tyrosine-phosphorylation-regulated kinase 2	Cytoplasm, Nucleus, Cytoskeleton	Protein Phosphorylation, Negative Regulation of Calcineurin-NFAT Signaling, Apoptotic Signaling, Glycogen Biosynthesis, Smoothened Signaling
O35658	C1qbp	Complement component 1 Q subcomponent-binding protein, mitochondrial	Mitochondrion, Nucleus, Plasma Membrane	Apoptotic Process, Innate Immune Response, Complement Activation, RNA Splicing, Negative Regulation of Virus Defense
Q9CZT6	Cmss1	Protein CMSS1	Nucleus	Ribosome Assembly
Q60997	Dmbt1	Deleted in malignant brain tumors 1 protein	Extracellular matrix, Plasma membrane	Blastocyst development, Defense response, Protein transport, Epithelial cell differentiation, Zymogen activation
Q920S2	Prss41	Serine protease 41	Plasma membrane, Intracellular organelle	Proteolysis
Q6ZPY5	Znf507	Zinc finger protein 507	Nucleus	Positive regulation of transcription by RNA polymerase II, Regulation of transcription, DNA-templated transcription
Q8VIH1	Nobox	Homeobox protein NOBOX	Nucleus, Cytoplasm	Oogenesis, Ovarian follicle development, Positive regulation of transcription, Transcription regulation, Transcription by RNA polymerase II
Q3UFM5	Nom1	Nucleolar MIF4G domain-containing protein 1	Nucleolus	Hair follicle maturation, Ribosomal small subunit biogenesis
Q9QXN0	Shroom3	Protein Shroom3	Apical plasma membrane, Cytoskeleton	Actin cytoskeleton organization, Cell morphogenesis, Regulation of cell shape, Neural tube closure, Apical protein localization
Q8R2R6	Mtg1	Mitochondrial ribosome-associated GTPase 1	Mitochondrion	Mitochondrial translation, Regulation of mitochondrial translation, Regulation of respiratory system process
O54826	Mllt10	Protein AF-10	Nucleus	Positive regulation of transcription by RNA polymerase II, Regulation of transcription

Uniprot ID	Gene	Short name	Subcellular location	Gene ontology
Q9D7I8	Fam83d	Protein FAM83D	Cytoplasm, Nucleus	Cell division, Cell migration, Positive regulation of cell cycle, Epidermal growth factor receptor signaling, Signal transduction
O70263	Lnx1	E3 ubiquitin-protein ligase LNX	Cytoplasm	Protein ubiquitination, Ubiquitin-dependent protein catabolic process
P56974	Nrg2	Pro-neuregulin-2, membrane-bound isoform	Extracellular region, Plasma membrane	Animal organ development, Cell surface receptor signaling pathway, Nervous system development, ERBB signaling pathways, Regulation of synapse assembly
Q8K2X3	Stn1	CST complex subunit STN1	Nucleus	Negative regulation of telomere maintenance, Positive regulation of DNA replication, Telomere capping, Telomere maintenance
Q30KP3	Defb20	Beta-defensin 20	Extracellular region	Defense response to bacterium, Innate immune response
P97813	Pld2	Phospholipase D2	Plasma membrane, Brush border membrane	G protein-coupled receptor internalization, Intracellular signal transduction, Neuron projection development, Phosphatidic acid biosynthesis, Positive regulation of cell adhesion
Q9CY34	Ube2f	NEDD8-conjugating enzyme UBE2F	Nucleus	Protein neddylation
Q8BR76	Tmem67	Meckelin (Meckel syndrome type 3 protein homolog)	Cilium, Plasma membrane	Cilium assembly, Branching morphogenesis of an epithelial tube, Determination of left/right symmetry, Heart development, Kidney development
Q9CZS1	Aldh1b1	Aldehyde dehydrogenase X, mitochondrial	Cytosol, Mitochondria	Ethanol catabolic process
Q8C2A2	Tsen54	tRNA-splicing endonuclease subunit Sen54	Nucleolus	MRNA processing, tRNA-type intron splice site recognition and cleavage
Q5SWY7	Fam83g	Protein FAM83G	Cytosol, Nucleus	BMP signaling pathway, Epidermal growth factor receptor signaling, Cell migration
Q9D1N9	Mrpl21	Large ribosomal subunit protein bL21m	Mitochondria	Mitochondrial translation
Q9WUF3	Casp8ap2	CASP8-associated protein 2	Cytoplasm, Nucleus	Cell cycle, Extrinsic apoptotic signaling pathway via death domain receptors, Fas signaling pathway
Q9JJV9	Scn5a	Sodium channel protein type 5 subunit alpha	Plasma membrane, Axon	Cardiac ventricle development, Membrane depolarization during action potential, Regulation of heart rate, Sodium ion transmembrane transport, Positive regulation of epithelial cell proliferation
Q8CDV6	Ccdc63	Coiled-coil domain-containing protein 63	Axoneme	Cilium movement, Outer dynein arm assembly, Spermatid development
Q8BHD8	Pcmtd2	Protein-L-isoaspartate O-methyltransferase domain-containing protein 2	Cytoplasm	Protein modification process

Uniprot ID	Gene	Short name	Subcellular location	Gene ontology
Q9Z1W4	Gdf11	Growth/differentiation factor 11	Extracellular space, Nucleoplasm	Animal organ morphogenesis, Neuron differentiation, Skeletal system development, Positive regulation of SMAD protein signal transduction, Pancreas development
Q8BPM6	Ric3	Protein RIC-3	Endoplasmic reticulum, Neuron projection	Positive regulation of cytosolic calcium ion concentration, Protein localization to cell surface, Synaptic transmission, cholinergic
Q8R2T8	Gtf3c5	General transcription factor 3C polypeptide 5	Nucleoplasm	Skeletal muscle cell differentiation, Transcription initiation at RNA polymerase III promoter
Q64692	St8sia4	CMP-N-acetylneuraminate-poly-alpha-2,8-sialyltransferase	Extracellular region, Golgi apparatus	Ganglioside biosynthetic process, Protein glycosylation, N-glycan processing, Oligosaccharide metabolic process, Sialylation
Q09M05	Agbl1	Cytosolic carboxypeptidase 4	Cytosol	C-terminal protein deglutamylation, Protein side chain deglutamylation, Proteolysis
Q8K1N4	Spats2	Spermatogenesis-associated serine-rich protein 2	Cytoplasm, Cytosol	-
P56564	Slc1a3	Excitatory amino acid transporter 1	Plasma membrane, Neuron projection	Auditory behavior, Cell morphogenesis involved in neuron differentiation, Positive regulation of synaptic transmission, L-glutamate transmembrane transport, Cranial nerve development
P29974	Cnga1	Cyclic nucleotide-gated channel alpha-1	Plasma membrane, Photoreceptor outer segment	Membrane depolarization, Monoatomic cation transmembrane transport, Regulation of cytosolic calcium ion concentration, Visual perception, Response to stimulus
Q80UG2	Plxna4	Plexin-A4	Plasma membrane, Neuron projection	Axon guidance, Nervous system development, Regulation of cell migration, Positive regulation of axonogenesis, Motor neuron axon guidance
P54761	Ephb4	Ephrin type-B receptor 4	Plasma membrane, Dendrite	Angiogenesis, Axon guidance, Cell adhesion, Heart morphogenesis, Protein autophosphorylation
Q9Z0N2	Eif2s3y	Eukaryotic translation initiation factor 2 subunit 3, Y-linked	Cytosol	Formation of translation preinitiation complex
Q9CWP6	Mospd2	Motile sperm domain-containing protein 2	Endoplasmic reticulum membrane, Endomembrane system	Chemotaxis, Lipid droplet formation
Q68FF6	Git1	ARF GTPase-activating protein GIT1	Cytosol, Mitochondrion, Dendrite	Brain development, Synaptic vesicle recycling, Regulation of synaptic vesicle exocytosis, Neuron development, Motor learning
Q8BK62	Olfml3	Olfactomedin-like protein 3	Extracellular space	Signal transduction
Q69Z37	Samd9l	Sterile alpha motif domain-containing protein 9-like	Cytoplasm, Early endosome, Mitochondrion	Hematopoietic progenitor cell differentiation, Stem cell division, Spleen development, Regulation of protein catabolic process, Common myeloid progenitor cell proliferation

Uniprot ID	Gene	Short name	Subcellular location	Gene ontology
Q05A13	Sdr16c6	Short-chain dehydrogenase/reductase family 16C member 6	Lipid droplet	-
Q0P557	Spata18	Mitochondria-eating protein	Cytoplasm, Mitochondrial outer membrane, Sperm flagellum	Cell differentiation, DNA damage response, Spermatogenesis, Mitophagy
Q9EQC1	Hsd3b7	3 beta-hydroxysteroid dehydrogenase type 7	Endoplasmic reticulum membrane, Lipid droplet	Steroid biosynthetic process, Cholesterol catabolic process, Regulation of cell growth, B cell chemotaxis, — (fifth less significant)
P15539	Cyp11b2	Cytochrome P450 11B2, mitochondrial	Mitochondrial inner membrane, Dendrite	Aldosterone biosynthetic process, Cholesterol metabolic process, Regulation of blood volume, Cortisol metabolic process, Cellular response to peptide hormone stimulus
Q3UNZ8	Cryzl2	Quinone oxidoreductase-like protein 2	Mitochondrion	-
O88839	Adam15	Disintegrin and metalloproteinase domain-containing protein 15	Plasma membrane, Cell surface, Extracellular space	Angiogenesis, Cell adhesion, Proteolysis, Immune response, Tissue regeneration
Q8BP78	Fra10ac1	Protein FRA10AC1 homolog	Nucleus	Dephosphorylation; MRNA Splicing Via Spliceosome
Q8BH55	Thnsl1	Threonine synthase-like 1	Cytoplasm	-
P39053	Dnm1	Dynamin-1	Cytoplasm; Plasma Membrane; Golgi Apparatus	Clathrin Coat Assembly Involved In Endocytosis; Endosome Organization; G Protein-Coupled Receptor Internalization
Q6X786	Zpbp2	Zona pellucida-binding protein 2	Extracellular Region; Cell Body	Acrosome Assembly; Binding Of Sperm To Zona Pellucida; Circadian Regulation Of Gene Expression
Q8BGE7	Trim6	Tripartite motif-containing protein 6	Cytoplasm; Nucleus	Antiviral Innate Immune Response; Cellular Response To Virus; Positive Regulation Of Cytokine Production
Q9DBX3	Susd2	Sushi domain-containing protein 2	Extracellular Space; Plasma Membrane	Negative Regulation Of Cell Cycle G1/S Phase Transition; Negative Regulation Of Cell Division
P06802	Enpp1	Ectonucleotide pyrophosphatase/phosphodiesterase family member 1	Plasma Membrane; Extracellular Space; Cell Body	Adipose Tissue Development; Bone Development; ATP Metabolic Process; Cellular Homeostasis; Response To Insulin
Q9CUB6	Otud1	OTU domain-containing protein 1	-	Protein K63-Linked Deubiquitination; Proteolysis
Q3UTY6	Thsd4	Thrombospondin type-1 domain-containing protein 4	Extracellular Matrix	Elastic Fiber Assembly; Extracellular Matrix Organization; Microfibril Assembly; Proteolysis
P57748	Mmp20	Matrix metalloproteinase-20	Extracellular Matrix; Extracellular Space	Amelogenesis; Collagen Catabolic Process; Extracellular Matrix Disassembly; Proteolysis
Q8R570	Snap47	Synaptosomal-associated protein 47	Plasma Membrane; Synapse	Exocytosis; Long-Term Synaptic Potentiation; Synaptic Vesicle Fusion To Presynaptic Active Zone
Q9DA21	Smco2	Single-pass membrane and coiled-coil domain-containing protein 2	Membrane	-

Uniprot ID	Gene	Short name	Subcellular location	Gene ontology
Q9D6P8	Calml3	Calmodulin-like protein 3	-	-
Q8CH02	Sugp1	SURP and G-patch domain-containing protein 1	Nucleoplasm	MRNA Processing; RNA Splicing
P47750	Tshr	Thyrotropin receptor	Plasma Membrane	Adenylate Cyclase-Activating G Protein-Coupled Receptor Signaling
P36536	Sar1a	Small COPII coat GTPase SAR1A	Endoplasmic reticulum, Golgi apparatus	COPII vesicle coating; Intracellular protein transport; Vesicle-mediated transport; Membrane organization
B1AQJ2	Usp36	Ubiquitin carboxyl-terminal hydrolase 36	Cytoplasm, nucleus	Protein deubiquitination; Regulation of apoptotic process; Proteolysis; Protein stabilization; Regulation of protein stability
A2AJT4	Pnisr	Arginine/serine-rich protein PNISR	Nucleus	—
Q80TV8	Clasp1	CLIP-associating protein 1	Cytoplasm, Golgi apparatus	Cell division; Cell migration; Microtubule nucleation; Establishment of cell polarity; Mitotic spindle assembly
O70326	Grem1	Gremlin-1	Cell surface, extracellular region	Negative regulation of apoptotic process; Positive regulation of angiogenesis; Signal transduction; Regulation of epithelial to mesenchymal transition; Cell migration involved in sprouting angiogenesis
Q62167	Ddx3x	ATP-dependent RNA helicase DDX3X	Cytoplasm, nucleus	Positive regulation of transcription by RNA polymerase II; Negative regulation of apoptotic process
Q3TYA6	Mphosph8	M-phase phosphoprotein 8	Cytoplasm, nucleus	Negative regulation of gene expression via chromosomal CpG dinucleotide methylation; Positive regulation of DNA
Q8VC82	Rhbdl1	Rhomboid-related protein 1	Membrane	Proteolysis
E9Q8T2	Prdm15	PR domain zinc finger protein 15	Cytoplasm, nucleus	Positive regulation of transcription by RNA polymerase II; Cell fate commitment; Methylation; Regulation of stem cell division; Negative regulation of MAPK cascade
Q6R2P8	Neil2	Endonuclease 8-like 2	Cytoplasm, nucleus	Base-excision repair
Q8R420	Abca3	Phospholipid-transporting ATPase ABCA3	Plasma membrane, intracellular membrane-bounded organelle	Lipid transport; Phospholipid transport; Lung development; Surfactant homeostasis; Regulation of lipid transport
Q3TUF7	Yeats2	YEATS domain-containing protein 2	Nucleus	Chromatin remodeling; Regulation of transcription by RNA polymerase II
Q5SSL4	Abr	Active breakpoint cluster region-related protein	Axon, cytosol	Cell migration; Actin cytoskeleton organization; Phagocytosis; Regulation of vascular permeability; Negative regulation of inflammatory response
Q99K82	Smox	Spermine oxidase	Cytosol, nucleus	Spermine catabolic process

Uniprot ID	Gene	Short name	Subcellular location	Gene ontology
Q9WVM3	Anapc7	Anaphase-promoting complex subunit 7	Cytoplasm, nucleus	Cell cycle; Cell division; Protein ubiquitination; Positive regulation of mitotic metaphase/anaphase transition; Brain development
Q9D5A0	Spesp1	Sperm equatorial segment protein 1	Acrosomal vesicle	Acrosome reaction, Fertilization, Sperm-egg recognition
Q61398	Pcolce	Procollagen C-endopeptidase enhancer 1	Extracellular matrix, Extracellular space	Proteolysis, Cellular response to leukemia inhibitory factor, Collagen-containing extracellular matrix, Extracellular matrix organization, Collagen biosynthesis
Q9R2B6	St6galnac4	Alpha-N-acetyl-neuraminyl-2,3-beta-galactosyl-1,3-N-acetyl-galactosaminide alpha-2,6-sialyltransferase	Golgi apparatus	Ganglioside biosynthetic process, Oligosaccharide metabolic process, Protein glycosylation, Glycoprotein biosynthesis, Carbohydrate metabolism
P15864	H1f2	Histone H1.2	Nucleus	Chromatin organization, Chromosome condensation, Negative regulation of transcription,
Q3U564	Dcp1b	mRNA-decapping enzyme 1B	Nucleus, P-body	mRNA decapping, Nonsense-mediated mRNA decay, Response to interleukin-17, Nuclear mRNA catabolic process, Regulation of mRNA stability
Q99LB6	Mat2b	Methionine adenosyltransferase 2 subunit beta	Cytoplasm, Mitochondrion, Nucleus	One-carbon metabolic process, S-adenosylmethionine biosynthetic process, Polysaccharide biosynthesis, Methylation, Cellular metabolic process
Q9JM73	Srf	Serum response factor	Nucleus, Cytoplasm	Actin cytoskeleton organization, Cardiac muscle differentiation, Regulation of transcription, Cell migration, Neuron projection development
Q8BQ30	Ppp1r18	Phostensin	Cytoplasm, Cytoskeleton	Cytoskeleton organization, Protein phosphatase regulation, Actin filament organization, Signal transduction, Cytoplasmic transport
P97427	Crmp1	Dihydropyrimidinase-related protein 1	Cytoplasm, Cytoskeleton, Neuron	Semaphorin-plexin signaling pathway, Neuron projection development, Axon guidance, Cytoskeleton organization, Neural development
Q8QZV6	Fam161a	Protein FAM161A	Cytoplasm, Centriole	Cilium assembly, Cilium organization, Photoreceptor development, Organelle biogenesis, Microtubule-based process
Q9WV66	Marchf7	E3 ubiquitin-protein ligase MARCHF7	Cytoplasm, Nucleus, Plasma membrane	Negative regulation of cilium assembly, Protein ubiquitination, DNA damage response, Cell proliferation regulation, Immune response regulation
Q7JJ13	Brd2	Bromodomain-containing protein 2	Nucleus, Cytoplasm	Chromatin remodeling, Regulation of transcription, Neural tube closure, T-helper 17 cell differentiation, Gene expression regulation

Uniprot ID	Gene	Short name	Subcellular location	Gene ontology
Q6P1H6	Ankle2	Ankyrin repeat and LEM domain-containing protein 2	Endoplasmic reticulum	Cell division, CNS development, Nuclear membrane reassembly, Negative regulation of apoptosis, Protein dephosphorylation
Q8C6P8	Zfp57	Zinc finger protein 57	Nucleus	Genomic imprinting, Epigenetic regulation, DNA methylation, Negative regulation of transcription, Post-fertilization gene regulation
Q61730	Il1rap	Interleukin-1 receptor accessory protein	Plasma membrane, Extracellular region	Cytokine-mediated signaling, Inflammatory response, Interleukin production regulation, NF-kappaB activation, Synapse assembly
Q8K1C9	Lrrc41	Leucine-rich repeat-containing protein 41	Cytoplasm, Nucleus	Protein ubiquitination, Regulation of protein stability, Cell cycle regulation, Signal transduction, Cellular response to stress
Q80U38	Khnyn	Protein KHNYN	Cytoplasm, Nucleus	RNA processing, RNA stabilization, RNA export, Gene expression regulation, Post-transcriptional regulation
P01626	Ig kappa	Ig kappa chain V-II region MOPC 167	Extracellular space	Adaptive immune response, Immune response, Antigen binding, Immunoglobulin production, Defense response
Q9D5E4	Drc3	Dynein regulatory complex subunit 3	Axoneme, Cilium, Flagellum	Cilium assembly, Microtubule-based movement, Cell motility, Flagellum organization, Signal transduction
Q8BGS0	Mak16	Protein MAK16 homolog	Nucleolus	rRNA processing, Ribosome biogenesis, RNA maturation, Ribosomal subunit assembly, RNA metabolic process
Q9JJT9	Phax	Phosphorylated adapter RNA export protein	Nucleus, Cytoplasm	Protein transport, RNA stabilization, snRNA export, Nuclear export, RNA processing
Q9Z2G6	Sel1l	Protein sel-1 homolog 1	Endoplasmic reticulum	ER-associated degradation, Notch signaling, Protein secretion, Response to ER stress, Retrograde transport
Q8VIM6	Strc	Stereocilin	Cell surface, Kinocilium	Sensory perception of sound, Cell adhesion, Auditory receptor organization, Mechanical stimulus detection, Signal transduction
Q8CH18	Ccar1	Cell division cycle and apoptosis regulator protein 1	Nucleus	Apoptotic process, Cell cycle, Positive regulation of apoptosis, Cell migration, Transcription regulation
Q02284	Htr1f	5-hydroxytryptamine receptor 1F	Plasma membrane, Dendrite	G protein-coupled receptor signaling, Chemical synaptic transmission, Signal transduction, Neurotransmitter signaling, Adenylate cyclase inhibition
Q5SWD9	Tsr1	Pre-rRNA-processing protein TSR1 homolog	Nucleolus	rRNA processing, Endonucleolytic cleavage, Ribosome biogenesis, RNA maturation, RNA metabolic process
O35633	Slc32a1	Vesicular inhibitory amino acid transporter	Plasma membrane, Synapse	Neurotransmitter transport, GABA transport, Amino acid transport, Synaptic vesicle loading, Synaptic transmission
P28776	Ido1	Indoleamine 2,3-dioxygenase 1	Cytoplasm, Cytosol	NAD biosynthesis, Defense response, Inflammatory response, T cell proliferation regulation, Response to stress

Uniprot ID	Gene	Short name	Subcellular location	Gene ontology
Q8BUL5	Klhl7	Kelch-like protein 7	Cytosol, Nucleolus	Protein ubiquitination, Ubiquitin ligase complex, Protein degradation, Cell cycle regulation, Cellular protein catabolic process
Q80U93	Nup214	Nuclear pore complex protein Nup214	Nuclear envelope, Nuclear pore	Nuclear transport, mRNA export, Protein export, Cell cycle regulation, Nucleocytoplasmic transport
Q8BHN1	Txlng	Gamma-taxilin (Factor inhibiting ATF4-mediated transcription)	Cytosol, Nuclear membrane	Cell cycle, Regulation of bone mineralization, Regulation of cell cycle, Regulation of cell cycle process
Q14AK4	Zdhhc11	Palmitoyltransferase ZDHHC11	Endoplasmic reticulum, Golgi apparatus, Endosome membrane	Antiviral innate immune response, Protein palmitoylation, Positive regulation of defense response to virus, Protein targeting to membrane
Q921I2	Klhdc4	Kelch domain-containing protein 4	-	-
Q61006	Musk	Muscle, skeletal receptor tyrosine-protein kinase	Plasma membrane, Neuromuscular junction, Cell projection	Cell differentiation, Synaptic transmission, Receptor signaling, Gene expression regulation, Neuromuscular junction development
Q9WVI9	Mapk8ip1	C-Jun-amino-terminal kinase-interacting protein 1	Cytoplasm, Membrane, Nucleus, Neuron projection, Synapse	JNK cascade regulation, Negative regulation of apoptosis, Signal transduction, Vesicle-mediated transport, Regulation of transcription
P60755	Mdga2	MAM domain-containing glycosylphosphatidylinositol anchor protein 2	Plasma membrane, Synapse	Nervous system development, Neuron migration, Pattern specification, Presynapse assembly regulation, Synaptic membrane adhesion
Q65Z40	Wapl	Wings apart-like protein homolog	Chromatin, Cytoplasm, Nucleoplasm	Cell division, Sister chromatid segregation, Negative regulation of DNA replication, Regulation of cohesin loading, Chromatin binding
Q8BHP2	Nutm1	NUT family member 1	Cytoplasm, Nucleus	-
O35683	Ndufa1	NADH dehydrogenase [ubiquinone] 1 alpha subcomplex subunit 1	Mitochondrial inner membrane	Aerobic respiration, Mitochondrial respiratory chain assembly, ATP synthesis
P18653	Rps6ka1	Ribosomal protein S6 kinase alpha-1	Cytoplasm, Nucleoplasm, Spindle	Hepatocyte proliferation, Signal transduction, Negative regulation of apoptosis, Phosphorylation, Positive regulation of transcription
Q6PE65	Gpatch2l	G patch domain-containing protein 2-like	Nucleus	-
G5E869	Znf142	Zinc finger protein 142	Nucleus	Positive regulation of transcription by RNA polymerase II
Q8BHN1	Txlng	Gamma-taxilin (Factor inhibiting ATF4-mediated transcription)	Cytosol, Nuclear membrane	Cell cycle, Regulation of bone mineralization, Regulation of cell cycle, Regulation of cell cycle process

Uniprot ID	Gene	Short name	Subcellular location	Gene ontology
Q14AK4	Zdhhc11	Palmitoyltransferase ZDHHC11	Endoplasmic reticulum, Golgi apparatus, Endosome membrane	Antiviral innate immune response, Protein palmitoylation, Positive regulation of defense response to virus, Protein targeting to membrane
Q921I2	Klhdc4	Kelch domain-containing protein 4	-	-
