# Supplementary material for: Elektra-Qki and Alien-Wt1 lncRNA-protein interaction controls myocardial ion channel expression and epicardial EMT during heart development in mice
Source: Cell Mol Life Sci. 2026 Jul 7;83(1):356. doi: 10.1007/s00018-026-06322-x (PMC13631190; doi:10.1007/s00018-026-06322-x)
Supplement: Supplementary file 10 [file 18_2026_6322_MOESM7_ESM.pdf]

RT-qPCR primers	
Mm_Elektra	Fw: TGGATCAGCAGAAGAGGGTG
	Rv: AGGACCAAGCTAGGTTCTGC
Mm_Xist (isoform 2)	Fw: CCGTTCCTCACACCAGACTG
	Rv: AACAGCAAACTACCCCACCC
Mm_Mef2c	Fw: TGGTTTCCGTAGCAACTCCT
	Rv: AGTTACAGAGCCGAGGTGGA
Mm_Pitx2c	Fw: CCTCACCCTTCTGTCACCAT
	Rv: GCCCACATCCTCATTCTTTC
Mm_Srf	Fw: GCTCAATGCCTTCTCTCAGG
	Rv: CCCTATCACAGCCATCTGGT
Mm_Nkx2.5	Fw: TTGGCGTCGGGGACTTGAAC
	Rv: GGTGGGTGTGAAATCCGAGGGAC
Mm_Qki	Fw: GGGCCTGAAGCTGGGTTAAT
	Rv: TTCTTACCGTTCGCTTGGGG
Mm_Ryr2	Fw: AGTGGGTTGAGCTATGCAGA
	Rv: ACAAGAGCCTCCTTCTGTCC
Mm_Scn5a	Fw: TGGTGGAGACAGATGACCAA
	Rv: GCAGCCAGCTTGACAATACA
Mm_Kcnn3	Fw: ACTTCAACACCCGATTCGTC
	Rv: GGAAAGGAACGTGATGGAGA
Mm_Kcnk3	Fw: TGTGCACCTTCACCTACCTG
	Rv: GGTGATGGCGAAGTAGAAGG
Mm_Cacna1c	Fw: TCCTGGTCTGAGGAGACGA
	Rv: GGTGGTGACCTCGATGAACT
Mm_Cacnb2	Fw: GCCGACTATCTGGAGGCATA
	Rv: GGCCTTTGATCACCTTGAGA
Mm_Cacnb3	Fw: ATCCCTGGACTTCAGAACCA
	Rv: TGGTACAGGTCCTGGTAGG
Mm_Cacnb4	Fw: TCTCACCATATCCCACAGCA
	Rv: CAAGCGGTTCCTACTCTTGC
Mm_Casq2	Fw: TTGACCTGTTCAAGCCACAG
	Rv: CACCATCATCATCTTCATCTTCA
Mm_Ncx1	Fw: TTCATTGGAGACCTGGCTTC
	Rv: TGCTGTGAATGTCTTCCTGG
Mm_Atp2a2	Fw: CTGTGGAGACCCTTGGTTGT
	Rv: TCCAATTGGTGCATATGTGG
Mm_Alien	Fw: TGTGCACACATGGCTCAGC
	Rv: GAGTTGGGAGGCAGCAGAC
Mm_Wt1	Fw: AGGTTTTCTCGCTCAGACCA
	Rv: GCTGAAGGGCTTTTCACTTG
Mm_Snai1	Fw: CTTGTGTCTGCACGACCTGT
	Rv: AGTGGGAGCAGGAGAATGG
Mm_Snai2	Fw: CATTGCCTTGTGTCTGCAAG
	Rv: GATGTGCCCTCAGGTTTGAT
Mm_Cdh1	Fw: CGACCGGAAGTGACTCGAAA
	Rv: AGTCCCCTAGTCGTCCTCAC
Mm_Cdh2	Fw: TCCTTGTTCTCATGTTTGTGGT
	Rv: TTAATGAAGTCCCCAATATCCCCA
Mm_Cdh5	Fw: TGCATCCTCACCATCACAGT
	Rv: AGTGACCAACTGCTCGTGAA

Mm_Wnt8a	Fw: ACGTGGAGTTCGGGGAAAAA
	Rv: TTTCCCATCTGCCGGAAGTC
Mm_Wnt11	Fw: GCCAAGTTTTCCGATGCTCC
	Rv:GTCTTGAGGTCAGCAGCCAC
Mm_Foxf1	Fw: GGCCTCCTACATCAAGCA
	Rv: CTGGGCGACTGTGAGTGATA
Mm_Nr2f1	Fw: CAAAGCCATCGTGCTATTCA
	Rv: GCTCGATGACAGAGGAGGAC
Mm_Nr2f2	Fw: GACTCCGCCGAGTATAGCTG
	Rv: CCTACCAAACGGACGAAAAA
Mm_Gsk3ß	Fw: CCTTATCCCTCCACATGCTC
	Rv: ATTGGTCTGTCCACGGTCTC
Mm_Fzd6	Fw: TTAATTGTTGGTATCTCTGCGGT
	Rv: CCTATGAGGCCCTGGTGC
Mm_Dkk3	Fw: CTGGGAACTGGAGCCTGAAG
	Rv: GAAGGCTGGCTTGCACATGTA
Mm_Gapdh	Fw: GGCATTGCTCTCAATGACAA
	Rv: TGTGAGGGAGATGCTCAGTG
Cloning primers	
Mm_Elektra_GOF	Fw: GAATTCCTCTTCTTTCTTTTACTCCCATTTCCTTCCAGG
	Rv: AAGCTTGGATATGTACAGGAGCCAGAGGAGACC
Biotinylated RNA primers	
Mm_Elektra_B1	Fw: AGTAATACGACTCACTATAGGGACTCCCATTTCCTTCCAGGT
	Rv: GCCTTCTGGCTTGTAGGATG
Mm_Elektra_B2	Fw: AGTAATACGACTCACTATAGGGCTACAAGCCAGAAGGCCAAG
	Rv: ATATGTACAGGAGCCAGAGGAGACCA
Mm_Alien_B1	Fw: AGTAATACGACTCACTATAGGGTAAATGCAGGGATGCCTCGG
	Rv: TGAACTTCAGCACTTGCCCA
Mm_Alien_B2	Fw: AGTAATACGACTCACTATAGGGACATTGAACATAAGTGGTGTGGA
	Rv: ACTCAGTGGTACTTCATTGGAGA
Mm_Alien_B3	Fw: AGTAATACGACTCACTATAGGGTGTGGAGCCTGACTGTGAAA
	Rv: GGGAGTCCAGCATATCAGCC
Mm_Alien_B4	Fw: AGTAATACGACTCACTATAGGGGGGACGGTGCCTGTAACTTA
	Rv: TGAGGGTAGAGTCAGCTATTTCA
Mm_Alien_B5	Fw: AGTAATACGACTCACTATAGGGATGCACCCTCGTACAAAGCA3
	Rv: AGCAGAGATGAACAGGTGCC
Mm_Alien_B6	Fw: AGTAATACGACTCACTATAGGGTGCAGAACCGTGCCACTG
	Rv: GGCTGCCAAATGTCCAGAAG
Gapdh_B	Fw: AGTAATACGACTCACTATAGGGCAGCAAGGACACTGAGCAAG
	Rv: GGGTGCAGCGAACTTTATTG
siRNAs	
siPitx2c	S 5’ GUGCAUACAAUCUCCGAUAUU<br>AS 3' UUCACGUAUGUUAGAGGCUAU
siMef2c	S 5’ GGGUCGAUAUGCAUUAUAUUU<br>AS 3’ UUCCCAGCUAUACGUAAUAUA
siSrf	S 5' GACGGCACCACUUAUUUAUUU<br>AS 3’ UUCUGCCGUGGUGAAUAAAUA
siNkx2.5	S 5' CCAUUAAAGUGAGUGCGCUUU<br>AS 3’ UUGGUAAUUUCACUCACGCGA
siQKI	S 5' GACACGUUAAAUGGCAGUAUU<br>AS 3' UUCUGUGCAAUUUACCGUCAU

siElektra	S 5' CUGGAUGUGAGUUCAAAGAUU<br>AS 3' UUGACCUACACUCAAGUUUCU
siWt1	S 5' AAGCUGUCCCACUUACAGAUGCTT<br>AS 3' TTUUCGACAGGGUGAAUGUCUACG
ASOs	
ASO_Elektra	mG*mG*mA*mG*mA*G*G*C*T*C*C*T*G*T*A*C*A*mA*mU*mA*mG*mG
ASO1_Alien	mC*mC*mC*mU*mC*A*G*G*C*A*C*A*T*T*C*A*C*mA*mA*mC*mC*mU
ASO2_Alien	mC*mG*mU*mG*mU*A*C*T*C*C*C*T*A*G*G*mC*mA*mU*mU*mU
SCRINSHOT padlock probes	
Mm_Tnnt2_pr1	ACAGTTTCATCTATTTCCAACCCTCTATGATTACTGACTGCGTCTATTTAGAGAGAA<br>GTAGAAGAAGATCCTGG CACAGCTTTGACGAGA
Mm_Col1a2_pr1	GAGTTGCATCAACTTCATAGTCCTCTATGATTACTGACTGCGTCTATTTAGTGGAGC<br>CTATCCTATCTTCTTTATCTGGTTATTGAGAGATTTCA
Mm_Col1a2_pr2	TTAGCACCTTTGTGTTGCATCCTCTATGATTACTGACTGCGTCTATTTAGTGGAGCC<br>TATCCTATCTTCTTTTCAAGTATCTGAAATACTACTAA
Mm_Col1a2_pr3	AATGCTGAATCTAATGAAGAGTCCTCTATGATTACTGACTGCGTCTATTTAGTGGA<br>GCCTATCCTATCTTCTTTAAATGAGGCTGTTAAAGAATG
Mm_Pecam_pr1	TGGACCAAGTGTGTCACTTGTCCTCTATGATTACTGACTGCGTCTATTTAGTGGAG<br>CCACATCTATCTTCTTTGATGATAATTTCTGTAAACTCC
Mm_Pecam_pr2	CATTTCAGCCTCAGAATATTCTCCTCTATGATTACTGACTGCGTCTATTTAGTGGAG<br>CCACATCTATCTTCTTTCTGTCTATCCGAGACACAA
Mm_Elektra_pr1	CGGTTACAGGCTTTAGATAG TGATCCTCTATGA TTACTGACTGCGTCTATTTA<br>CCTTCTGGCTTGTAGGATGCTATCAGATCCAGTGAAGCT
Mm_Elektra_pr2	TCAATCAGGTATCTTCAGATATTTGTGATCCTCTATGATTACTGACTGCGTCTATTT<br>ACCTTCTGGCTTGTAGGATGC CCTCTTCTGCTGATCCATG
Mm_Alien_pr1	ACACCTGTTCATCACATTTTGATCCTCTATGATTACTGACTGCGTCTATTTACTACTT<br>TGGAGGCACAACTCCCTGAATCCAAAGTAAATGG
Mm_Alien_pr2	AAAGGGATGTTAACTACTGATATGATCCTCTATGATTACTGACTGCGTCTATTTACT<br>ACTTTGGAGGCACAACTCCTGGAAACAGCTGATAGG
SCRINSHOT detection oligos	
Mm_Tnnt2_pr1	GAGAGAAGUAGAAGAAGAUCCTGG[Cy5]
Mm_Col1a2_pr1	TATUGAGAGATUTCAGAGTTGCAUCAAC [Cy5]
Mm_Col1a2_pr2	TATCUGAAATACTACUAATTAGCACCTTTGUG [Cy5]
Mm_Col1a2_pr3	GGCTGTTAAAGAAUGAATGCTGAATCUA [Cy5]
Mm_Pecam_pr1	TCTGUAAACTCCUGGACCAAGUG [Cy3]
Mm_Pecam_pr2	CCGAGACACAACAUUUCAGCC [Cy3]
Mm_Elektra_pr1	CCTTCUGGCTTGUAGGATGC [FITC]
Mm_Alien_pr1	CTACUTTGGAGGCACAACUC [Cy3]
