# Supplementary material for: Elektra-Qki and Alien-Wt1 lncRNA-protein interaction controls myocardial ion channel expression and epicardial EMT during heart development in mice
Source: Cell Mol Life Sci. 2026 Jul 7;83(1):356. doi: 10.1007/s00018-026-06322-x (PMC13631190; doi:10.1007/s00018-026-06322-x)
Supplement: Supplementary file 11 [file 18_2026_6322_MOESM8_ESM.pdf]

ANOVA Summary for Figure 1B (*Elektra* mRNA level)

Tukey's multiple comparisons test	Summary	Adjusted P Value
Head vs. Heart	ns	0.3221
Head vs. Lung	ns	0.5628
Head vs. Stomach	ns	0.7061
Head vs. Kidney	****	<0.0001
Heart vs. Lung	ns	0.9872
Heart vs. Stomach	ns	0.9422
Heart vs. Kidney	****	<0.0001
Lung vs. Stomach	ns	0.9989
Lung vs. Kidney	****	<0.0001
Stomach vs. Kidney	****	<0.0001

ANOVA Summary for Figure 1B (*Alien* mRNA level)

Tukey's multiple comparisons test	Summary	Adjusted P Value
Head vs. Heart	ns	0.4981
Head vs. Lung	****	<0.0001
Head vs. Stomach	****	<0.0001
Head vs. Kidney	ns	0.5581
Head vs. Intestine	ns	0.8661
Head vs. Liver	****	<0.0001
Heart vs. Lung	****	<0.0001
Heart vs. Stomach	****	<0.0001
Heart vs. Kidney	ns	>0.9999
Heart vs. Intestine	ns	0.0776
Heart vs. Liver	****	<0.0001
Lung vs. Stomach	****	<0.0001
Lung vs. Kidney	****	<0.0001
Lung vs. Intestine	****	<0.0001
Lung vs. Liver	*	0.0252
Stomach vs. Kidney	****	<0.0001
Stomach vs. Intestine	****	<0.0001
Stomach vs. Liver	****	<0.0001
Kidney vs. Intestine	ns	0.0930
Kidney vs. Liver	****	<0.0001
Intestine vs. Liver	****	<0.0001

ANOVA Summary for Figure 1C (*Elektra* mRNA level)

Tukey's multiple comparisons test	Summary	Adjusted P Value
Ventricle vs. Left atrium	ns	0.7134
Ventricle vs. Right atrium	***	0.0004
Left atrium vs. Right atrium	***	0.0002

ANOVA Summary for Figure 1C (*Alien* mRNA level)

Tukey's multiple comparisons test	Summary	Adjusted P Value
Head vs. Ventricle	ns	>0.9999
Head vs. Left atrium	ns	>0.9999
Head vs. Right atrium	ns	>0.9999
Head vs. Lung	**	0.0041
Head vs. Stomatch	****	<0.0001
Head vs. Kidney	ns	>0.9999

Head vs. Intestine	ns	0.9991
Head vs. Liver	****	<0.0001
Head vs. Muscle	ns	>0.9999
Ventricle vs. Left atrium	ns	>0.9999
Ventricle vs. Right atrium	ns	>0.9999
Ventricle vs. Lung	**	0.0023
Ventricle vs. Stomatch	****	<0.0001
Ventricle vs. Kidney	ns	>0.9999
Ventricle vs. Intestine	ns	0.9907
Ventricle vs. Liver	****	<0.0001
Ventricle vs. Muscle	ns	>0.9999
Left atrium vs. Right atrium	ns	>0.9999
Left atrium vs. Lung	**	0.0024
Left atrium vs. Stomatch	****	<0.0001
Left atrium vs. Kidney	ns	>0.9999
Left atrium vs. Intestine	ns	0.9917
Left atrium vs. Liver	****	<0.0001
Left atrium vs. Muscle	ns	>0.9999
Right atrium vs. Lung	**	0.0024
Right atrium vs. Stomatch	****	<0.0001
Right atrium vs. Kidney	ns	>0.9999
Right atrium vs. Intestine	ns	0.9915
Right atrium vs. Liver	****	<0.0001
Right atrium vs. Muscle	ns	>0.9999
Lung vs. Stomatch	****	<0.0001
Lung vs. Kidney	**	0.0023
Lung vs. Intestine	*	0.0194
Lung vs. Liver	****	<0.0001
Lung vs. Muscle	**	0.0023
Stomatch vs. Kidney	****	<0.0001
Stomatch vs. Intestine	****	<0.0001
Stomatch vs. Liver	****	<0.0001
Stomatch vs. Muscle	****	<0.0001
Kidney vs. Intestine	ns	0.9905
Kidney vs. Liver	****	<0.0001
Kidney vs. Muscle	ns	>0.9999
Intestine vs. Liver	****	<0.0001
Intestine vs. Muscle	ns	0.9904
Liver vs. Muscle	****	<0.0001

ANOVA Summary for Figure 2A (*Elektra* mRNA level)

Tukey's multiple comparisons test	Summary	Adjusted P Value
HL1 vs. MEC1	****	<0.0001
HL1 vs. EPIC	****	<0.0001
HL1 vs. MEVEC	****	<0.0001
MEC1 vs. EPIC	ns	0.0971
MEC1 vs. MEVEC	****	<0.0001
EPIC vs. MEVEC	****	<0.0001

ANOVA Summary for Figure 2A (*Alien* mRNA level)

Tukey's multiple comparisons test	Summary	Adjusted P Value
HL1 vs. MEC1	****	<0.0001
HL1 vs. EPIC	****	<0.0001
HL1 vs. MEVEC	****	<0.0001
MEC1 vs. EPIC	***	0.0007
MEC1 vs. MEVEC	****	<0.0001
EPIC vs. MEVEC	****	<0.0001
