# Supplementary material for: Elektra-Qki and Alien-Wt1 lncRNA-protein interaction controls myocardial ion channel expression and epicardial EMT during heart development in mice
Source: Cell Mol Life Sci. 2026 Jul 7;83(1):356. doi: 10.1007/s00018-026-06322-x (PMC13631190; doi:10.1007/s00018-026-06322-x)
Supplement: Supplementary file 12 [file 18_2026_6322_MOESM9_ESM.pdf]

Uniprot ID	Gene	Short name	Subcellular location	Gene ontology
Q61315	Apc	Adenomatous polyposis coli protein	Cytoplasm, cytoskeleton, cell membrane	Canonical wnt signaling pathway, cell adhesion, cell migration, regulation of cell cycle, apoptotic process
Q8BGQ7	Aars1	Alanine--tRNA ligase, cytoplasmic	Cytoplasm, nucleus	Alanine-tRNA ligase activity, amino acid binding, ATP binding, tRNA binding, neuron apoptotic process
Q8BP47	Nars1	Asparagine--tRNA ligase, cytoplasmic	Cytoplasm	Asparagine-tRNA ligase activity, ATP binding, CCR3 chemokine receptor binding, nucleic acid binding, protein dimerization activity
Q8VHK9	Dhx36	ATP-dependent DNA/RNA helicase DHX36	Nucleus, cytoplasm, cytosol, mitochondrion	ATP binding, ATP hydrolysis activity, DNA helicase activity, RNA helicase activity, positive regulation of transcription by RNA polymerase II
Q62167	Ddx3x	ATP-dependent RNA helicase DDX3X	Cell membrane, Nucleus, Cytoplasm, Inflammasome, Lamellipodium	ATP binding, RNA helicase activity, positive regulation of apoptotic process, positive regulation of transcription by RNA polymerase II, positive regulation of translation
Q8K4L0	Ddx54	ATP-dependent RNA helicase DDX54	Nucleus, nucleolus	ATP binding, ATP hydrolysis activity, RNA binding, RNA helicase activity, transcription corepressor activity
O35161	Celsr1	Cadherin EGF LAG seven-pass G-type receptor 1	Cell membrane	G protein-coupled receptor activity, cell surface receptor signaling pathway, homophilic cell adhesion via plasma membrane adhesion molecules, regulation of actin cytoskeleton organization, wound healing
O08529	Capn2	Calpain-2 catalytic subunit	Cell membrane, cytoplasm	Calcium ion binding, calcium-dependent cysteine-type endopeptidase activity, Peptidase activity, Positive regulation of myoblast fusion, Proteolysis
Q9D3A8	Nos1ap	Carboxyl-terminal PDZ ligand of neuronal nitric oxide synthase protein	Cell projection, filopodium	Nitric-oxide synthase binding, postsynaptic actin cytoskeleton organization, regulation of heart rate by chemical signal, eegulation of ventricular cardiac muscle cell membrane repolarization
Q70KF4	Cmya5	Cardiomyopathy-associated protein 5	Nucleus, cytoplasm, cytoplasm perinuclear region, cytoplasm myofibril sarcomere M line, sarcoplasmic reticulum	Identical protein binding, protein phosphatase inhibitor activity, negative regulation of calcineurin-NFAT signaling cascade, regulation of skeletal muscle adaptation
O54724	Cavin1	Caveolae-associated protein 1	Membrane, caveola, cell membrane, microsome, endoplasmic reticulum, cytoplasm, cytosol, mitochondrion, nucleus	Transcription initiation at RNA polymerase I promoter, termination of RNA polymerase I transcription, protein secretion, positive regulation of cell motility, rRNA transcription
Q63918	Cavin2	Caveolae-associated protein 2	Cytoplasm, cytosol	Phosphatidylserine binding, plasma membrane tubulation, positive regulation of transcription by RNA polymerase II
Q9CQ10	Chmp3	Charged multivesicular body protein 3	Cytoplasm, cytosol	Autophagy, autophagosome maturation, late endosome to lysosome transport, multivesicular body-lysosome fusion, viral budding via host ESCRT complex

Uniprot ID	Gene	Short name	Subcellular location	Gene ontology
Q8CIE6	Copa	Coatomer subunit alpha	Cytoplasm, golgi apparatus membrane	Endoplasmic reticulum to Golgi vesicle-mediated transport, intracellular protein transport, protein localization to axon, protein localization to cell leading edge, hormone activity
Q6A0A9	FAM120A	Constitutive coactivator of PPAR-gamma-like protein 1	Cytoplasm, cell membrane	RNA binding
P11440	Cdk1	Cyclin-dependent kinase 1	Nucleus, cytoplasm, cytosol, mitochondrion, cytoskeleton	Apoptotic process, cell division, chromosome condensation, regulation of circadian rhythm, response to toxic substance
Q3U1J4	Ddb1	DNA damage-binding protein 1	Cytoplasm, nucleus	Protein ubiquitination, DNA damage response, regulation of circadian rhythm, positive regulation of protein catabolic process, apoptotic process.
P13705	Msh3	DNA mismatch repair protein Msh3	-	ATP binding, DNA binding, damaged DNA binding, mismatch repair, ATP-dependent DNA damage sensor activity.
P49718	Mcm5	DNA replication licensing factor MCM5	Nucleus, cytoplasm, cytosol	ATP binding, ATP hydrolysis activity, helicase activity, DNA replication origin binding, DNA replication initiation.
Q61881	Mcm7	DNA replication licensing factor MCM7	Nucleus	ATP binding, ATP hydrolysis activity, DNA helicase activity, DNA replication initiation, DNA damage response.
Q61510	Trim25	E3 ubiquitin/ISG15 ligase TRIM25	Nucleus, cytoplasm	Ubiquitin protein ligase activity, transcription coactivator activity, antiviral innate immune response, innate immune response, response to oxidative stress.
P46935	Nedd4	E3 ubiquitin-protein ligase NEDD4	Nucleus, cytoplasm	Ubiquitin protein ligase activity, protein ubiquitination, DNA damage response, innate immune response, regulation of synapse organization.
Q9WVK4	Ehd1	EH domain-containing protein 1	Endosome membrane	ATP binding, GTP binding, endocytosis, endosomal transport, cilium assembly.
P70372	Elavl1	ELAV-like protein 1	Nucleus, cytoplasm	RNA binding, mRNA binding, mRNA stabilization, post-transcriptional gene silencing, positive regulation of autophagy.
Q7TT37	Elp1	Elongator complex protein 1	Nucleus, cytoplasm	tRNA wobble uridine modification.
Q8BGE5	Fancm	Fanconi anemia group M protein homolog	Nucleus	ATP binding, ATP hydrolysis activity, DNA binding, chromatin binding, interstrand cross-link repair.
Q6R0H7	Gnas	Guanine nucleotide-binding protein G(s) subunit alpha isoforms XLas	Cell membrane	GTP binding, GTPase activity, G protein activity, adenylate cyclase activator activity, G protein-coupled receptor signaling pathway.
Q8CI11	Gnl3	Guanine nucleotide-binding protein-like 3	Nucleus, nucleolus	GTP binding, regulation of cell population proliferation, stem cell population maintenance.
Q9CY66	Gar1	H/ACA ribonucleoprotein complex subunit 1	Nucleus, nucleolus	Telomere maintenance via telomerase, pseudouridine synthesis, rRNA processing, telomerase RNA binding, snoRNA binding.

Uniprot ID	Gene	Short name	Subcellular location	Gene ontology
Q9D0E1	Hnrnpm	Heterogeneous nuclear ribonucleoprotein M	Nucleus	Alternative mRNA splicing via spliceosome, RNA binding, protein domain specific binding, nuclear matrix, synapse.
P10922	H1-0	Histone H1.0	Nucleus	DNA binding, nucleosome assembly, chromatin DNA binding, heterochromatin formation, transcription repression.
Q64475	H2bc3	Histone H2B type 1-B	Nucleus	DNA binding, protein heterodimerization activity, structural constituent of chromatin
P62806	H4c1	Histone H4	Nucleus	DNA binding, protein heterodimerization activity, structural constituent of chromatin, chromatin organization, nucleosome assembly
Q03963	Eif2ak2	Interferon-induced, double-stranded RNA-activated protein kinase	Nucleus, cytoplasm	Antiviral innate immune response, response to virus, negative regulation of apoptotic process, positive regulation of NF-kappaB transcription factor activity, protein kinase activity.
Q5DTN8	Jakmip3	Janus kinase and microtubule-interacting protein 3	Golgi apparatus	Kinase binding, microtubule binding
P11679	Krt8	Keratin, type II cytoskeletal 8	Nucleus, cytoplasm	Cell differentiation involved in embryonic placenta development, extrinsic apoptotic signaling pathway, hepatocyte apoptotic process, tumor necrosis factor-mediated signaling pathway, response to other organism.
P62717	Rpl18a	Large ribosomal subunit protein eL20	Cytoplasm	Postsynaptic density, structural constituent of ribosome, cytoplasmic translation.
P61358	Rpl27	Large ribosomal subunit protein eL27	Cytoplasm	Estructural constituent of ribosome, cytoplasmic translation, rRNA processing, translation at postsynapse, translation at presynapse.
P62889	Rpl30	Large ribosomal subunit protein eL30	Cytoplasm	Structural constituent of ribosome, cytoplasmic translation, RNA binding, antimicrobial humoral immune response mediated by antimicrobial peptide
Q9JJI8	Rpl38	Large ribosomal subunit protein eL38	Cytoplasm	Structural constituent of ribosome, cytoplasmic translation, regulation of translation, translation at postsynapse, translation at presynapse.
Q9CPR4	Rpl17	Large ribosomal subunit protein uL22	Cytoplasm	Structural constituent of ribosome, cytoplasmic translation, translation at postsynapse, translation at presynapse, positive regulation of G1/S transition of mitotic cell cycle.
Q9CXW4	Rpl11	Large ribosomal subunit protein uL5	Nucleus, nucleolus, cytoplasm	Structural constituent of ribosome, cytoplasmic translation, positive regulation of gene expression, protein stabilization, ribosomal large subunit assembly.
Q8BMJ2	Lars1	Leucine--tRNA ligase, cytoplasmic	Cytoplasm	Aminoacyl-tRNA deacylase activity, glutamine-tRNA ligase activity, leucine-tRNA ligase activity, cellular response to leucine starvation, positive regulation of TORC1 signaling.

Uniprot ID	Gene	Short name	Subcellular location	Gene ontology
P11369	Pol	LINE-1 retrotransposable element ORF2 protein	-	Endonuclease activity, metal ion binding, RNA-directed DNA polymerase activity, DNA recombination.
P08249	Mdh2	Malate dehydrogenase, mitochondrial	Mitochondrion matrix	Malate dehydrogenase activity, aerobic respiration, gluconeogenesis, malate-aspartate shuttle, tricarboxylic acid cycle
Q8BMD7	Morc4	MORC family CW-type zinc finger protein 4	Nucleus	ATP hydrolysis activity, methylated histone binding, nucleoplasm, zinc ion binding
Q99LX5	Mmtag2	Multiple myeloma tumor-associated protein 2 homolog	-	-
Q6DFW4	Nop58	Nucleolar protein 58	Nucleus, nucleolus	ATPase binding, snoRNA binding, ribosomal small subunit biogenesis, snoRNA localization, TFIID-class transcription factor complex binding
Q9JIK5	Ddx21	Nucleolar RNA helicase 2	Nucleus, nucleolus, cytoplasm	Chromatin remodeling, defense response to virus, positive regulation of transcription by RNA polymerase II
Q9QYX7	Pclo	Protein piccolo	Presynaptic active zone	Cell projection, postsynaptic density, presynaptic active zone, synapse, calcium ion binding
Q6NS46	Pdcd11	Protein RRP5 homolog	Nucleus, nucleolus	NF-kappaB binding, nucleic acid binding, rRNA processing
Q8CBY1	Samd4a	Protein Smaug homolog 1	Cytoplasm	RNA binding, translation repressor activity, positive regulation of translation, regulation of mRNA stability
Q8BVY0	Rsl1d1	Ribosomal L1 domain-containing protein 1	Nucleus, nucleolus	mRNA 3'-UTR binding, mRNA 5'-UTR binding, regulation of apoptotic process, regulation of cellular senescence, regulation of protein localization
P97473	Tarbp2	RISC-loading complex subunit TARBP2	Cytoplasm	Global gene silencing by mRNA cleavage, miRNA processing, positive regulation of translation, regulation of viral transcription, skeletal muscle tissue regeneration
Q8CGC6	Rbm28	RNA-binding protein 28	Nucleus, nucleolus	Spliceosomal complex, RNA binding, mRNA processing, RNA splicing, pre-mRNA splicing
Q99LF4	Rtcb	RNA-splicing ligase RtcB homolog	Nucleus, cytoplasm	tRNA splicing via endonucleolytic cleavage and ligation, RNA ligase (GTP) activity, GTP binding, metal ion binding, in utero embryonic development
P35550	Fbl	rRNA 2'-O-methyltransferase fibrillarin	Nucleus, nucleolus	ATPase binding, histone H2AQ104 methyltransferase activity, RNA binding, ribosomal small subunit biogenesis, rRNA methylation
O55222	Ilk	Scaffold protein ILK	Nucleus, cytoplasm, cell membrane, cytoskeleton, centrosome	Cell morphogenesis, cell population proliferation, integrin-mediated signaling pathway, positive regulation of canonical NF-kappaB signal transduction, positive regulation of DNA-templated transcription.

Uniprot ID	Gene	Short name	Subcellular location	Gene ontology
Q6ZWU9	Rps27	Small ribosomal subunit protein eS27	Nucleus, nucleolus, cytoplasm	Structural constituent of ribosome, ribosomal small subunit biogenesis, rRNA processing, translation at postsynapse, translation at presynapse.
P62267	Rps23	Small ribosomal subunit protein uS12	Nucleus, nucleolus, cytoplasm, endoplasmic reticulum	Translation, cytoplasmic translation, maintenance of translational fidelity, ribosomal small subunit biogenesis, stress granule assembly.
Q9D883	U2af1	Splicing factor U2AF 35 kDa subunit	Nucleus	mRNA splicing via spliceosome, RNA binding, catalytic step 2 of spliceosome, RS domain binding, zinc ion binding.
Q8CIT9	Sbsn	Suprabasin	Cytoplasm	-
P70279	Surf6	Surfeit locus protein 6	Nucleus	DNA binding, molecular condensate scaffold activity, RNA binding, ribosome assembly, ribosome biogenesis.
Q6PGB8	Smarca1	SWI/SNF-related matrix-associated actin-dependent regulator of chromatin subfamily A member 1	Nucleus	ATP-dependent chromatin remodeler activity, chromatin binding, positive regulation of DNA-templated transcription, neuron differentiation, regulation of neural precursor cell proliferation
Q91ZW3	Smarca5	SWI/SNF-related matrix-associated actin-dependent regulator of chromatin subfamily A member 5	Nucleus	ACF complex, B-WICH complex, CERF complex, CHRAC, chromatin silencing complex
Q62381	Tll1	Tolloid-like protein 1	-	Calcium ion binding, collagen binding, metalloendopeptidase activity, zinc ion binding, cell differentiation, proteolysis
Q62318	Trim28	Transcription intermediary factor 1-beta	Nucleus	Chromatin, DNA binding, DNA repair, transcription coactivator activity, protein sumoylation
Q61687	Atrx	Transcriptional regulator ATRX	Nucleus	ATP binding, ATP hydrolysis activity, chromatin binding, DNA binding, helicase activity
Q9Z277	Baz1b	Tyrosine-protein kinase BAZ1B	Nucleus	Chromatin organization, chromatin remodeling, DNA damage response, positive regulation of transcription by RNA polymerase II, positive regulation of transcription by RNA polymerase I
Q80X50	Ubap2l	Ubiquitin-associated protein 2-like	Nucleus	Binding of sperm to zona pellucida, hematopoietic stem cell homeostasis, positive regulation of stress granule assembly, stress granule assembly
Q8VDJ3	Hdlbp	Vigilin	Nucleus, cytoplasm	RNA binding, cholesterol metabolic process, lipid transport
